# Aberrant activation of hippocampal astrocytes causes neuroinflammation and cognitive decline in mice

**DOI:** 10.1371/journal.pbio.3002687

**Published:** 2024-07-11

**Authors:** Jae-Hong Kim, Nakamura Michiko, In-Sun Choi, Yujung Kim, Ji-Young Jeong, Maan-Gee Lee, Il-Sung Jang, Kyoungho Suk

**Affiliations:** 1 Department of Pharmacology, School of Medicine, Kyungpook National University, Daegu, Republic of Korea; 2 Brain Science & Engineering Institute, Kyungpook National University, Daegu, Republic of Korea; 3 Brain Korea 21 four KNU Convergence Educational Program of Biomedical Sciences for Creative Future Talents, Kyungpook National University, Daegu, Republic of Korea; 4 Department of Pharmacology, School of Dentistry, Kyungpook National University, Daegu, Republic of Korea; Academia Sinica, TAIWAN

## Abstract

Reactive astrocytes are associated with neuroinflammation and cognitive decline in diverse neuropathologies; however, the underlying mechanisms are unclear. We used optogenetic and chemogenetic tools to identify the crucial roles of the hippocampal CA1 astrocytes in cognitive decline. Our results showed that repeated optogenetic stimulation of the hippocampal CA1 astrocytes induced cognitive impairment in mice and decreased synaptic long-term potentiation (LTP), which was accompanied by the appearance of inflammatory astrocytes. Mechanistic studies conducted using knockout animal models and hippocampal neuronal cultures showed that lipocalin-2 (LCN2), derived from reactive astrocytes, mediated neuroinflammation and induced cognitive impairment by decreasing the LTP through the reduction of neuronal NMDA receptors. Sustained chemogenetic stimulation of hippocampal astrocytes provided similar results. Conversely, these phenomena were attenuated by a metabolic inhibitor of astrocytes. Fiber photometry using GCaMP revealed a high level of hippocampal astrocyte activation in the neuroinflammation model. Our findings suggest that reactive astrocytes in the hippocampus are sufficient and required to induce cognitive decline through LCN2 release and synaptic modulation. This abnormal glial–neuron interaction may contribute to the pathogenesis of cognitive disturbances in neuroinflammation-associated brain conditions.

## Abstract 2

LCN2 is an inflammatory mediator that is upregulated under diverse neuropathological conditions. This study shows that repetitive and sustained stimulation of hippocampal astrocytes induces reactive astrogliosis and subsequent LCN2 release, triggering neuroinflammation, inhibition of NMDA-receptor-related neuronal activity and cognitive impairment.

## Introduction

The hippocampus has a vital role in the establishment and retention of learning and memory. Processing of brain information is traditionally perceived as a neuronal function. In general, it is believed that astrocytes primarily have supportive functions for neurons in the central nervous system (CNS) [1], but increasing evidence suggests that astrocytes exert several additional active functions, including signal transmission, information processing, and regulation of neural and synaptic plasticity [2–4]. Recent studies have also linked astrocytes with various behavioral states and brain pathologies in different animal models and presented evidence that cognitive processing, including learning and memory, requires coordinated interplay between astrocytes and different synaptic ensembles [5–8]. Therefore, the coordinated actions of a glial–neuron network may underlie many brain functions, including cognition. However, there is limited information regarding the physiological contribution of astrocytes to cognitive function and underlying mechanisms.

Numerous studies have previously shown the importance of astrocytes in memory, demonstrating that the disruption of astrocyte function resulted in memory impairment [9–12] and that memory impairment in genetic models of cognitive deficit can be reduced by correcting the genotype of astrocytes [13,14]. Moreover, a recent study demonstrated that activation of the Gq-coupled pathway in astrocytes enhanced the memory of mice [15]. In contrast, a more recent study by Li and colleagues demonstrated that the optogenetic stimulation of hippocampal CA1 astrocytes in a transgenic rat model attenuated the contextual fear memory via adenosine A_1_ receptors [16]. Furthermore, other studies have suggested that memory function may be detrimentally affected by certain astrocytic intracellular pathways [17–19]. Interestingly, these seemingly contradictory results provide the true representation in that the effect of astrocytic activity on memory is reflected as an inverted “U-shaped” function in which an optimal level of astrocytic activity is critical to support intact memory, but either a deficit or excess of activity may be detrimental.

A number of previous studies have demonstrated that nonneuronal cells, mainly microglia and astrocytes, are involved in the pathogenesis of Alzheimer’s disease (AD) as well as other neurodegenerative conditions and disorders [20–22]. Neuroinflammation is a common feature of diverse nervous system pathologies. In many instances, it begins at the early stage of the disease, which lays the foundations for further exacerbation. The main drivers of neuroinflammation are brain-resident glial cells, such as microglia and astrocytes [23]. Microglia are the primary responders to any insult to the brain parenchyma, translating the signals into diverse molecules. These microglia-derived molecules regulate the stimuli-dependent reactivity of astrocytes. Once activated, astrocytes can control the microglia phenotype [24]. Recent evidence indicates that the crosstalk between these glial cells plays an important role in delaying or accelerating neuroinflammation and the overall disease progression [24–26].

It was determined that astrocytes are integral mediators of cognitive impairment [27,28]. Considering the important role of astrocytes in memory impairment, it is possible that physiologically expressed proinflammatory cytokines are involved in memory formation [29–33], but under pathological conditions, excessive cytokine release may lead to overactivation of astrocytes, thereby resulting in neuronal injury and eventual cognitive decline [34,35]. We have previously identified lipocalin-2 (LCN2) as a mediator of reactive astrocytosis [36–39]. Other studies have also implicated LCN2 in neurodegenerative and cognitive disorders displaying neuronal loss, alterations in astrocytes, neuroinflammatory responses, and synaptic and network dysfunction [40,41]. However, a definitive link between LCN2, sustained neuroinflammation in the hippocampus, and cognitive impairment has not yet been established, and it remains unclear whether hippocampal inflammation persists along with chronic reactive astrocytosis. Moreover, LCN2 has been previously reported to exert both anti-inflammatory [42–44] and proinflammatory [45–60] effector functions in different contexts. Whether LCN2 is neurotoxic or neuroprotective is the subject of controversy.

To elucidate the disease mechanisms characterized by inappropriate astrocyte reactivity, it is important to understand the relationship between astrocytic dysfunction, particularly prolonged signaling, and neuronal function. To address this issue, we employed repeated optogenetic stimulation and chemogenetic strategies with a combination of electrophysiology and behavioral assessments to better understand the role of the CA1 astrocytes and effects of aberrant activation in the modulation of hippocampal synaptic activity and cognitive decline.

## Results

### Repeated optogenetic stimulation of hippocampal astrocytes induces cognitive impairment

**Fig 1:**
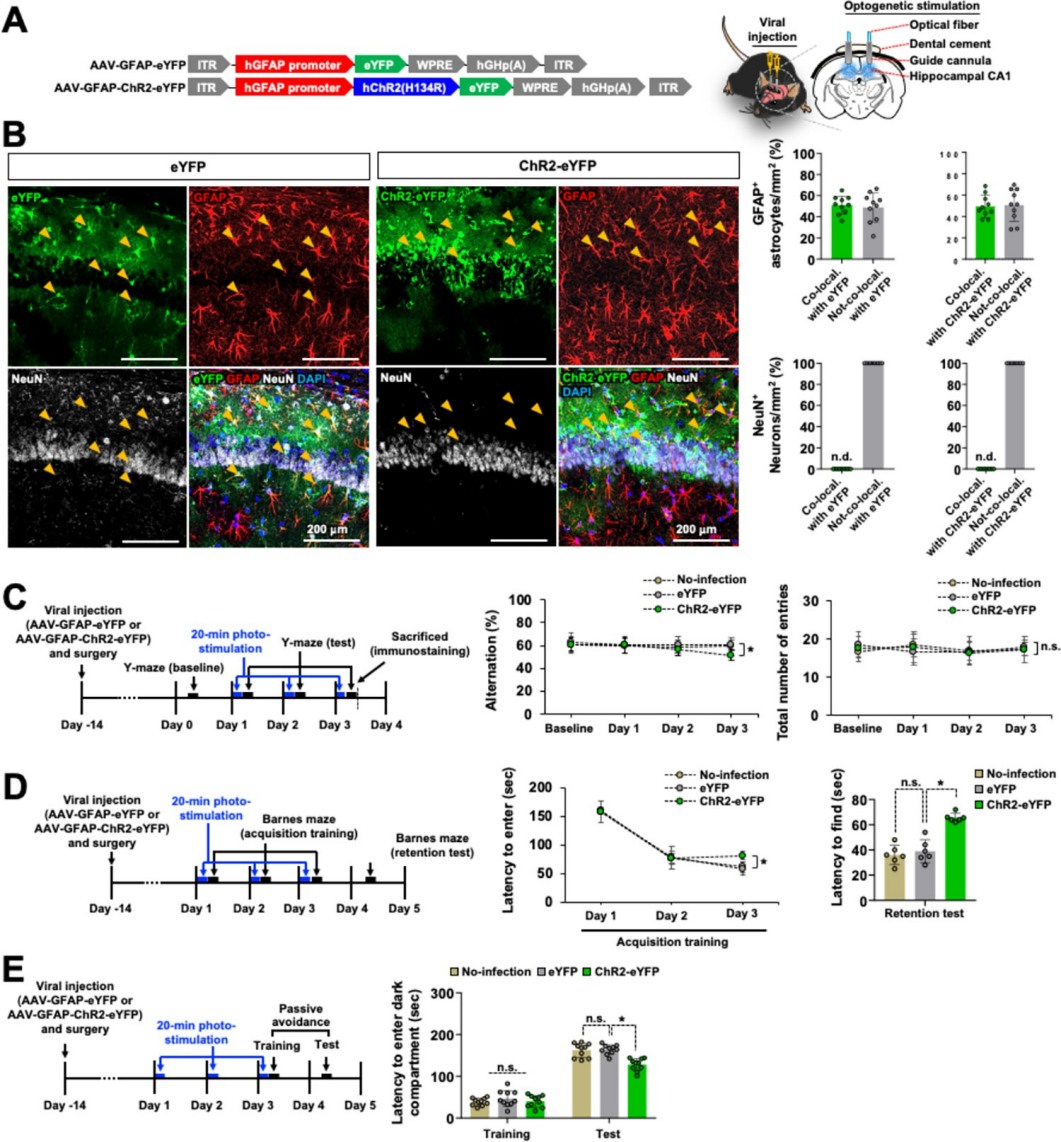
Repeated optogenetic stimulation of hippocampal astrocytes induces cognitive impairment. (**A**) Visual representation of viral construction, cannula, and optical fiber placement site used to stimulate the hippocampal CA1 astrocytes. (**B**) Brain tissue samples were subjected to immunofluorescence analysis to localize the expression of the eYFP (green) and ChR2-eYFP (green) in astrocytes (GFAP, red) and neurons (NeuN, white). Nuclei were stained with DAPI (blue). Arrowheads (yellow) indicate the colocalization of eYFP, ChR2-eYFP, and GFAP. Quantification of eYFP, ChR2-eYFP, GFAP, and NeuN colocalization is shown in the adjacent graphs. Scale bar: 200 μm. Results are expressed as mean ± SEM (*n* = 10). n.d., not detected. (**C**–**E**) Experimental timeline of viral injection, surgery, optogenetic stimulation, and behavioral analysis. Mice were injected with AAV-GFAP-eYFP or AAV-GFAP-ChR2-eYFP. From post-viral injection day 14, the first (day 1), second (day 2), and third (day 3) optogenetic stimulations were delivered for 20 min to the hippocampal CA1 region. Blue and black arrows indicate the time points of optogenetic stimulation and behavioral testing, respectively. The cognitive behavior of eYFP or ChR2-eYFP-expressing mice after the optogenetic stimulation of hippocampal astrocytes was analyzed by Y-maze (*n* = 10) (**C**), Barnes maze (*n* = 6) (**D**), and passive avoidance (*n* = 10) (**E**) tests. Results are expressed as mean ± SEM (*n* = 6 or 10). *p < 0.05 between the indicated groups; n.s., not significant (two-way ANOVA). Source data can be found in S1 Data. AAV, adeno-associated virus.

Previous anatomical studies have indicated that the dorsal hippocampus is important for memory formation and retrieval, whereas its ventral part is essential for emotions [61,62]. To examine the effects of the in situ long-term astrocyte stimulation on cognitive function, dorsal hippocampus CA1 astrocytes were optogenetically stimulated under different time conditions, and their behavior with regards to spatial learning and memory were monitored. For this purpose, mice were intracranially injected (targeting dorsal hippocampus) with an adeno-associated virus (AAV) vector expressing the blue-light-sensitive cation channel (channelrhodopsin 2; ChR2) under the regulation of the astrocyte-specific GFAP promoter (AAV-GFAP-ChR2-eYFP). At 14 days after viral injection, photostimulation was delivered through an optic fiber to the ChR2-eYFP-expressing astrocytes within the hippocampus CA1 region. The hippocampal astrocytes were exposed to the laser-based illumination through the optic fiber (Fig 1A). These animals were subjected to the Y-maze, Barnes maze, and passive avoidance tests to examine their spatial learning and memory performance. After the behavioral tests, the brain tissue was harvested for the histological examination of ChR2 expression. Green fluorescence derived from ChR2-fused eYFP was detected in the CA1 region of the hippocampus in AAV-GFAP-ChR2-eYFP-injected animals after optogenetic stimulation. The cellular distribution of ChR2 expression in the brain sections was characterized by immunofluorescence staining with GFAP (an astrocyte marker), NeuN (a neuronal nucleus marker), and Iba-1 (a microglial marker) antibodies. As anticipated, ChR2 expression was detected in GFAP^+^ astrocytes, but not in the neurons (Fig 1B) or microglia (S1A Fig). A similar pattern of astrocytic ChR2-eYFP expression was observed in the CA1 region of the no-photostimulation control animals (S1B and S1C Fig).

Using this optogenetics setup, we investigated whether the number and duration of optogenetic stimulation had an effect on spatial learning and memory. Spatial learning and memory were evaluated after 1 to 3 times of photostimulation of astrocytes for 20 min. This 20-min photostimulation was administered once-a-day for up to 3 days. Chronic optogenetic stimulation has also been used in a recent study to induce cerebellar Bergman gliosis and excitotoxicity [63]. Photostimulation (1 Hz, 500 ms on and 500 ms off cycle) did not result in significant changes in cognitive performances at day 1 or day 2; however, cognitive performance was significantly decreased at day 3 in the Y-maze test (Fig 1C). We found no significant effects in mice without surgery, surgery without virus injection (S2 Fig), or mice injected with a control viral vector (AAV-GFAP-eYFP) (Fig 1C). Comparatively, the 5- or 10-min photostimulation did not induce any significant alteration in cognitive function over a period of 1 to 3 days (S3 Fig). Motor activity remained unchanged with photostimulation, as determined by the total number of arm entries in the Y-maze test (Figs 1C and S3A). When the photostimulation was repeated 12 times over 12 days, the optogenetic-induced cognitive impairments were not further influenced (S4 Fig).

We also conducted other behavioral tests that rely on spatial information processing by the CA1 hippocampal networks, i.e., the Barnes maze and passive avoidance test. In the Barnes maze platform, mice were trained for 3 consecutive days after photostimulation (20 min), as indicated in Fig 1D. Then, a probe trial was conducted to evaluate spatial memory retention. We observed significantly decreased learning performances in the ChR2-eYFP-expressing group on day 3 during the training period. These mice exhibited a decrease in spatial memory retention compared with the eYFP-expressing control group at 24 h (day 4) after training (Fig 1D). Photostimulation for 5 or 10 min did not cause statistically significant differences in spatial memory (S3B Fig). In the passive avoidance test, similar impaired memory retention was observed in the mice exposed to photostimulation (20 min) for 3 days. The mice were given an electric shock in the dark compartment after photostimulation (20 min) for 3 days in the home cage (at day 3), and then a probe trial was performed without photostimulation after 24 h (at day 4). We detected a significantly decreased latency to enter the dark compartment in the probe test in the ChR2-eYFP-expressing group, thus indicating a decrease in memory retention (Fig 1E). There were no significant differences after photostimulation for 5 or 10 min (S3C Fig). These results suggest that repeated optogenetic stimulation (20-min photostimulation for 3 days) of hippocampal CA1 astrocytes induces hippocampus-dependent cognitive impairment.

Next, to evaluate the effect of astrocytic activation on spatial memory during the acquisition stage, we administered photostimulation for 5 to 20 min during the training session in the Barnes maze task (S5A Fig). Photostimulation for 5 min led to a slight enhancement of memory function by reducing the latency to reach the target hole at day 2 of the training trial, but there was no significant difference at day 3 (S5B Fig), whereas 10-min photostimulation showed no changes (S5C Fig) in spatial learning and memory behavior. These results suggest that the modification of cognitive function by the optogenetic stimulation of hippocampal astrocytes is dependent on the stimulation time. Furthermore, to assess the effect of the astrocytic activation on spatial memory during and after the memory acquisition stage, we administered 20-min photostimulation during (S5D Fig) and after (S6A Fig) the training session in the Barnes maze task. Consistently, both photostimulation paradigms induced cognitive decline in the ChR2-eYFP-expressing mice at day 3 in the Barnes maze task (S5D and S6B Figs), implying that the photostimulation impaired diverse cognitive functions, including learning and memory recall. Moreover, in the passive avoidance test, to assess the effect of astrocytic activation on memory before and after the training (electric shock), we administered 20-min photostimulation after (S7A Fig) and before (S7B Fig) the training session. Consistently, both photostimulation paradigms induced cognitive decline in ChR2-eYFP-expressing mice at day 3 in the passive avoidance test (S7A and S7B Fig), implying that the photostimulation impaired diverse cognitive functions, including memory retention and recall. We found no significant effects in the mice injected with a control viral vector (AAV-GFAP-eYFP). Repeated optogenetic stimulation, such as 20 min/day for 3 days, induced consistent cognitive impairment irrespective of the memory acquisition stage. Therefore, this condition was used to stimulate the ChR2-eYFP-expressing astrocytes in subsequent experiments to explore the mechanism underlying the hippocampal astrocyte-mediated cognitive impairment.

### Repeated optogenetic stimulation of hippocampal astrocytes reduces the LTP of excitatory synaptic transmission

The above-described results show that the repeated optogenetic stimulation of the hippocampal CA1 astrocytes induces memory impairment which suggests a possible interaction between astrocytes and neurons within the CA1 region. Moreover, emerging evidence suggests that LTP is the result of the interaction between astrocytes and synapses in the hippocampus [64,65]. However, abnormal astrocytic signaling can induce or contribute to the synaptic and network imbalances associated with cognitive impairment [66–68]. Thus, we investigated whether the excitatory synaptic transmission is affected by the repeated stimulation of CA1 astrocytes using hippocampal slices, which were prepared from the mice on the last day of optogenetic stimulation (at day 3) (S8A Fig). No significant difference was observed in the input–output relationship and paired-pulse ratio of field excitatory postsynaptic potential (fEPSPs) between the eYFP- and ChR2-eYFP-expressing animals (S8B and S8C Fig). In a whole-cell recording mode, spontaneous miniature excitatory postsynaptic currents (mEPSCs) were recorded at a holding potential of −60 mV in the presence of both 50 μm D-APV and 300 nM TTX, an NMDA receptor antagonist and a specific voltage-gated Na^+^ channel blocker, respectively, from the hippocampal CA1 pyramidal neurons. The frequency and amplitude of mEPSCs showed no difference between the eYFP- and ChR2-eYFP-expressing animals (S8D Fig). Altogether, these results suggest that the basal excitatory synaptic transmission mediated by AMPA receptor subtypes is not affected by the repeated stimulation of astrocytes in both the eYFP- and ChR2-eYFP-expressing groups.

**Fig 2:**
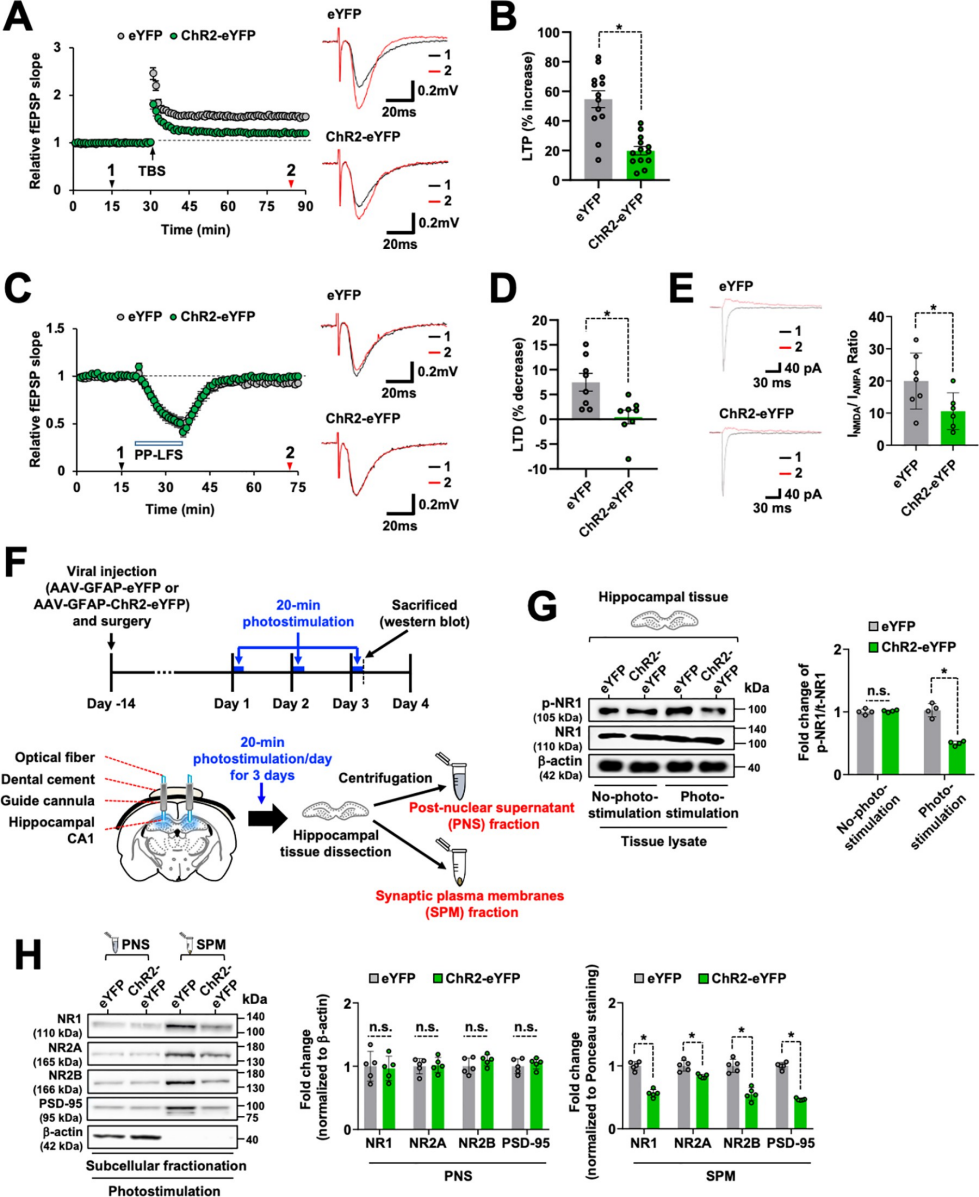
Optogenetic stimulation of the hippocampal CA1 astrocytes reduced the hippocampal long-term synaptic plasticity and expression of the NMDA receptors. (**A**) Time courses of the fEPSP responses before and after TBS from the hippocampal slices in the eYFP (gray circles, *n* = 13 from 10 mice) or ChR2-eYFP-expressing (green circles, *n* = 13 from 10 mice) groups. The values were normalized in each experiment to the mean amplitude value measured during the control period (20–30 min). TBS was applied for LTP induction at 30 min. Typical traces from the average of 6 successive fEPSPs were recorded at the time indicated by the arrowheads with numbered regions (1, black; 2, red). Results are expressed as mean ± SEM (*n* = 13). (**B**) Levels of TBS-induced LTP in the eYFP (*n* = 13) and ChR2-eYFP-expressing (*n* = 13) groups. Results are expressed as mean ± SEM. *p < 0.01 between the indicated groups (unpaired *t* test). (**C**) Time courses of the fEPSP responses before and after PP-LFS from the hippocampal sections in the eYFP (gray circles, *n* = 8 from 5 mice) and ChR2-eYFP-expressing (green circles, *n* = 8 from 5 mice) groups. PP-LFS was applied for LTD induction at 30 min. Insets represent the typical raw traces from the average of 6 successive fEPSPs recorded at the time indicated by the arrowheads with numbered regions (1, black; 2, red). (**D**) PP-LFS-induced LTD in the eYFP (*n* = 8 from 5 mice) and ChR2-eYFP-expressing (*n* = 8 from 5 mice) groups. The mean fEPSP slope 35–40 min after PP-LFS was quantified as the LTD level. Results are expressed as mean ± SEM. *p < 0.01 between the indicated groups (Student’s *t* test). (**E**) Typical traces of the action potential-dependent EPSCs recorded from the hippocampal CA1 neurons in the eYFP (upper) and ChR2-eYFP-expressing (lower) groups. The AMPA receptor-mediated (inward currents, black) and NMDA receptor-mediated (outward currents, red) EPSCs were recorded at a holding potential of −60 and +20 mV, respectively. The amplitude ratio of AMPA and NMDA receptor-mediated currents in the eYFP (*n* = 7) and ChR2-eYFP-expressing (*n* = 6) groups is shown in the adjacent graph. Results are expressed as mean ± SEM. *p < 0.05 between the indicated groups (unpaired *t* test). (**F**) Experimental timeline and scheme for western blot analysis. (**G**) Phospho-NR1 (p-NR1) or total NR1 (NR1) protein levels were measured by western blotting. Quantification of the band intensities is presented in the adjacent graphs. Results are expressed as mean ± SD (*n* = 4). *p < 0.05 between the indicated groups (one-way ANOVA). Visual representation of the cannula and optical fiber placement site used for the optogenetic stimulation of the hippocampal astrocytes, and schematic view of the subcellular fractionation experimental workflow (**F**, bottom). (**H**) Western blot analysis of NR1, NR2A, NR2B, and PSD-95 present in subcellular fractions (PNS and SPM) obtained from the hippocampal tissues of the eYFP and ChR2-eYFP-expressing mice groups. Quantification of NR1, NR2A, NR2B, or PSD-95 in the PNS was based on the normalization against the internal control β-actin, and that of NR1, NR2A, NR2B, or PSD-95 in the SPM was normalized against the internal control Ponceau S and represented as graphs for the blots. The Ponceau S-stained membranes are shown in S13A Fig. Results are expressed as mean ± SD (*n* = 5). *p < 0.05 between the indicated groups; n.s., not significant (one-way ANOVA). Source data can be found in S1 Data. fEPSP, field excitatory postsynaptic potential; LTD, long-term depression; LTP, long-term potentiation; PNS, postnuclear supernatant; PP-LFS, paired-pulse low-frequency stimulation; SPM, synaptic plasma membrane; TBS, theta burst stimulation.

Therefore, we next examined whether there were changes in the long-term synaptic plasticity, such as long-term potentiation (LTP) and long-term depression (LTD), in the ChR2-eYFP-expressing group. Theta burst stimulation (TBS) induced a significant LTP of the excitatory synaptic transmission (20-min stimulation, 54.7 ± 5.7% increase, *n* = 13) in the control group; however, the magnitude of TBS-induced LTP was significantly reduced in the ChR2-eYFP-expressing group (20-min stimulation, 19.9 ± 2.8% increase, *n* = 13, *p* < 0.01, Fig 2A and 2B). However, the extent of TBS-induced LTP alteration was not significantly different in ChR2-eYFP-expressing group after 5-min photostimulation (S9 Fig), which is consistent with the behavioral data (S3 Fig). We performed additional behavioral experiments to determine the possibility of memory enhancement after 5-min photostimulation during the memory acquisition stage (S10A Fig). We confirmed that the effect of photostimulation for 5 min led to a slight enhancement of memory function at day 2 of the training trial in spatial learning and memory behavior (S10B Fig). We performed additional microdialysis and ELISA to measure the LCN2 protein levels in the dialysate samples of the hippocampal CA1 region after 5-min photostimulation. Under this training condition, no significant differences were detected in the LCN2 protein levels by ELISA (S10C Fig). Although no statistically significant changes were observed in the LTP levels between the eYFP control and ChR2-eYFP-expressing groups (*p* = 0.086), the average LTP value was slightly increased in the ChR2-eYFP-expressing group after 5-min photostimulation during the memory acquisition stage (S10D and S10E Fig). Furthermore, while paired-pulse low-frequency stimulation (PP-LFS) induced a significant LTD of the excitatory synaptic transmission (7.5 ± 1.8% decrease, *n* = 8) in the control group, the magnitude of PP-LFS-induced LTD was significantly reduced in the ChR2-eYFP-expressing group (0.5 ± 1.4% decrease, *n* = 8, *p* < 0.01, Fig 2C and 2D). Because both the LTP and LTD of the hippocampal CA1 area are largely dependent on the activation of postsynaptic NMDA receptors [69–71], we investigated whether there were changes in the NMDA receptor functions in hippocampal CA1 pyramidal neurons after repeated 20-min photostimulation. We observed a significantly lower ratio of NMDA- and AMPA-receptor-mediated EPSCs (NMDA/AMPA ratio) in the ChR2-eYFP-expressing group than in the control groups (19.9 ± 8.6%, *n* = 7, for the eYFP control group, and 10.5 ± 5.7%, *n* = 6, for the ChR2-eYFP group, *p* < 0.05, Fig 2E). These findings suggest that the impaired function of NMDA receptors is involved in the photostimulation-induced decrease in cognitive functions.

Previous research has established that the activation of NMDA receptors in the hippocampus plays a vital role in learning and memory and is associated with an increase in neuronal activity [72]. Therefore, we evaluated the levels of phospho-NR1 (p-NR1) in the hippocampus after optogenetic stimulation (Fig 2F). The results of the western blotting of the hippocampal tissues after photostimulation for 3 days showed significantly decreased p-NR1 levels in the hippocampus compared with those of the control animals (Fig 2G). Total NR1 protein levels remained unchanged in the hippocampus after photostimulation. We further examined the expression levels of NMDA receptor subunits in the synaptic plasma membranes (SPMs) fraction of hippocampal tissues by western blotting (Fig 2F, bottom). After photostimulation, the levels of NR1, NR2A, and NR2B subunits were significantly decreased in the SPM, but not in the postnuclear supernatant (PNS) fraction (Fig 2H). In addition, photostimulation did not alter the expression levels of AMPA receptor subunits in either the tissue lysates or subcellular fractions (S11 Fig). These data suggest that the optogenetic stimulation of astrocytes decreases neuronal activity in the hippocampus by affecting the synaptic membrane localization of NMDA receptor subunits and without altering the number of AMPA receptors or any ion channel properties. In contrast to the 20-min photostimulation, 5- or 10-min photostimulation did not induce significant changes in the p-NR1 or NMDAR expression in the ChR2-eYFP-expressing groups (S12A Fig).

In a previous study, it was observed that the activation of postsynaptic NMDA receptors triggered complex multicomponent signaling pathways that can cause persistent changes in synaptic strength, such as LTP [73]. Therefore, we investigated whether the postsynaptic density (PSD) marker was altered in the hippocampus by the optogenetic stimulation. Results of this western blot analysis demonstrated that the PSD-95 protein levels were also decreased in the SPM of hippocampal tissues (Fig 2H), but there was no difference in the levels of excitatory presynaptic protein maker VGLUT2 (S11C Fig). The SPM was validated using a cytosolic marker β-actin. We found that the SPM was free of cytosolic components (Fig 2H). Ponceau staining was used to confirm the equal loading of samples in the western blot analysis (S13A Fig). These data suggest that the decrease in the NMDA receptor subunit levels in the synaptic membrane is associated with a decrease in NMDA receptor functionality after repeated stimulation of hippocampal astrocytes. An important determinant of the function and activity of the NMDAR involves their subcellular localization [74–76]. We measured the subcellular localization of NMDA receptors in the subcellular fractionated samples of the hippocampal tissue after 20-min photostimulation. In the western blot analysis, we found a decrease in the NMDAR levels in the SPM and an increase in cytosol and light membranes (S2) fraction at day 3 after repeated photostimulation. This indicates a decrease in surface membrane NMDARs and an increase in NMDARs in the intracellular organelle membranes and cytosol (S12A Fig). However, 20-min photostimulation did not alter mRNA levels of NMDAR in the hippocampal tissue (S12B Fig). Taken together, our results suggest that optogenetic stimulation induces the internalization of NMDARs.

### Optogenetic stimulation of CA1 astrocytes induces LCN2 release in the hippocampus

**Fig 3:**
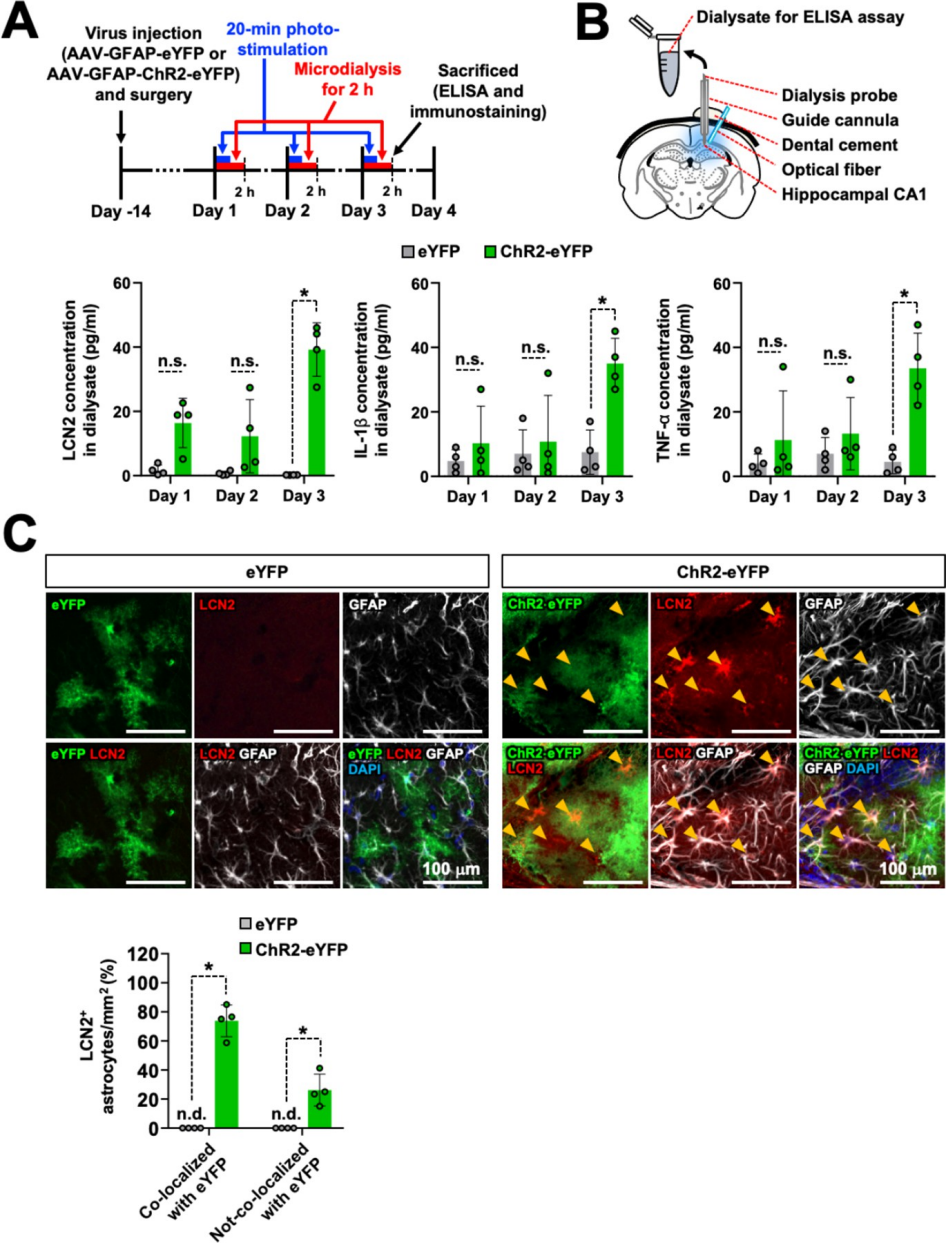
Optogenetic stimulation of hippocampal astrocytes induces extracellular LCN2 and cytokine release. (**A**) Experimental timeline. (**B**) Visual representation of in vivo microdialysis and subsequent ELISA. LCN2, IL-1β, and TNF-α levels in dialysate measured by ELISA. Results are expressed as mean ± SEM (*n* = 4). *p < 0.05 between the indicated groups; n.s., not significant (one-way ANOVA). (**C**) Brain tissue samples were subjected to immunofluorescence analysis to localize the expression of LCN2 (red), eYFP (green), and ChR2-eYFP (green) in astrocytes (GFAP, white). The nuclei were stained with DAPI (blue). Arrowheads (yellow) indicate the colocalization of LCN2, ChR2-eYFP, and GFAP. The quantification of colocalization is shown in the adjacent graph. Scale bar: 100 μm. Results are expressed as mean ± SEM (*n* = 4). *p < 0.05 between the indicated groups (one-way ANOVA). n.d., not detected. Source data can be found in S1 Data. ELISA, enzyme-linked immunosorbent assay.

To explore whether optogenetic stimulation triggers LCN2 release in the hippocampal CA1 region, we collected interstitial fluid from this region by microdialysis and subjected it to LCN2 ELISA analysis (Fig 3A). Microdialysis samples were collected from the hippocampus during 3 days of photostimulation of AAV-ChR2-eYFP-injected mice. After photostimulation for 20 min, the dialysate collected on day 3 showed significantly increased levels of LCN2 and cytokine (Fig 3B). We determined the cellular localization of LCN2 expression in the hippocampus after optogenetic stimulation by immunostaining for LCN2 and GFAP in the brain sections collected after photostimulation. We detected the colocalization of LCN2 protein with GFAP-positive astrocytes (Fig 3C). These results indicate that stimulated astrocytes release LCN2 and proinflammatory cytokines in the hippocampal CA1 region, concurrent with the induction of neuroinflammation and cognitive impairment.

Additional in vitro experiments were conducted using cultured astrocytes, which provided further evidence supporting the important role of astrocyte-derived LCN2 in hippocampal neuroinflammation. The production of *Lcn2* and proinflammatory mediators in vivo after the optogenetic stimulation of hippocampal CA1 astrocytes was corroborated by the optogenetic experiments conducted using cultured astrocytes (S14A and S14B Fig). Optogenetic stimulation of the cultured astrocytes resulted in significantly elevated mRNA expression of *Lcn2*, *Il1b*, and *Tnf* (S14C Fig). LCN2 protein production was also significantly increased in the astrocyte culture media following optogenetic stimulation (S14D Fig). On the other hand, the 20-min photostimulation of the cultured astrocytes expressing ChR2 had no significant impact on the cell viability (S14E Fig).

### Recombinant LCN2 protein reduces the surface expression of NMDA receptor subunits and their signaling in hippocampal neurons and inhibits LTP in hippocampal slices

**Fig 4:**
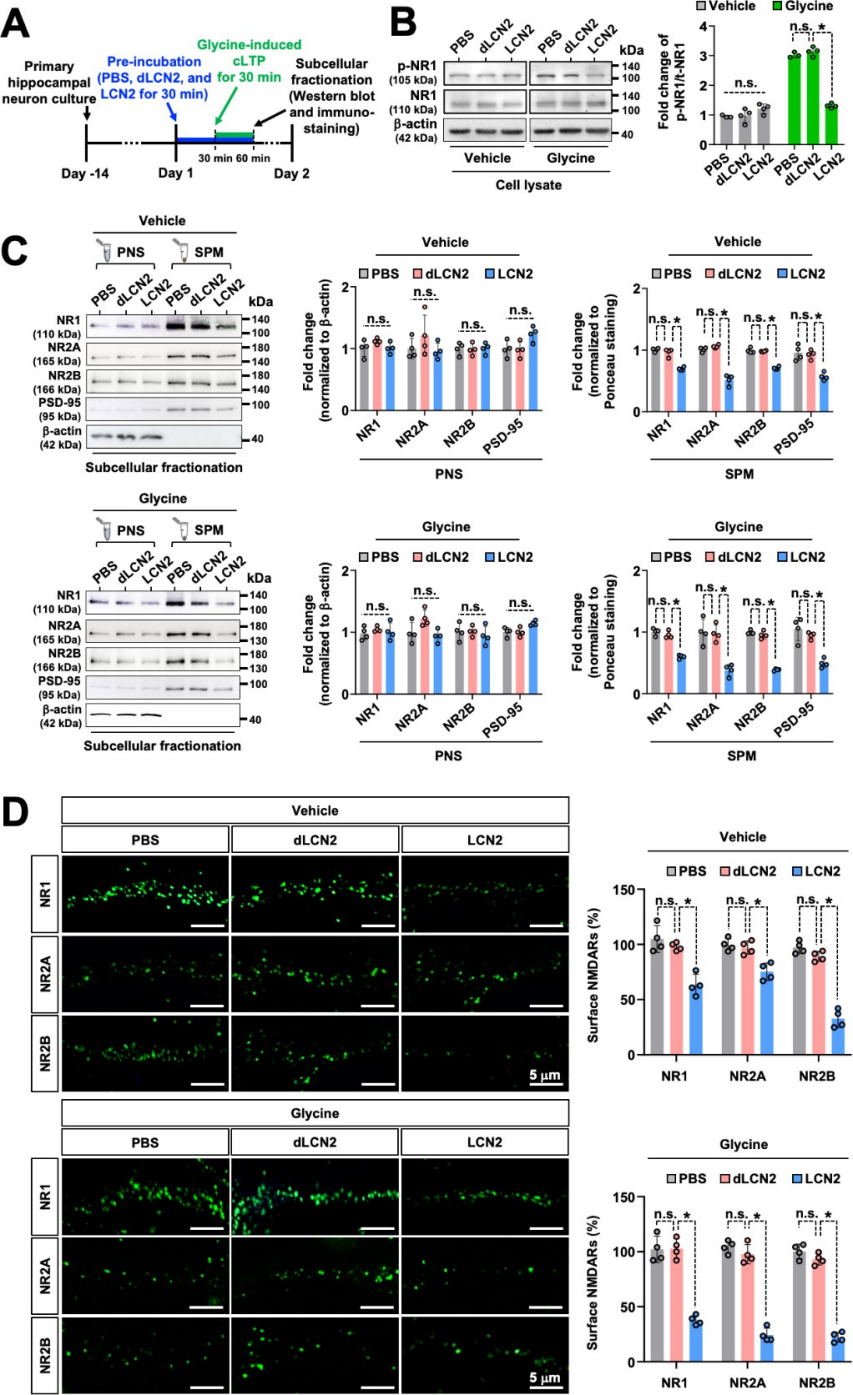
LCN2 treatment reduces membrane expression of NMDA receptors in the hippocampal neurons. (**A**) Experimental timeline. (**B**) Phospho-NR1 (p-NR1) or total NR1 (NR1) protein levels were measured by western blotting. The glycine-induced cLTP method was used to mimic physiological LTP. Cultured hippocampal neurons were stimulated with the vehicle or glycine (200 μm) after incubation with PBS, denatured LCN2 (dLCN2, 10 ng/ml), or LCN2 (1 ng/ml) protein. Results are expressed as mean ± SD (*n* = 3 or 4). *p < 0.05 between the indicated groups; n.s., not significant (one-way ANOVA). (**C**) Western blot analysis of NR1, NR2A, NR2B, and PSD-95 present in the PNS and SPM obtained from hippocampal neurons under each condition. Quantification for NR1, NR2A, NR2B, or PSD-95 in PNS was based on normalization against the internal control β-actin, and for NR1, NR2A, NR2B, or PSD-95 in SPM was normalized against the internal control Ponceau S and represented as graphs for the blots. The Ponceau S-stained membranes were shown in S13D Fig. Results are expressed as mean ± SD (*n* = 4). *p < 0.05 between the indicated groups; n.s., not significant (one-way ANOVA). (**D**) Confocal microscope images depicting the surface immunostaining without cell membrane permeabilization for NR1, NR2A, and NR2B in the cultured hippocampal neurons treated as indicated. Quantification of immunostaining of the surface NR1, NR2A, and NR2B levels. The percentage of surface NMDA receptors in the vehicle- or glycine-treated group was based on each PBS-treated group. Scale bar: 5 μm. Results are expressed as mean ± SD (4 cells). *p < 0.05 between the indicated groups (one-way ANOVA). Source data can be found in S1 Data. cLTP, chemical long-term potentiation; LCN2, lipocalin-2; LTP, long-term potentiation; PNS, postnuclear supernatant; SPM, synaptic plasma membrane.

Based on our finding that optogenetic stimulation of hippocampal astrocytes negatively influences LTP and NMDA receptor signaling, we used primary hippocampal neuronal cultures to further elucidate the role of LCN2 in this phenomenon. We exposed the primary hippocampal neurons to recombinant LCN2 protein and performed western blot analysis to measure the levels of p-NR1 (Fig 4A). To mimic physiological LTP, the glycine-induced chemical LTP (cLTP) method was used because it specifically stimulates the NMDA receptors only at synapses receiving spontaneous release of glutamate, thereby reproducing stimulus-induced synaptic potentiation [77]. LTP induction requires the activation of NMDA receptors and a subsequent rise in the intracellular Ca^2+^ concentration [78,79]. Pretreatment of hippocampal neurons with recombinant LCN2 protein for 30 min reduced the levels of cLTP-related p-NR1 (Fig 4B). Western blot analysis revealed that the amount of detectable NR1 protein in the cell lysates was equivalent among all samples. Treatment with recombinant LCN2 protein decreased the cLTP-related intracellular Ca^2+^ transients in the cultured hippocampal neurons (S15 Fig).

Different NMDA receptor subunits have been implicated in several forms of synaptic plasticity [80]. NMDA receptors are tetrameric, or possibly pentameric, complexes containing at least 1 NR1 subunit and 2 or more NR2 subunits [81], and there is widespread agreement that LTP is regulated by the postsynaptically located NMDA receptors [82–84], although exceptions exist [64]. We evaluated whether LCN2 affects the expression of NMDA receptor subunits in the hippocampal neurons and observed that treatment with recombinant LCN2 protein decreased the expression of NR1, NR2A, and NR2B in the SPM of hippocampal neurons (Fig 4C). Treatment with LCN2 protein also reduced the expression levels of the postsynaptic marker protein PSD-95 (Fig 4C) and Homer (S16A and S16B Fig) in the SPM of cultured hippocampal neurons; however, the expression of the presynaptic marker VGLUT2 was found to be unchanged (S16B Fig). There were no significant differences in the protein levels of NMDA receptor subunits, postsynaptic density proteins (Fig 4C), or presynaptic protein VGLUT2 (S16B Fig) in the PNS. Surface expression levels of NMDA receptor subunits and postsynaptic density proteins were decreased with LCN2 pretreatment in both vehicle- and glycine-treated hippocampal neurons. Glycine can potentiate NMDA receptor-mediated currents through its high-affinity binding with NMDA receptors and produce or facilitate LTP of the AMPA-subtype of glutamate receptor-mediated EPSCs [77]. First, we confirmed that the glycine treatment significantly increases the levels of AMPA receptors in the SPM (S16C Fig), consistent with a previous report [77]. Next, we evaluated the expression levels of AMPA receptor subunits following LCN2 treatment. There were no significant differences in the protein levels of AMPA receptor subunits (GLUR1 and GLUR2) in the SPM or PNS of cultured hippocampal neurons following LCN2 treatment (S16B Fig). We detected the LCN2-mediated reduction of NMDA receptor subunits and postsynaptic density proteins in the neuronal synaptic membrane, which was irrespective of glycine-mediated cLTP induction. Treatment of hippocampal neurons with LCN2 protein (1 ng/ml) had no significant impact on neurite length (S17A Fig) or cell viability (S17B Fig), suggesting that the effects of LCN2 on NMDA receptors and related events were not due to the compromise of neuronal morphology or health. Denatured recombinant LCN2 protein (dLCN2, 10 ng/ml) was used as a negative control, and western blot analysis was performed using the same amounts of protein in PNS and SPM (S13D Fig). Immunostaining analysis of the cultured hippocampal neurons showed that LCN2 decreased the neuronal surface expression of NMDA receptor subunits (Fig 4D). Altogether, these results indicate that LCN2 down-regulates the synaptic membrane expression of NMDA receptor subunits or postsynaptic density proteins in hippocampal neurons.

**Fig 5:**
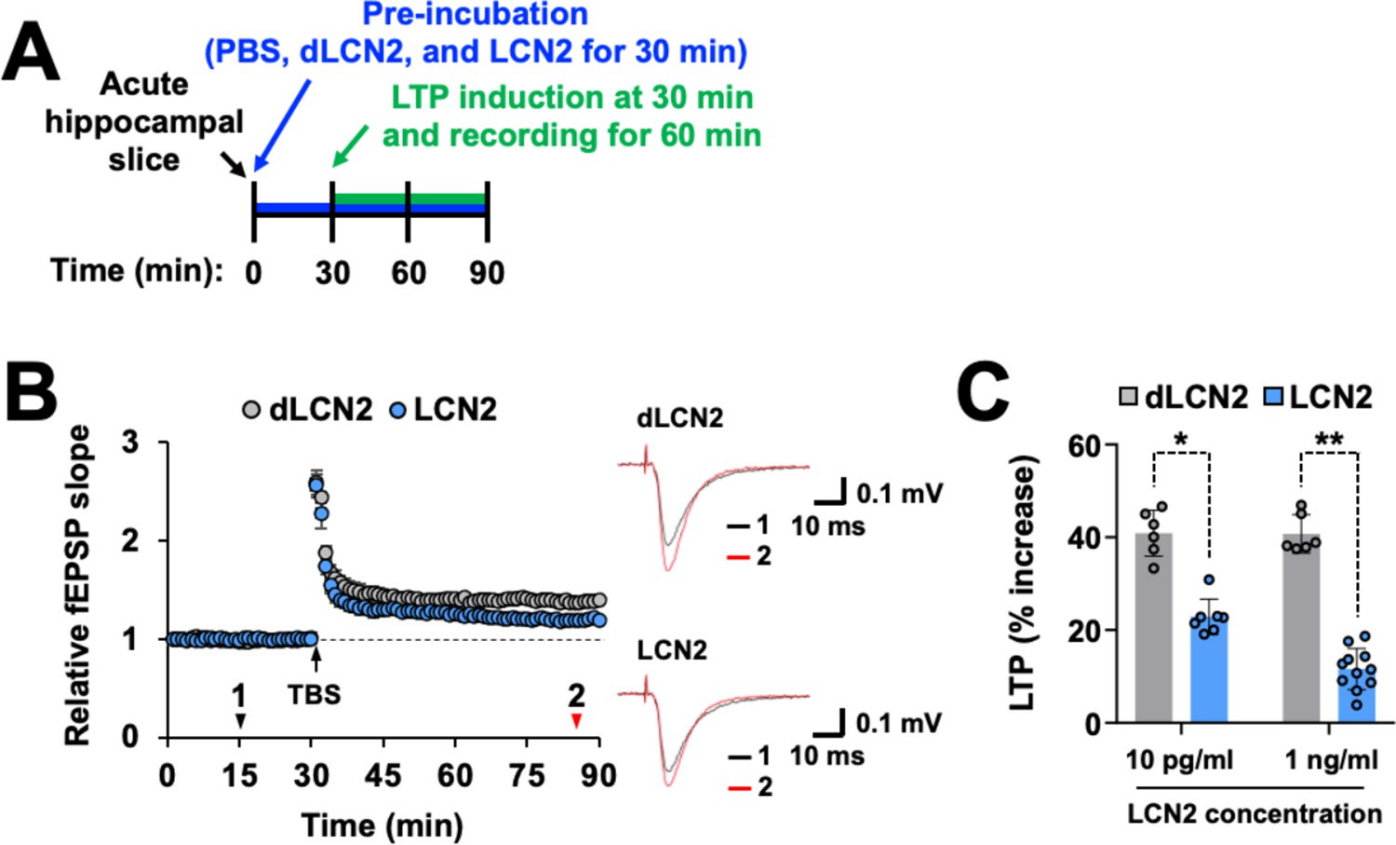
LCN2 treatment decreases the extent of hippocampal LTP in brain slices. **(A)** Experimental timeline. (**B**) Time courses of fEPSP responses before and after TBS from the hippocampal slices treated with denatured LCN2 (dLCN2) (10 pg/ml, gray circles, *n* = 6) and LCN2 (10 pg/ml; blue circles, *n* = 7). Hippocampal slices were pretreated with LCN2 or denatured LCN2 for 30 min before TBS. The values were normalized in each experiment to the mean amplitude value measured during the control period (20–30 min). LTP induction was applied at 30 min. Typical traces averaged from six successive fEPSPs recorded at the time indicated by arrowheads with numbered regions (1, black; 2, red). Results are expressed as mean ± SEM. (**C**) The extent of LTP induced by TBS from the hippocampal slices in the presence of dLCN2 (10 pg/ml, *n* = 6; 1 ng/ml, *n* = 6) or LCN2 (10 pg/ml, *n* = 7; 1 ng/ml, *n* = 11). Results are expressed as mean ± SEM. *p < 0.05, **p < 0.01 between the indicated groups (unpaired *t* test). Source data can be found in S1 Data. fEPSP, field excitatory postsynaptic potential; LCN2, lipocalin-2; LTP, long-term potentiation; TBS, theta burst stimulation.

Based on the above-described results showing that LCN2 down-regulates the expression of NMDA receptors and p-NR1, we next examined whether LCN2 has a direct effect on the long-term synaptic plasticity of excitatory transmission. The treatment of hippocampal slices with recombinant LCN2 protein had a minor effect on the basal excitatory transmission because treatment with LCN2 protein (10 pg/ml, 30 to 90 min) did not change the fEPSP slope (98.5 ± 0.2% of the control, *n* = 7, *p* = 0.40). However, under the continued presence of the recombinant LCN2 protein (Fig 5A), the levels of TBS-induced LTP were significantly lower in the LCN2-treated group than in the control (dLCN2-treated) group (22.8 ± 3.8% increase in the LCN2 group (10 pg/ml), *n* = 6, and 40.8 ± 4.9% increase in the dLCN2 group (10 pg/ml), *n* = 7, *p* < 0.05, Fig 5B and 5C). Pretreatment with LCN2 protein (1 ng/ml) further decreased the levels of TBS-induced LTP (Fig 5C). These results suggest that LCN2 could impair long-term synaptic plasticity, without affecting the basal excitatory transmission, by down-regulating the levels of p-NR1, NMDA receptor subunits, PSD-95, and Homer in the hippocampal synapses.

### Repeated optogenetic stimulation induces neuroinflammation and reactive astrocytes in the hippocampus

**Fig 6:**
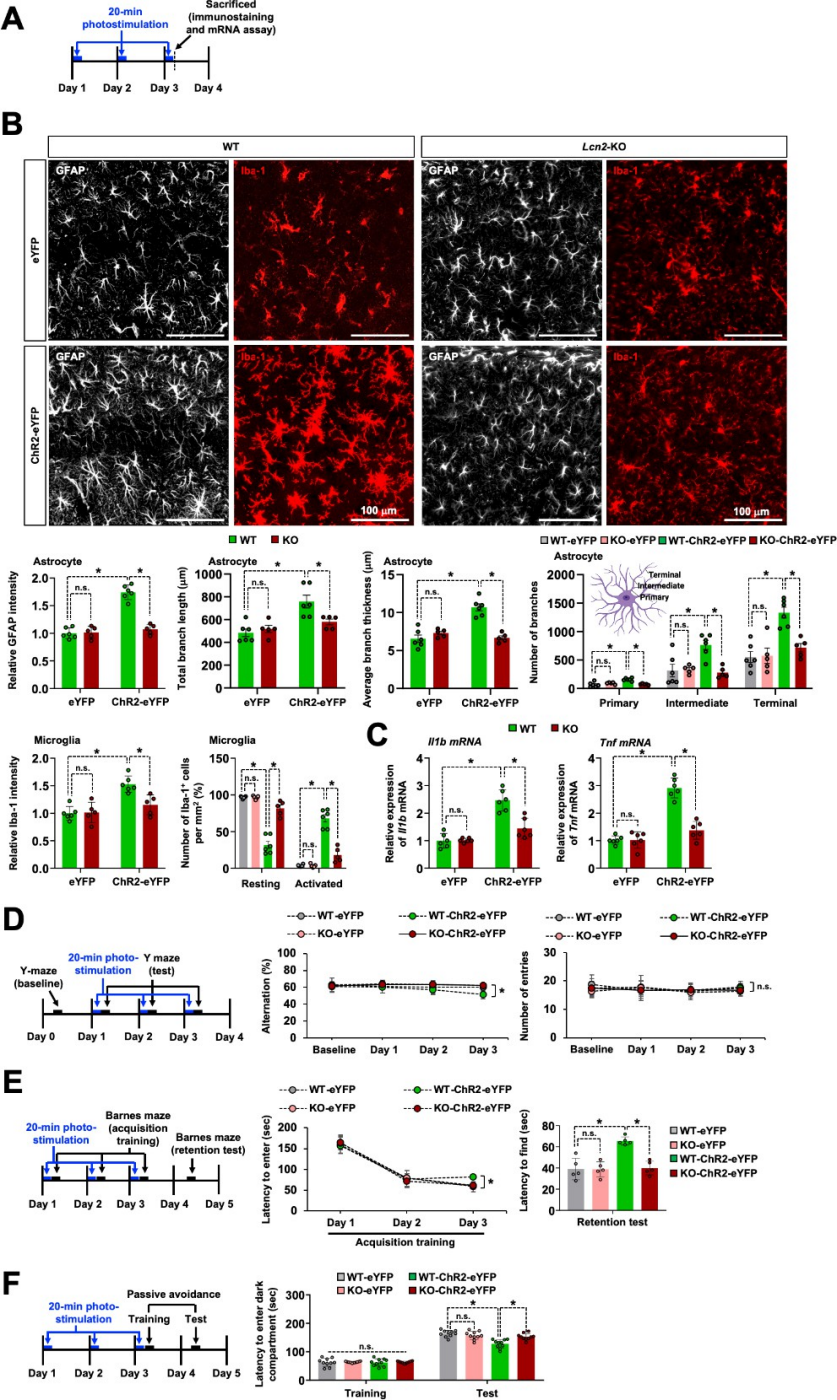
*Lcn2* deficiency attenuates neuroinflammation and cognitive impairment induced by optogenetic stimulation of hippocampal astrocytes. (**A**) Experimental timeline. (**B**) Astrocytes and microglia were identified using GFAP and Iba-1 immunolabeling after optogenetic stimulation. The adjacent graph displays the quantification of fluorescence intensity (astrocytes, left; microglia, middle) and percentage of microglia displaying resting and activated morphology (right) in the hippocampal CA1 region of WT and *Lcn2*-KO mice. In further morphological analysis, the total length and average thickness of astrocyte processes in each group were assessed. The total number of primary, intermediate, and terminal branches for each astrocyte was also measured and compared among experimental groups. Scale bar: 100 μm. Results are expressed as mean ± SEM (*n* = 5 or 6). *p < 0.05 between the indicated groups; n.s., not significant (one-way ANOVA). (**C**) Total mRNA was extracted from the hippocampal tissues of each group and subjected to qPCR to determine the expression levels of *Il1b* and *Tnf* mRNA. *Gapdh* was used as an internal control. Results are expressed as mean ± SEM (*n* = 6). *p < 0.05 between the indicated groups; n.s., not significant (one-way ANOVA). (**D**–**F**) Y-maze (*n* = 10) (**D**), Barnes maze (*n* = 5) (**E**), and passive avoidance (*n* = 10) (**F**) cognitive behavior of eYFP or ChR2-eYFP-expressing WT or *Lcn2*-KO mice after 20-min photostimulation. Results are expressed as mean ± SEM (*n* = 5 or 10). *p < 0.05 between the indicated groups; n.s., not significant (two-way ANOVA). Source data can be found in S1 Data. LCN2, lipocalin-2.

Neuroinflammation is a common feature of virtually every CNS disease and is being increasingly recognized as a potential mediator of cognitive impairment [85]. After the AAV-mediated delivery, the expression of ChR2-eYFP in GFAP^+^ astrocytes was confirmed in the hippocampal sections of these animals (S18 Fig). After the optogenetic stimulation of these animals, we evaluated the glial activation and proinflammatory cytokine expression in the hippocampus (Fig 6A). Immunofluorescence analysis of the hippocampal CA1 region revealed a significant increase in the immunoreactivity of GFAP and Iba-1 in the hippocampal CA1 region of the ChR2-eYFP-expressing mice (Fig 6B). An increased intensity of GFAP immunoreactivity in the hippocampal CA1 region after the optogenetic stimulation was accompanied by morphological changes in astrocytes, reminiscent of reactive astrocytes. The repeated optogenetic stimulation increased the length, thickness, and number of branch in hippocampal astrocytes (Fig 6B). Additionally, we observed an increased number of primary processes leaving the soma in astrocytes from the ChR2-eYFP group compared to the eYFP group. Astrocytes in the ChR2-eYFP group exhibited a bushy morphology with many fine terminal processes protruding from the primary cellular processes, and the processes appeared thicker than those in the eYFP group (Fig 6B). Microglia also showed morphological changes from resting to activated states following the optogenetic stimulation of astrocytes (Fig 6B). We next compared the immunoreactivity of GFAP and Iba-1 in the CA1 versus dentate gyrus (DG) areas on day 3 after repeated optogenetic stimulation. Our data showed increased immunoreactivity of GFAP (S19A Fig) and Iba-1 (S19B Fig) in the hippocampal CA1 area, but not in the DG area. As expected, ChR2-eYFP expression was localized to the hippocampal CA1 area. The activation of astrocytes and microglia has been widely acknowledged to contribute to cognitive impairment through the release of proinflammatory mediators, including IL-1β and TNF-α [27,86–88]. To determine whether the optogenetic stimulation of the astrocytes influenced the expression of proinflammatory genes, the hippocampus was harvested from the eYFP or ChR2-eYFP-expressing mice on day 3 after photostimulation. We detected a significantly enhanced expression of *Il1b* and *Tnf* mRNA in the hippocampus of ChR2-eYFP-expressing mice compared with that of the eYFP-expressing control mice (Fig 6C). These results indicate that the repeated optogenetic stimulation of CA1 astrocytes induces neuroinflammation and reactive phenotype of astrocytes in the hippocampus.

### *Lcn2* deficiency ameliorates neuroinflammation and cognitive impairment after the optogenetic stimulation of hippocampal astrocytes

Studies have demonstrated that LCN2 regulates the immune and inflammatory responses in a range of neurological diseases [38,40]. We have previously reported that LCN2 caused glial activation and increases the expression of inflammatory cytokines [38]. In the present study, we determined whether LCN2 was involved in neuroinflammation and cognitive decline after the repeated optogenetic stimulation of astrocytes, for which we first evaluated the hippocampal expression of LCN2 after optogenetic stimulation. Photostimulation for 20 min for 3 consecutive days resulted in a significant increase in the mRNA expression of *Lcn2* in the hippocampus (S18C Fig). We also investigated whether *Lcn2* deficiency affected glial activation and the subsequent production of proinflammatory cytokines in the hippocampus after photostimulation. As depicted in Fig 6B, the optogenetic stimulation-induced activation of hippocampal astrocytes and microglia was diminished in *Lcn2*-KO mice. In further morphological analysis of astrocytes, *Lcn2* deficiency attenuated the optogenetically enhanced length, thickness, and number of astrocytic branches (primary, intermediate, and terminal processes) (Fig 6B). IMARIS-based 3D morphological analysis showed an enlargement in microglial cell somatic volume, alongside a decrease in the number and length of their processes after repeated optogenetic astrocyte stimulation. These effects were attenuated by *Lcn2* deficiency (S20 Fig). In addition, we examined the changes in the *Il1b* and *Tnf* mRNA expression in the hippocampus of *Lcn2*-KO mice. Results of quantitative PCR (qPCR) demonstrated a significant increase in the levels of these proinflammatory cytokines in the hippocampus of wild-type (WT) mice after photostimulation, which was significantly reduced in *Lcn2*-KO mice (Fig 6C). These findings suggest that LCN2 is an important trigger for neuroinflammation in the hippocampus after the repeated optogenetic stimulation of astrocytes.

We compared the photostimulation-induced cognitive impairment between *Lcn2*-deficient and WT mice. We observed that the cognitive behavior impairment induced by photostimulation was significantly ameliorated in the *Lcn2*-KO mice, as demonstrated by the Y-maze (Fig 6D), Barnes maze (Fig 6E), and passive avoidance tests (Fig 6F). These data indicate that LCN2 plays a vital role in the development of cognitive impairment after the optogenetic stimulation of hippocampal astrocytes. There was no significant difference in the basal excitatory synaptic transmission, and short- and long-term synaptic plasticity between the WT and *Lcn2*-KO mice (S21A–S21F Fig). We then examined potential changes in LTP levels in *Lcn2*-KO mice following 20 min of photostimulation. The degree of TBS-induced LTP alteration showed no significant differences between the eYFP and ChR2-eYFP groups in *Lcn2*-KO mice, with or without photostimulation (S21G and S21H Fig).

### Sustained Gq signaling activation of hippocampal CA1 astrocytes mimics the effect of optogenetic stimulation: LCN2 release, neuroinflammation, and cognitive deficit

**Fig 7:**
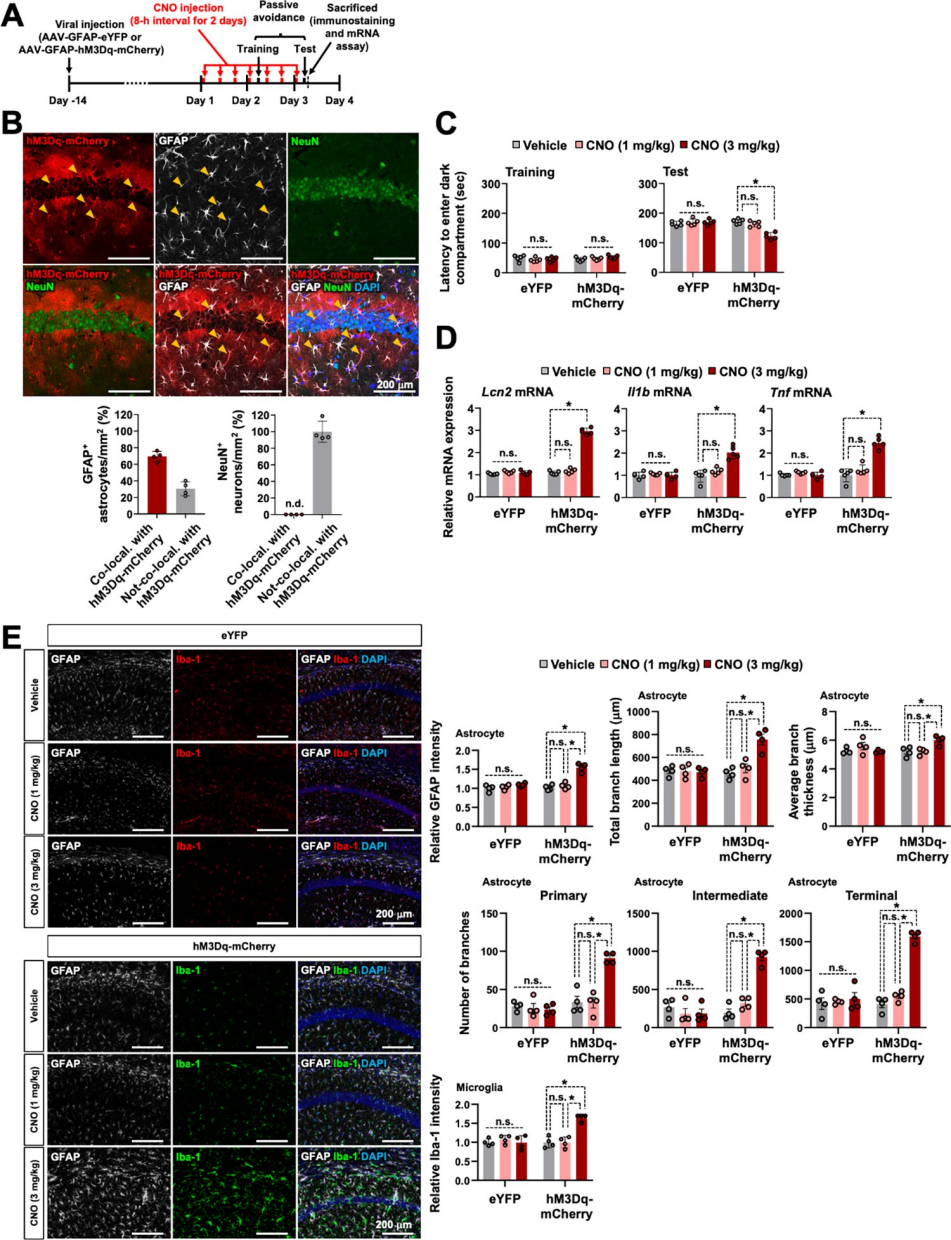
Long-term activation of astrocytic hM3Dq in the hippocampal CA1 region induces LCN2 release, neuroinflammation, and cognitive impairments. (**A**) Experimental timeline. (**B**) Brain tissue samples were subjected to immunofluorescence analysis to localize the expression of hM3Dq-mCherry (red) in the astrocytes (GFAP, white) and neurons (NeuN, green). The nuclei were stained with DAPI (blue). Arrowheads (yellow) indicate the colocalization of hM3Dq-mCherry and GFAP. The quantification of the colocalization is shown in the adjacent graphs. Scale bar: 200 μm. Results are expressed as mean ± SEM (*n* = 4). n.d., not detected. (**C**) Passive avoidance test. Results are expressed as mean ± SEM (*n* = 5). *p < 0.05 between the indicated groups; n.s., not significant (two-way ANOVA). (**D**) On day 3 after CNO (1 or 3 mg/kg, i.p.) injection, total mRNA was extracted from the hippocampal tissues of each group and subjected to qPCR to determine the expression levels of *Lcn2*, *Il1b*, and *Tnf*. *Gapdh* was used as an internal control. The graph displays the quantitative results normalized to *Gapdh*; results are expressed as mean ± SEM (*n* = 5). *p < 0.05 between the indicated groups; n.s., not significant (one-way ANOVA). (**E**) Astrocytes and microglia were identified using GFAP (white) and Iba-1 (eYFP, red; hM3Dq-mCherry, green) immunolabeling after CNO (1 or 3 mg/kg, i.p.) injection. The adjacent graph displays the quantification of the fluorescence intensity (GFAP or Iba-1). In further morphological analysis, the total length and average thickness of astrocyte processes in each group were assessed. Moreover, the total number of primary, intermediate, and terminal branches for each astrocyte was measured. Scale bar: 200 μm. Results are expressed as mean ± SEM (*n* = 4). *p < 0.05 between the indicated groups; n.s., not significant (one-way ANOVA). Source data can be found in S1 Data. CNO, clozapine-N-oxide; LCN2, lipocalin-2.

We used chemogenetic tools to examine whether chronic Gq signaling activation of hippocampal astrocytes mimics the effect of repeated optogenetic stimulation. The muscarinic receptor variant hM3Dq fused to a red fluorescent protein, mCherry, was expressed in the astrocytes within the hippocampal CA1 region using an AAV vector incorporating a GFAP promoter (AAV-GFAP-hM3Dq-mCherry, Fig 7A). To confirm the astrocyte-targeted expression of the hM3Dq-mCherry protein, we performed immunostaining of the hippocampal sections using anti-GFAP antibody and observed that the cells expressing the hM3Dq-mCherry protein were mostly GFAP^+^ astrocytes (Fig 7B). To verify whether hM3Dq activates astrocytes after treatment with clozapine-N-oxide (CNO), we performed Ca^2+^ imaging in cultured astrocytes expressing hM3Dq. For this purpose, cultured astrocytes were loaded with Fluo 4-AM and treated with CNO (10 μm) (S22A Fig). We observed that CNO treatment triggered an intracellular Ca^2+^ increase in hM3Dq-expressing astrocytes (S22B Fig).

The passive avoidance test was performed on the animals to evaluate the ability of chronic Gq signaling activation of hippocampal CA1 astrocytes to regulate cognitive behavior. hM3Dq-mCherry-expressing mice received CNO (1 or 3 mg/kg, intraperitoneal injection) 3 times a day (8 h interval) for 3 days. After the fourth CNO injection (3 mg/kg), the latency to enter the dark compartment was significantly lower in CNO (3 mg/kg)-injected hM3Dq-expressing mice than in control animals in the passive avoidance test trials performed 24 h after the foot shock, thus suggesting impaired memory due to the chemogenetic stimulation of hippocampal astrocytes (Fig 7C). The hM3Dq-expressing mice exhibited no difference in latency to enter the dark compartment during the conditioning session. No apparent changes in cognitive function were detected in eYFP-expressing control mice. These results indicate that the long-term activation of astrocytic Gq signaling resulted in cognitive function impairment.

We explored whether chronic stimulation of astrocytes through hM3Dq triggered LCN2 expression and neuroinflammation. Results from the qPCR analysis revealed the enhanced mRNA expression of *Lcn2* and proinflammatory cytokines (*Il1b* and *Tnf*) in the hippocampus after chemogenetic stimulation (Fig 7D). Moreover, chemogenetic stimulation induced the activation of both microglia and astrocytes (Fig 7E). In detailed morphological analysis, we observed an increased number of primary processes leaving the soma in astrocytes from the hM3Dq-mCherry group compared to the eYFP group (Fig 7E). Astrocytes in the hM3Dq-mCherry group exhibited a bushy morphology with many fine terminal processes protruding from the primary cellular processes, and the processes appeared thicker than those in the eYFP group (Fig 7E). These effects were only observed after treatment with a high concentration of CNO (3 mg/kg), but not when the concentration was low (1 mg/kg). Overall, these data suggest that the long-term and strong Gq signaling activation of CA1 astrocytes significantly impairs cognitive behavior and increases glial activation and expression of *Lcn2* and proinflammatory cytokines.

### Pharmacological inhibition of astrocytes reduces the release of LCN2, neuroinflammation, and cognitive deficit

**Fig 8:**
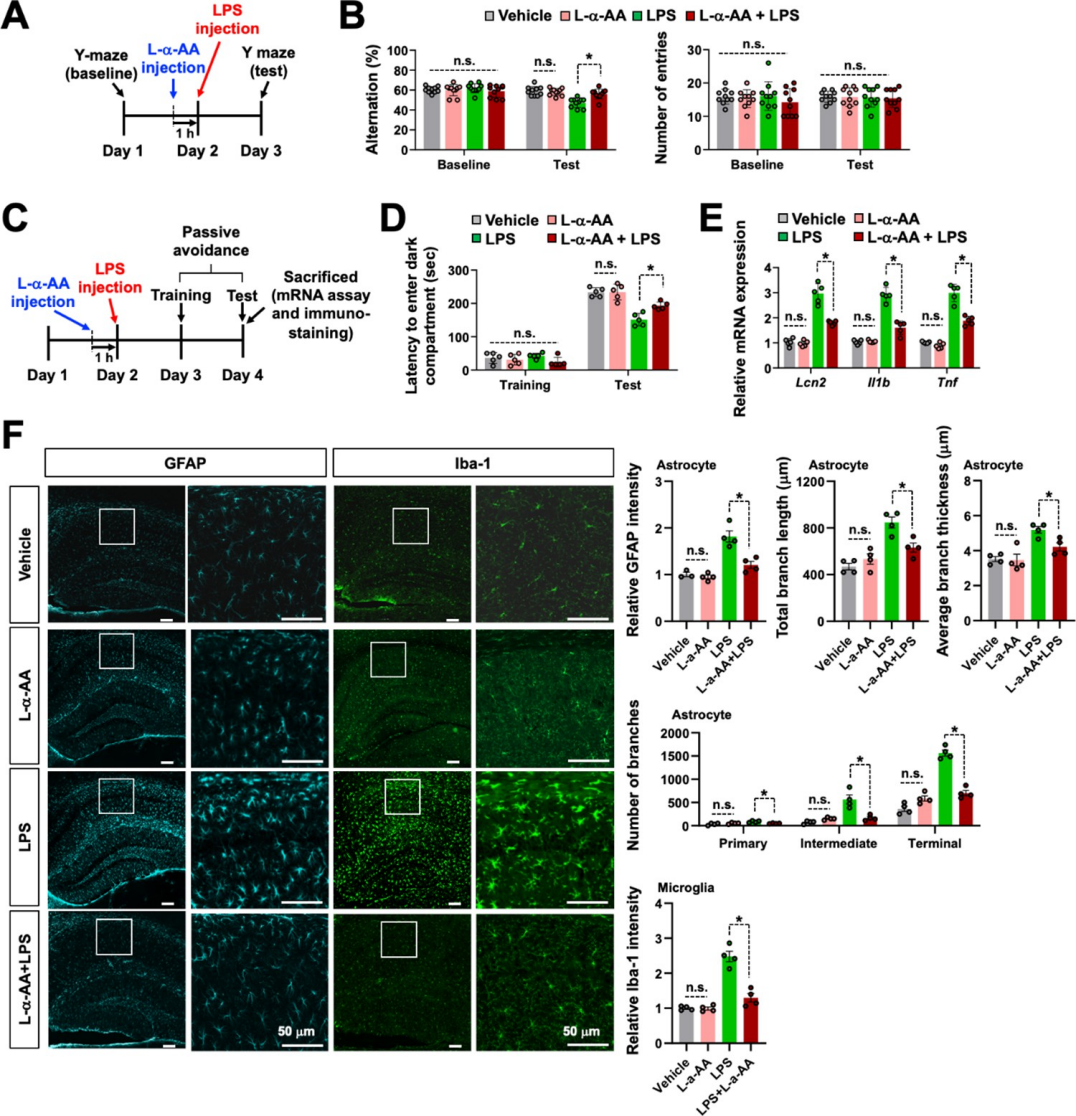
Astrocyte toxin blocks neuroinflammation-associated cognitive impairment. **(A)** Experimental timeline of l-α-AA (10 nM, i.c.v.) and LPS (2 μg, i.c.v.) injection to the hippocampal CA1 region for the Y-maze test. (**B**) Performance in the Y-maze test. Results are expressed as mean ± SEM (*n* = 10). *p < 0.05 between the indicated groups; n.s., not significant (one-way ANOVA). (**C**) Experimental timeline of l-α-AA and LPS injection for the passive avoidance test. (**D**) Performance in the passive avoidance test. Results are expressed as mean ± SEM (*n* = 5). *p < 0.05 between the indicated groups; n.s., not significant (one-way ANOVA). (**E**) At 48 h after LPS injection, total mRNA was extracted from the hippocampal tissues of each group and subjected to qPCR to evaluate the expression levels of *Lcn2*, *Il1b*, and *Tnf*. *Gapdh* was used as an internal control. The graph displays the quantitative results normalized to *Gapdh*; results are expressed as mean ± SEM (*n* = 5). *p < 0.05 between the indicated groups; n.s., not significant (one-way ANOVA). (**F**) Hippocampal CA1 sections prepared from the vehicle- or l-α-AA-injected mice at 48 h after LPS injection were stained with anti-GFAP (cyan) and anti-Iba-1 (green) antibodies. The graph displays the quantification of the fluorescence intensity. Scale bar: 50 μm. In further morphological analysis, the total length and average thickness of astrocyte processes in each group were assessed. Moreover, the total number of primary, intermediate, and terminal branches for each astrocyte was measured. Results are expressed as mean ± SEM (*n* = 3 or 4). *p < 0.05 versus indicated groups; n.s., not significant (one-way ANOVA). Source data can be found in S1 Data. LPS, lipopolysaccharide.

**Fig 9:**
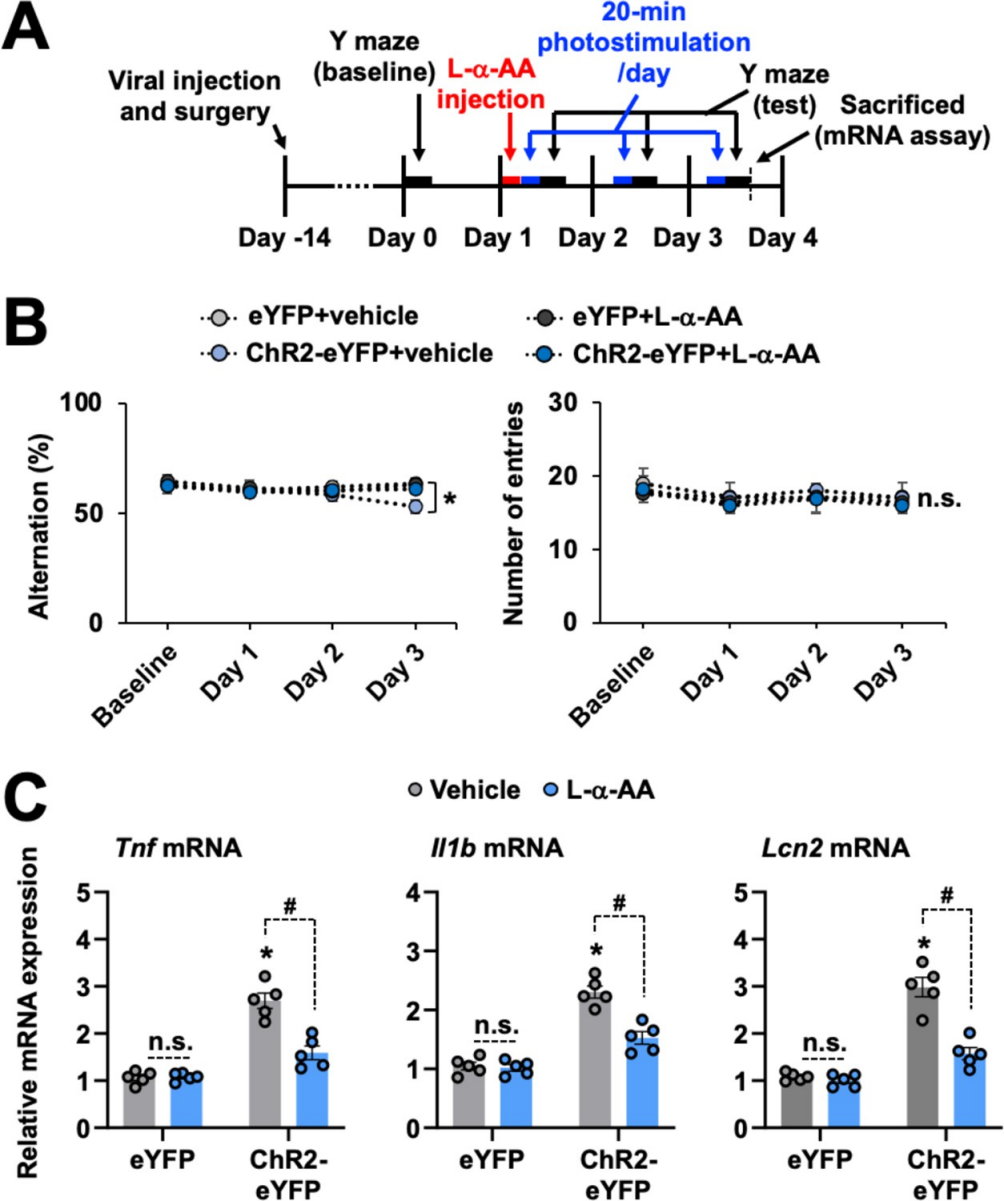
Astrocyte toxin attenuates the repeated 20-min photostimulation-induced cognitive impairment and neuroinflammation. **(A)** Experimental timeline of l-α-AA (10 nM) intracerebroventricular injection to the hippocampal CA1 region for the Y-maze test. (**B**) Performance in the Y-maze test. Results are expressed as the mean ± SEM (*n* = 5). *p < 0.05 between the indicated groups; n.s., not significant (one-way ANOVA). (**C**) Total mRNA was extracted from the hippocampal tissue of each group and subjected to qPCR to evaluate the expression levels of *Tnf*, *Il1b*, and *Lcn2*. *Gapdh* was used as an internal control. The graph displays the quantitative results normalized to *Gapdh*; results are expressed as mean ± SEM (*n* = 5). *p < 0.05 versus eYFP (vehicle) groups; #*p* < 0.05 between the indicated groups; n.s., not significant (one-way ANOVA). Source data can be found in S1 Data. LCN2, lipocalin-2.

Our results obtained strongly indicate that the sustained stimulation of hippocampal astrocytes was sufficient for cognitive decline. To determine whether hippocampal astrocyte activation is required to induce cognitive decline, we used a metabolic astrocyte inhibitor, l-α-AA, and a lipopolysaccharide (LPS)-induced neuroinflammation model. l-α-AA is known to selectively inhibit astrocyte metabolism and alleviate nerve injury and inflammatory pain [89–95]. Results of the Y-maze (Fig 8A and 8B) and passive avoidance (Fig 8C and 8D) tests indicated that intracerebroventricular injection of l-α-AA (10 nM) significantly attenuated the LPS injection (2 μg)-induced cognitive decline. Similarly, the LPS-induced mRNA expression of *Lcn2*, *Il1b*, and *Tnf* (Fig 8E) and glial activation (Fig 8F) in the hippocampus were suppressed by l-α-AA administration. Furthermore, we observed an increased number of primary processes leaving the soma in astrocytes in LPS-injected group, which was attenuated by l-α-AA administration (Fig 8F). Similar findings were observed in the length and thickness of astrocytic branches (Fig 8F). These results indicate the crucial role of astrocyte activation in LPS-induced neuroinflammation and cognitive impairment. Similarly, treatment with l-α-AA reduced the optogenetic stimulation-induced cognitive impairments (Fig 9A and 9B) as well as the *Lcn2* and cytokine mRNA expression levels (Fig 9C) in the ChR2-eYFP group. Combined with the data obtained from the optogenetic and chemogenetic stimulation experiments, this inhibitor study suggests that hippocampal CA1 astrocyte activation is sufficient and necessary for LCN2 release, neuroinflammation, and subsequent cognitive decline.

### Hippocampal astrocytes are activated in the neuroinflammation model as determined by Ca^2+^ fiber photometry

Based on the finding that optogenetic or chemogenetic stimulation of hippocampal CA1 astrocytes promotes neuroinflammation and cognitive dysfunction, we investigated whether the activity of the hippocampal CA1 astrocytes changed under neuroinflammatory conditions. For this purpose, we directly measured the Ca^2+^ dynamics of the hippocampal CA1 astrocytes in living animals using a fiber photometry technique. GCaMP6f, a Ca^2+^-sensing fluorescent protein, was selectively expressed in hippocampal CA1 astrocytes through viral vectors (AAV-GFAP-GCaMP6f). Immediately after LPS injection, the amplitude of the Ca^2+^ events of astrocytes was significantly elevated compared with the phosphate buffered saline (PBS)-injected controls (S23A and S23B Fig). The Ca^2+^ dynamics (i.e., the number, duration, and amplitude of Ca^2+^ transients) was further analyzed at 0, 3, and 24 h in the hippocampal CA1 region of the LPS-induced neuroinflammation model (S23C Fig). We detected a significantly increased number of spontaneous Ca^2+^ transients in the CA1 area at 24 h after LPS injection, which suggests that the astrocytes were activated in the inflamed hippocampus (S23C Fig). However, there were no differences in the Ca^2+^ frequency at 0 and 3 h after LPS injection compared with the baseline. To confirm the astrocyte-targeted expression of the GCaMP6f, we performed immunostaining of the hippocampal sections using anti-GFAP antibody and observed that the cells expressing the GCaMP6f were mostly GFAP^+^ astrocytes (S23D Fig). These results indicate that astrocytes in the hippocampus are hyperactivated under inflammatory conditions.

## Discussion

**Fig 10:**
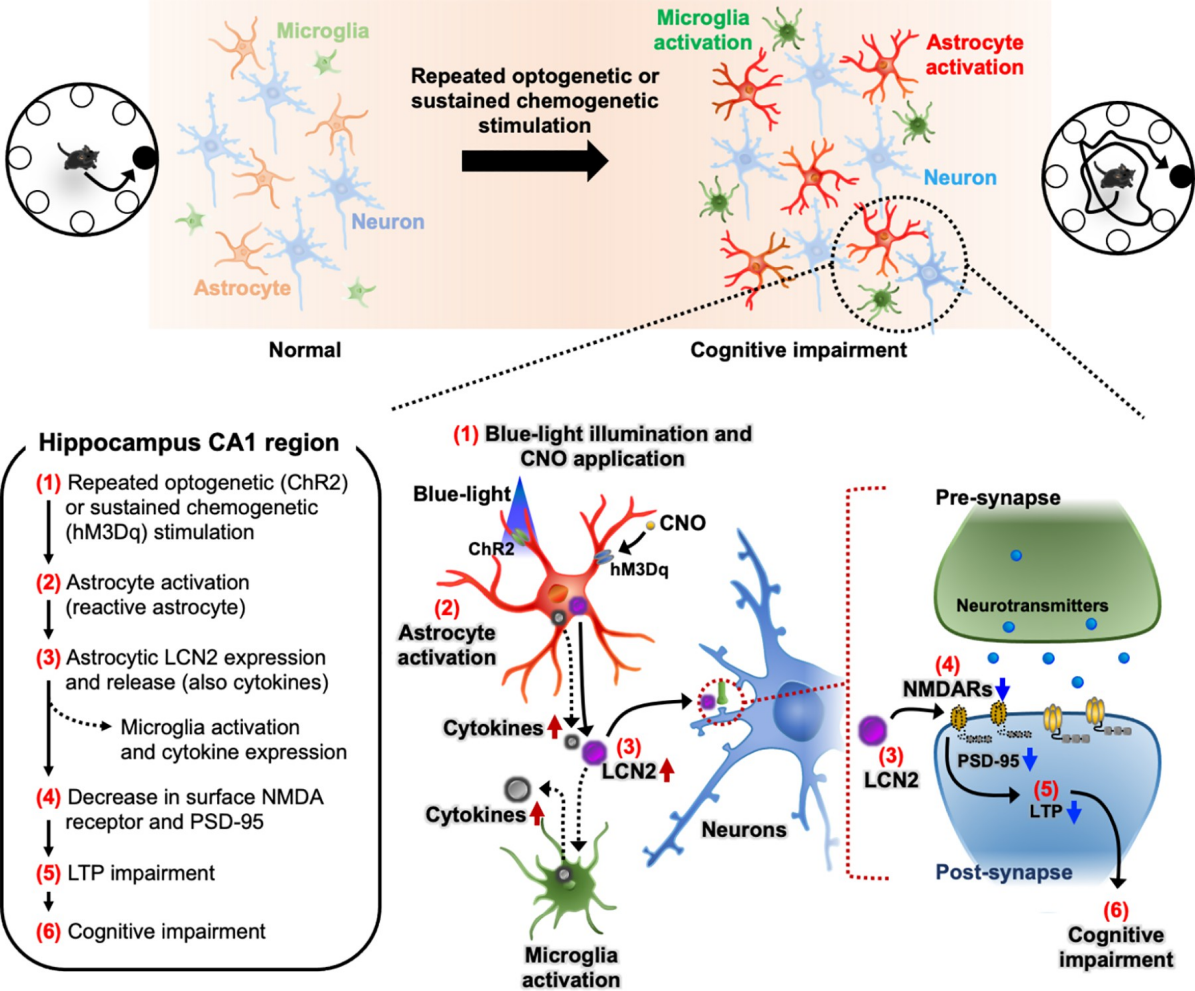
Schematic diagram depicting the proposed mechanism of cognitive dysfunction after optogenetic or chemogenetic stimulation of hippocampal astrocytes. Repeated optogenetic or sustained chemogenetic stimulation of astrocytes in the hippocampus induces cognitive impairment in mice. LCN2 derived from reactive astrocytes in the inflamed hippocampus mediates the cognitive deficit through a series of events, (1)–(6), in the figure. LCN2, lipocalin-2.

In this study, we demonstrated the process of hippocampal astrocyte-mediated cognitive impairment, as summarized in Fig 10. Repeated and sustained stimulation of hippocampal CA1 astrocytes using optogenetic and chemogenetic tools induced neuroinflammation and reactive astrocyte phenotype. The resultant LCN2 released from the reactive astrocytes decreased the NMDA receptor-mediated synaptic activity, thereby affecting the long-term synaptic plasticity of hippocampal CA1 neurons and resulted in cognitive decline. Our optogenetic and chemogenetic gain-of-function studies were complemented by a loss-of-function study conducted using an astrocyte metabolic inhibitor, which together demonstrated that hippocampal astrocyte activation is sufficient and necessary for cognitive decline. Finally, our conclusion was further supported by the in vivo detection of astrocyte activation in the hippocampus under neuroinflammatory conditions using Ca^2+^ fiber photometry.

We demonstrated that directly increasing the activation of the hippocampal CA1 astrocytes using optogenetic and chemogenetic tools for a sustained period of time dramatically impaired cognitive function. The condition we applied in the optogenetic and chemogenetic approaches may mimic a pathological state, wherein the astrocytes are aberrantly activated to deregulate cognitive function. During inflammatory and pathological conditions, LCN2 emerges as a critical factor, acting as a chemokine inducer and neuronal cell death mediator [96–98]. LCN2 induction is observed in various neurological conditions, including multiple sclerosis, chronic inflammatory pain, neuropathic pain, Alzheimer’s disease, Parkinson’s disease, vascular dementia, ischemic stroke, intracerebral hemorrhage, stab wound injury, and traumatic brain injury [38,99]. This suggests that changes in LCN2 levels may be linked to various brain injuries and disorders. LCN2 functions as a neurotoxin and marker protein, reflecting or influencing the state and phenotype of activated astrocytes [100–103]. These findings suggest that targeting specific brain regions may be employed to model diseases where reactive astrogliosis and subsequent LCN2 release and neuroinflammation may be induced by repeated optogenetic or sustained chemogenetic stimulation. However, further research is warranted to elucidate how reactive astrogliosis differs among diseases, including aspects such as astrocyte heterogeneity and metabolism [32,104,105].

The optogenetic stimulation paradigm for astrocytes may be different from that of neurons. A short stimulation might be sufficient for fast neuronal excitation; however, in the literature relatively long-term stimulation has been frequently used for astrocytes, which display slower responses than neurons in the various brain regions and behaviors [106–109]. Repeated optogenetic stimulation of astrocytes over several days has also been reported in the context of anxiety and spinocerebellar ataxia, etc. In the chronic activation of astrocytes, light was delivered for 10 min every 2 days for a total of 21 days of light stimulation [110] and 4 days of blue light stimulation for chronic optogenetic stimulation of Bergman glia [63]. In the current manuscript, the optogenetic stimulation paradigm, i.e., once-a-day optogenetic stimulation of astrocytes over 3 days, has been selected to induce an inflammatory condition in the hippocampus. Typically, it requires a few days of stimulation for neuroinflammation to peak in mouse models, such as LPS-induced neuroinflammation [111–115] and ATP-induced neuroinflammation [116]. Recent studies show conflicting effects of astrocyte stimulation on memory processes: Some findings suggest an enhanced memory formation through astrocyte stimulation [15,74], while others suggest impaired memory function [107,117] (S1 Table). These outcome variations may arise from the diverse substances released by astrocytes in response to varied stimulation patterns, differentially affecting synaptic transmission. Our study demonstrates impaired memory function following repeated optogenetic astrocyte stimulation via ChR2. This contrasts with the enhanced neuronal activity observed with Optoα1AR stimulation in a previous study [118]. Additionally, our study reveals reactive astrocyte induction via repeated optogenetic and chemogenetic stimulations—a phenomenon not observed with Optoα1AR stimulation [74]. Previous research on the underlying mechanisms of varying outcomes reveals that the source of Ca^2+^ influx—internal stores versus extracellular space—may influence the substances released by astrocytes, subsequently affecting synaptic modulation [119,120]. Furthermore, recent studies utilizing chemogenetic methods have demonstrated that astrocytic stimulation via Gq-GPCR (hM3Dq) induces long-term potentiation and enhances memory [15]. Conversely, astrocytic stimulation via Gi-GPCR (hM4Di) impairs remote memory recall and disrupts CA3 to CA1 communication [117]. Additionally, our previous study demonstrated that stimulating astrocytes via Gi-GPCR (hM4Di) reduces neuroinflammation and ameliorates memory impairment in an LPS-induced neuroinflammation model [121]. These findings underscore the significance of stimulation protocols in astrocyte manipulation using optogenetics and chemogenetic tools. Recently, extensive reviews abound regarding the role of astrocytes in memory formation and maintenance [122].

It is possible that the photostimulation of ChR2-expressing astrocytes used in the present study differs from the physiological stimuli that activate astrocytes within the brain. Nonetheless, optogenetic manipulation of astrocytes has been successfully used to study various behavioral responses [123,124]. Our previous study also showed that photostimulation evokes an increase in the intracellular Ca^2+^ concentration as well as its sustained oscillation [106]. Thus, our experimental method of using ChR2 (Ad-ChR2-Kat1.3 or AAV-ChR2-eYFP) appears to drive the Ca^2+^ responses in ChR2-expressing astrocytes. These Ca^2+^ responses were elicited by the activation of G-protein coupled receptors in the native astrocytes [125,126] and successfully triggered the release of gliotransmitters [127,128]. While astrocyte intracellular Ca^2+^ signaling is critical for astrocyte-neuron communication, the functional relevance of different features of Ca^2+^ signaling remains a topic of exploration [129,130]. Astrocytes also undergo morphological, molecular, and functional remodeling in response to injury, disease, or infection of the CNS, which has been extensively reviewed by Escartin and colleagues [37]. The pathological context in which astrocyte reactivity occurs can vary dramatically, e.g., sporadic versus genetically mediated, acute versus chronic, and due to a systemic pathology (i.e., sepsis), specific injury or disease of the CNS, or experimental manipulations (such as optogenetic stimulation). Astrocytes may also exhibit cell-autonomous disturbances [131], which can occur in astrocytopathies resulting from mutated alleles of astrocytic genes (e.g., GFAP in Alexander disease) [132]. An important implication of the disease-specific induction of the distinct reactive astrocyte states is that the damage- and pathogen-associated stimuli from one condition cannot be assumed to be active in another. Likewise, exposure to Tau, amyloid-β, or α-synuclein needs to be carefully designed to replicate the concentration, protein species, and combinations thereof found in patient brains. Moreover, the outcome of activating a signaling pathway may depend on the upstream stimuli [133] or priming caused by previous exposure to other stimuli [134]. Thus, careful selection of upstream stimuli is essential for appropriate in vivo and in vitro modeling of disease-specific reactive astrocytes. It is not completely understood how or whether changes in Na^+^, K^+^, Cl^−^, and Ca^2+^ fluxes and second messengers triggered by optogenetic approaches modulate the signaling cascades driving the phenotypical changes in reactive astrocytes.

Neuroinflammation has emerged as a critical regulator of many CNS pathologies and is mediated by the complex molecular crosstalk between microglia and astrocytes. Microglia and astrocytes are dynamic glial cells that respond to all CNS insults and undergo context-dependent morphological and functional changes [23]. These glial cells continuously interact to regulate the CNS microenvironment during healthy and disease/injured states [24]. Following insults to the brain tissue, microglia undergo extensive transcriptional reprogramming, releasing a plethora of cytokines and inflammatory mediators. These secreted molecules function as messengers to facilitate the communication between microglia and astrocytes [135]. However, it is also important to consider the reciprocal regulation of microglia by astrocytes [24]. In this regard, various cytokines/chemokines and inflammatory mediators, such as IL-1β, IL-10, IL-15, TNF-α, nitric oxide, and chemokine ligand 2 (CCL2) are also secreted by astrocytes, which act on microglia to regulate their function [24,25,136–139]. We also found an increased expression of *Tnf* and *Il1b* mRNA in the cultured astrocytes after photostimulation (S14C Fig). These results suggest that reactive astrocytes may secrete cytokines to promote the activation of microglia (Fig 10). It would be valuable to explore the role of microglia in astrocyte-mediated changes in cognitive behaviors. A recent study has shown that microglia-derived galectin-3 influences synaptic plasticity and brain rhythmicity, which are implicated in neuroinflammation and cognitive decline [140,141].

LCN2 serves as a key mediator of neuroinflammation and cognitive decline, with astrocytes playing a pivotal role in these processes. Astrocytes, responsible for supporting neurons and maintaining CNS homeostasis, exhibit distinct activation phenotypes—classical and alternative—depending on the context of inflammation. LCN2 drives the classical activation phenotype in both astrocytes and microglia, influencing pro-inflammatory responses and potentially contributing to brain injury. Our study investigated the release of LCN2 from hippocampal CA1 astrocytes during optogenetic stimulation, revealing its involvement in cognitive impairment. Moreover, we demonstrated that LCN2 modulates hippocampal synaptic activity by reducing the surface expression of NMDA receptors, inhibiting synaptic LTP, and influencing the excitability of CA1-CA3 synapses. While the precise molecular mechanisms remain unclear, these findings highlight the intricate role of LCN2 in astrocyte-mediated cognitive decline and suggest potential therapeutic avenues for targeting specific astrocyte activation phenotypes. The involvement of additional astrocyte-derived substances warrants further investigation. Astrocyte-derived adenosine has been demonstrated to modulate spike timing-dependent long-term synaptic plasticity during development [142].

Conventional *Lcn2* KO (whole-body KO) animals were utilized in our study, which comes with limitations in determining the precise role of astrocyte-secreted LCN2. RNA sequencing analysis reveals LCN2 expression in microglia/resident macrophages, endothelial cells, and astrocytes [143]. Consequently, we cannot exclude the potential involvement of LCN2 from microglia/macrophages and endothelial cells. Previous studies have shown that microglia became activated due to neuronal damage or inflammation, resulting in increased LCN2 secretion [144,145]. Additionally, endothelial cell-derived LCN2 contributed to neuronal cell death in ischemic brain injury [58]. To address these limitations, future studies should use conditional knockout or viral tools targeting astrocytes. These approaches allow for precise cell type-specific control of LCN2 expression. Nevertheless, our immunofluorescence analysis revealed that 95.8% of total LCN2-positive cells co-localized with GFAP immunoreactivity in the ChR2-eYFP groups after repeated photostimulation. This suggests that most LCN2-expressing cells were astrocytes in our experimental conditions.

In summary, our findings indicate that the aberrant activation of astrocytes in the hippocampus negatively affects the excitatory synaptic transmission and eventually cognitive performance. Moreover, LCN2 derived from the reactive astrocytes in the inflamed hippocampus may be involved in mediating these synaptic changes under this condition. Therefore, this type of pathological glial–neuron interaction might underlie the cognitive decline observed during neuroinflammatory conditions. These results suggest that targeting the astrocyte pathways and LCN2 represents a novel therapeutic prospect to prevent cognitive alterations in various CNS disorders.

## Materials and methods

### Animals

Male C57BL/6 mice (aged 8 to 12 weeks) were obtained from Samtaco (Osan, South Korea), and *Lcn2*-knockout (KO) C57BL/6 mice (aged 8 to 12 weeks) were kindly provided by Drs. Kiyoshi Mori (Kyoto University, Kyoto, Japan) and Shizuo Akira (Osaka University, Osaka, Japan). Only male mice were used in this study. Animal maintenance and experimental procedures were performed as reviewed and approved by the Institutional Animal Care and Use Committee of Kyungpook National University (Approval No. KNU 2019–0090). Animal care and use protocols were adhered to US National Institutes of Health (NIH) guidelines, Korean national legislation, and institutional requirements.

### Viral gene transfer and optogenetic stimulation in vivo

The following viral constructs were used: AAV5-GFAP-ChR2-eYFP (VVF Zurich; viral titer 3.9 × 10^12^ virus genomes per ml; vg/ml), AAV5-GFAP-hM3Dq-mCherry (VVF Zurich; viral titer 3.9 × 10^12^ vg/ml), AAV5-GFAP-Lck-GCaMP6f (PENN Vector Core; viral titer 6.4 × 10^13^ vg/ml), AAV5-GFAP-eYFP (control vector; VVF Zurich; viral titer 3.9 × 10^12^ vg/ml), and adenoviral vectors (Ad-GFP and Ad-GFAP-ChR2-Katushka1.3; viral titer 1.6 × 10^11^ vg/ml; kindly provided by Dr. Kasparov from the University of Bristol) [146]. To prepare the animals for in vivo experiments, WT and *Lcn2*-KO male mice were anesthetized using 2% to 4% isoflurane (Baxter, Deerfield, Illinois, United States of America) in oxygen and placed in a stereotaxic apparatus. For in vivo optogenetic stimulation, 2 stainless steel guide cannulas were bilaterally implanted, and the tip was gently lowered to 0.5 mm above the hippocampus (from the bregma by 2 mm posterior, 1.8 mm lateral, and 1.2 mm dorsoventral) to prevent damage to the target region. All the guide cannulas were secured in place using dental cement. A volume of 0.5 μl of the virus was injected bilaterally at a rate of 0.1 μl/min. After injection, the needle tip was held in place for 10 min before retraction to prevent leakage and then removed. Subsequently, an optical fiber (200 μm in diameter; 0.37 NA; the maximal light intensity at the tip of the optical fiber, 1.7 mW) was inserted into guide cannula, so that the tip of the optical fiber was placed 0.2 mm above the hippocampal CA1 region, before being fixed using dental cement. Immediate postoperative care was provided, and the animals were allowed to recover for 14 days before the experiments to ensure a high level of transgene expression. In the behavioral experiments following viral gene transfer, the expression of the relevant proteins within the dorsal hippocampus CA1 region was confirmed as fluorescence signals. For the behavior, electrophysiology, and microdialysis experiments, optogenetic stimulation was delivered through the optical fiber for 5-, 10-, or 20-min a day using DPSS 473 nm blue lasers (500 ms light on, 500 ms light off cycle, 1 Hz) (Shanghai Dream Laser Technology) to the eYFP or ChR2-eYFP-expressing astrocytes.

### Immunofluorescence staining

The immunofluorescence staining of brain tissues was performed as previously described [147]. For the double or triple immunofluorescence analysis, tissue sections were incubated with mouse anti-GFAP (BD Biosciences, San Diego, California, USA, Catalog number: 556330), rabbit anti-GFAP (Dako, Glostrup, Denmark, Catalog number: Z0334), rabbit anti-Iba-1 (Wako, Osaka, Japan, Catalog number: 019–19741), mouse anti-NeuN (Millipore, Burlington, Massachusetts, USA, Catalog number: MAB377), goat anti-LCN2 (Santa Cruz Biotech, Santa Cruz, California, USA, Catalog number: sc-23430), and mouse anti-βIII-tubulin (Santa Cruz, Catalog number: sc-80005) antibodies. Sections were visualized by incubation with Cy3-, Cy5-, and FITC-conjugated anti-mouse, rabbit, or goat IgG antibody (The Jackson Laboratory, Bar Harbor, Maine, USA; Cy3-mouse; Catalog number: 715-165-151, Cy3-rabbit; Catalog number: 711-165-152, Cy3-goat; Catalog number: 705-165-147, Cy5-rabbit; Catalog number: 711-175-152, FITC-mouse; Catalog number: 715-095-151, FITC-rabbit; Catalog number: 711-096-152, FITC-goat; Catalog number: 705-095-147) and examined under a fluorescence or confocal microscope. Fluorescence intensities were quantified using ImageJ software version 1.44 (National Institutes of Health, Bethesda, Maryland, USA).

The 2D analysis of microglia morphology was conducted as previously described [148]. In brief, confocal image stacks were collected using a 4× objective lens with a 0.5-μm interval through a 10-μm z-depth of the tissue. The image stacks were processed by the maximum intensity projection to create 2D images. The 2D images were imported into the Zen blue lite 2.3 program (Carl Zeiss, Oberkochen, Germany). Cell body sizes were measured using “Draw Spline Contour” in the “Graphics” section. Microglia were divided into 2 groups: ramified cells were regarded as resting microglia, while roundish cells with fewer branches were considered activated. A cell-body diameter of 10 μm was set as the cut-off criterion. Microglia possessing smaller cell bodies with long, lean, and relatively more branches were considered to be resting microglia. In contrast, microglia with a cell body larger than 10 μm with thick and hardly any branches were regarded as activated.

For 3D analysis of microglial cells, including somatic volume, process number, and total processes length, confocal image stacks were acquired using a 20× objective lens at 0.5-μm intervals across a z-depth of the tissue of 15 μm. The image stacks were processed with maximum intensity projection to generate 3D images. The 3D images were imported into the LAS X software (Leica Microsystems, Wetzlar, Germany) and subsequently analyzed using IMARIS software (Version 9.5.1, Bitplane, Zurich, Switzerland). First, IMARIS was utilized to reconstruct the microglia surface, with subsequent application of the filter function to eliminate nonspecific background signals. The surface reconstruction then served as the template for filament reconstruction, employing the following custom settings: detection of new starting points, the largest diameter of 9.00 μm, and seed points of 2.00 μm; removal of seed points around the starting points, with a sphere region diameter of 15 μm. Seed points were manually adjusted—either placed within or removed from the somata center—if the IMARIS algorithm misallocated them [149–151]. All surface and filament parameters were exported to separate Excel files for data analysis. Image processing, 3D reconstruction, and data analysis were conducted in a blinded manner to the experimental conditions.

Analysis of astrocyte morphology was conducted as previously described [152–155]. Confocal images were preprocessed using an automated macro in Fiji-ImageJ software. For each cell, maximum projection images were converted into binary images. Thresholds were set to label the cell body and processes, avoiding background pixels. To determine the length and thicknesses of processes, the complexity of astrocyte processes from different regions was assessed. The analysis was carried out using the AutoSholl Analysis plugin of ImageJ [155].

### Behavioral testing

#### Y-maze test

The Y-maze test was conducted as previously described with slight modifications [156]. Spatial cognition was evaluated using the spontaneous alternation task in the Y-maze apparatus. Animals were initially placed within the center, and the sequence (i.e., ABCCAB) and number of arm entries were recorded manually for each animal over a 7-min period. A spontaneous alternation was defined as entries into all 3 arms on consecutive choices (i.e., ABC, CAB, or BCA, but not BAB). The percentage of alternations was defined using the following equation: % alternation = [(number of alternations) / (total arm entries)] × 100. The number of total arm entries was used as an indicator of locomotor activity.

#### Passive avoidance test

This test began with training in which a mouse was placed in a light compartment; when the mouse crossed over to the dark compartment, it received mild (0.25 mA/1 s) electric shock on the foot. This initial latency to enter the dark (shock) compartment was used as the baseline measure. During the probe trials, 1 or 24 h after training, the mouse was again placed in the light compartment, and the latency to return to the dark compartment was measured as an index of passive fear avoidance.

#### Barnes maze test

The Barnes maze test was conducted as previously described with slight modifications [157]. There are 19 imitation holes circled around the maze to distract from the real hole. The imitation holes resemble the escape box but do not lead to an escape chamber. In the habituation phase, the mice were allowed to freely explore the arena for 20 min. Then, they were then moved to the escape box through the cylinder. After they had spent 5 min in the escape box, they were returned to the home cage. This procedure was performed once per mouse. In the training phase, the mice explored the arena until they entered the escape box. Once the experimenter confirmed that the mouse had successfully entered the escape box, the recording was turned off. In the probe test, the mice explored the arena without the escape box. This trial was conducted once per mouse.

### Electrophysiology

Mice (male WT and *Lcn2*-KO mice, aged 8 to 12 weeks) were decapitated under ketamine anesthesia (50 mg/kg, intraperitoneal injection). The brain was quickly removed and immersed in ice-cold Ringer solution containing 119 mM NaCl, 2.5 mM KCl, 1.3 mM MgSO_4_, 2.5 mM CaCl_2_, 1.0 mM NaH_2_PO_4_, 26.2 mM NaHCO_3_, and 11 mM glucose that had been saturated with 95% O_2_ and 5% CO_2_. The hippocampi were dissected and transverse slices (400-μm thick) were prepared using a microslicer (VT1000S; Leica, Nussloch, Germany). The slices were placed in a humidified holding chamber for at least 1 h and then transferred to a recording chamber that was continuously superfused with a bath solution (120 mM NaCl, 2 mM KCl, 1 mM KH_2_PO_4_, 26 mM NaHCO_3_, 2 mM CaCl_2_, 1 mM MgCl_2_, and 10 mM glucose, saturated with 95% O_2_ and 5% CO_2_) at a rate of approximately 2 ml/min.

Field EPSPs were recorded at the stratum radiatum of the CA1 region using an Axopatch-1D amplifier (Molecular Devices; Union City, California, USA). For field recording, a glass microelectrode filled with 2 M NaCl was used, and the bath solution routinely contained 100 μm picrotoxin, a γ-aminobutyric acid type A (GABA_A_) receptor antagonist. Glutamatergic excitatory postsynaptic currents (EPSCs) were recorded from the CA1 neurons using a computer-controlled patch clamp amplifier (MultiClamp 700B; Molecular Devices). For whole-cell recording, patch pipettes were prepared from borosilicate capillary glass (1.5 mm outer diameter, 0.9 mm inner diameter; G-1.5; Narishige, Tokyo, Japan) using a pipette puller (P-97; Sutter Instrument Co., Novato, California, USA). The resistance of the recording pipettes filled with an internal solution (140 mM CsMeHSO_3_, 10 mM CsCl, 2 mM EGTA, 2 mM Mg-ATP, and 10 mM HEPES (pH 7.2), with Tris-base) was 4 to 6 MΩ. In the whole-cell recordings, 10 mV hyperpolarizing step pulses (30 ms in duration) were periodically delivered to monitor the access resistance (15 to 20 MΩ), and recordings were discontinued when the access resistance changed by >15%. The signal was filtered at 3 kHz, digitized at 10 kHz, and stored on a personal computer equipped with pCLAMP 10.7 (Molecular Devices). Electrical stimuli were delivered at 0.1 Hz through a bipolar tungsten electrode using a stimulator (SEN-7203, Nihon Kohden, Tokyo, Japan) equipped with an isolator unit (SS-701J, Nihon Kohden), and the LTP was induced by TBS protocols as follows: bursts of 4 pulses at 100 Hz, with the bursts repeated in 10 trains at 5 Hz, and the trains were repeated 4 times delivered by 10 s. LTD was induced by PP-LFS protocols [158] as follows: paired stimulation with an interval of 50 ms, 1 Hz for 15 min. In the experiments examining the NMDA/AMPA ratio, AMPA receptor-mediated EPSCs were recorded at a holding potential of −60 mV in the presence of 10 μm SR95531 and 50 μm D-APV, selective GABA_A_ and NMDA receptor antagonists, respectively. The NMDA receptor-mediated EPSCs were recorded at a holding potential of +20 mV in the presence of 10 μm SR95531 and 20 μm CNQX, a selective AMPA/kainate receptor antagonist. All electrophysiological experiments were conducted at room temperature (22 to 25°C).

The amplitudes of EPSCs elicited by electrical stimulation were calculated by subtracting the baseline value from the peak amplitude. The extent of LTP was determined by the % increase in the mean fEPSP slope 50 to 60 min after TBS of the baseline (the mean fEPSP slope 10 min before the LTP induction). The extent of LTD was determined by the % decrease in the mean fEPSP slope 35 to 40 min after PP-LFS of the baseline. Spontaneous mEPSCs were counted and analyzed using the MiniAnalysis program (Synaptosoft, Inc., Decatur, Georgia, USA). Briefly, mEPSCs were automatically screened using an amplitude threshold of 10 pA and then visually accepted or rejected based on the increase and decay times. Basal noise levels during voltage-clamp recordings were <10 pA. The average values of both the frequency and amplitude of mEPSCs were calculated for each recording (10 min).

### Western blot analysis

An equal amount of protein for each sample was resolved by SDS-PAGE, transferred to polyvinylidene difluoride membranes (PVDF; Bio-Rad, Hercules, California, USA), and probed with primary antibodies against rabbit anti-phospho-NR1 (p-NR1) (Millipore, Burlington, Massachusetts, Catalog number: ABN88), mouse anti-NR1 (Abcam, Waltham, Massachusetts, Catalog number: ab134308), rabbit anti-NR2A (Invitrogen, Waltham, Massachusetts, Catalog number: 480031), rabbit anti-NR2B (Sigma-Aldrich, Catalog number: 06–600), mouse anti-PSD-95 (Invitrogen, Waltham, Massachusetts, Catalog number: MA1-046), mouse anti-GLUR1 (Millipore, Burlington, Massachusetts, Catalog number: MAB2263), mouse anti-GLUR2 (Millipore, Burlington, Massachusetts, Catalog number: MABN1189), rabbit anti-VGLUT2 (Synaptic Systems, Gottingen, Germany, Catalog number: 135 418), rabbit anti-Homer (Synaptic Systems, Gottingen, Germany, Catalog number: 160 002), and mouse anti-β-actin (Thermo Fisher Scientific, Waltham, Massachusetts, Catalog number: AM4302) antibodies followed by HRP-conjugated secondary antibodies (1:2,000; Sigma-Aldrich). Signals were visualized and analyzed on a Micro Chemi system (DNR Bio-imaging systems, Jerusalem, Israel).

### Subcellular fractionation

Subcellular fractionation was conducted as previously described [159,160]. Briefly, the hippocampal tissues or cultured neurons were homogenized in ice-cold TEVP buffer (10 mM Tris-base (pH 7.4), 1 mM EDTA, 1 mM EGTA, 1 mM Na_3_VO_4_, and 5 mM NaF) containing 320 mM sucrose. The homogenate was centrifuged at 1,000 × *g* for 10 min at 4°C, and the PNS was collected. Synaptosomal pellets were lysed in hypo-osmotic solution, layered on a discontinuous gradient consisting of 0.32, 0.8, 1.0, and 1.2 M sucrose in TEVP buffer and centrifuged at 150,000 × *g* for 2 h at 4°C. The SPMs fraction was collected from the 1.0/1.2 M sucrose interface. Concurrently, the supernatant above the crude synaptosomal fraction was centrifuged at 10,000 × *g* for 70 min to obtain the cytosol and light membranes (S2). Total protein concentrations of the PNS, SPM, and S2 were determined using a BCA kit (Thermo Fisher Scientific) for western blot analysis. The membranes were initially probed with antibodies against phospho-NR1 (p-NR1). After detection, the membranes were stripped and reprobed with antibodies against NMDA (NR1, NR2A, and NR2B) and AMPA (GLUR1 and GLUR2) receptors. All data were corrected for equal protein loading as determined by β-actin detection or Ponceau S staining.

### Quantitative real-time polymerase chain reaction (qPCR)

Total RNA was extracted using the QIAzol reagent (QIAGEN). The cDNA was reverse transcribed from the extracted RNA using a QuantiTect Reverse Transcription Kit (QIAGEN). The gene expression quantified by qPCR experiments were calculated using the 2^−ΔΔCt^ method [161]. The primers used in qPCR analyses of mouse *Lcn2*, *Il1b*, *Tnf*, and *Gapdh* were as follows: *Lcn2*, 5′-ATG TCA CCT CCA TCC TGG TC-3′ (forward), 5′-CAC ACT CAC CAC CCA TTC AG-3′ (reverse); *Il1b*, 5′-AGT TGC CTT CTT GGG ACT GA-3′ (forward), 5′-TCC ACG ATT TCC CAG AGA AC-3′ (reverse); *Tnf*, 5′-CAT CTT CTC AAA ATT CGA GTG ACA A-3′ (forward), 5′-ACT TGG GCA GAT TGA CCT CAG-3′ (reverse); and *Gapdh*, 5′-TGG GCT ACA CTH AHC ACC AG-3′ (forward), 5′-GGG TGT CGC TGT TGA AGT CA-3′ (reverse).

### Microdialysis

Microdialysis was conducted on awake, freely moving mice as previously described [50]. For the optical stimulation coupled with simultaneous microdialysis, mice were stereotaxically implanted with an optical fiber and microdialysis probe guide cannula (AP −2 mm, L ±1.8 mm from the bregma, and V −1.2 mm from the dura). The microdialysis probe (CMA Microdialysis AB, Stockholm, Sweden) was inserted into the CA1 region through the guide cannula, 24 h before starting microdialysis. The probe was connected to a microperfusion pump with polyethylene tubing and perfused with artificial cerebrospinal fluid at a flow rate of 0.5 μl/min for 2 h. Extracellular fluid (ECF) from the outlet of the tube was collected in plastic vials kept on ice. The collected samples were immediately frozen at −80°C until being subjected to ELISA.

### Enzyme-linked immunosorbent assay (ELISA)

The levels of LCN2, IL-1β, and TNF-α in the ECF were quantified using the ELISA kit (R&D Systems). The assays were conducted in 96-well plates using the ECF (1:50 dilution) or cell culture media according to the manufacturer’s instructions. All measurements were obtained from duplicate assays.

### Cell cultures

#### Primary astrocyte culture and optogenetic stimulation

Astrocytes were isolated from mice 1 to 3 days postnatally as previously described [147]. In every 12 wells, 12 μl AAV-GFAP-eYFP and AAV-GFAP-ChR2-eYFP virus was added to 1.2 ml plating medium and mixed, and then 100 μl virus mixture was added to each well. After 7 days of viral infection, the cells were used for the experiments. Primary astrocytes expressing eYFP or ChR2 were illuminated for 20 min with blue light at 460 to 475 nm using a light-emitting diode (LED) illumination system [89]. Cells and culture media were collected after photostimulation for mRNA analysis and ELISA, respectively.

#### Primary hippocampal neurons

Neurons were isolated from the hippocampus of mice at embryonic day 16.5 (E16.5) as previously described [162]. Cultured hippocampal neurons were treated with recombinant LCN2 protein for 30 min, followed by the addition of PBS or glycine (200 μm) and incubated for 30 min. After incubation, the levels of p-NR1, NR1, NR2A, NR2B, PSD-95, GLUR1, GLUR2, Homer, VGLUT2, and β-actin in the PNS and SPM were measured by western blotting and immunostaining.

#### Ca^2+^ imaging in vitro

The Ca^2+^ levels in the primary hippocampal neurons and astrocytes were monitored by a Lionheart automated image analyzer (BioTek) using Fluo-4 AM (5 μm in 0.01% of pluronic, 30-min incubation at 25°C) as previously described [163]. The Ca^2+^ levels were recorded from the hippocampal neurons or astrocyte cell body and processes, and the variations in Ca^2+^ were estimated as changes in the fluorescence signal over the baseline value (*Δ*F/F_0_). The Ca^2+^ event probability was calculated as the number of Ca^2+^ elevations grouped in 5 s bins recorded from the cells in the field of view, and mean values were obtained by averaging the values of each different experiment. For the hippocampal neuron Ca^2+^ imaging, the culture plates were transferred to a submerged recording chamber (37°C), and cells exhibiting Fluor-4 AM were selected for imaging. At 1 min after treatment with recombinant LCN2 protein, PBS or glycine was added to the hippocampal neurons. The Ca^2+^ levels were recorded for 3 min, and variations in Ca^2+^ were estimated as the changes in the fluorescence signal over the baseline value (*Δ*F/F_0_). For the Ca^2+^ imaging of primary astrocytes, astrocytes exhibiting both hM3Dq-mCherry and Fluor-4 AM were selected for imaging. The Ca^2+^ levels were recorded for 3 min (170 s after baseline recording for 10 s), and Ca^2+^ variations were estimated as changes in the fluorescence signal over the baseline value (*Δ*F/F_0_).

#### Neurite length assessment

To measure the neurite length, cells were fixed and processed for confocal microscopy using mouse anti-βIII-tubulin (Santa Cruz, Catalog number: sc-80005) antibody, followed by labeling with FITC-conjugated secondary antibodies. Cells were viewed under the 20× objective of a Zeiss LSM 700 confocal microscope. The coverslips were scanned from left to right, and 8 to 10 fields were randomly selected. For each field, the neurites were traced and measured using Zen software (Zeiss).

### Cell viability assay

Cell viability was determined using the 3-(3,5-dimethylthiazol-2-yl)-2, 5-diphenyltetrazolium bromide (MTT) assay as previously described [36,164]. Hippocampal neurons were treated with PBS, H_2_O_2_, or recombinant LCN2 protein for 24 h, and then cell viability was evaluated in accordance with the manufacturer’s instructions.

### Immunocytochemistry (ICC)

For immunofluorescence analysis of the surface NMDA receptors, primary hippocampal neurons were incubated with mouse anti-NR1 (Abcam, Catalog number: ab134308), rabbit anti-NR2A (Invitrogen, Catalog number: 480031), and rabbit anti-NR2B (Sigma-Aldrich, Catalog number: 06–600) antibodies. Neurons were blocked with antibody solution, placed on ice for 30 min, and fixed in 4% paraformaldehyde and 4% sucrose in PBS for 20 min at room temperature. Next, the neurons were incubated with FITC-conjugated anti-mouse or anti-rabbit antibodies for 1 h at room temperature and mounted in a liquid mounting medium (Vectashield, H-1200), followed by imaging under an Airyscan super resolution confocal microscopy (Carl Zeiss). Surface NMDA receptor image analysis was conducted using the Zen (Carl Zeiss) or ImageJ software, with subsequent quantification being performed using Prism (GraphPad).

### Clozapine-N-oxide (CNO) administration

CNO (Tocris Bioscience, Catalog number: 4936) was dissolved in dimethylsulfoxide (DMSO) and diluted in 0.9% saline to yield a final DMSO concentration of 0.5%. The saline solution used for the control injections also consisted of 0.5% DMSO. Before conducting the behavioral assays, 1 or 3 mg/kg CNO was intraperitoneally injected at 8 h intervals for 24 h. For the in vitro Ca^2+^ imaging, primary astrocytes expressing hM3Dq-mCherry were treated with CNO (10 μm).

### Intracerebroventricular injection of LPS and astrocyte toxin or repeated optogenetic stimulation

Under isoflurane anesthesia, the mice were mounted onto a stereotaxic frame. Two guide cannulas were surgically implanted bilaterally 0.5 mm above the lateral ventricle of the brain. The coordinates for the placement of the guide cannula were 1 mm posterior to the bregma, 1.6 mm lateral, and 2.0 mm below the skull surface at the point of entry. Mice were allowed to recover for a minimum period of 7 days before treatment and initiation of behavioral tests. After recovery, intracerebroventricular (i.c.v.) injections of sterile saline or LPS (1 mg/ml; Sigma-Aldrich). For the astrocyte inhibition experiment, l-α-aminoadipate (l-α-AA, 10 nM; Sigma-Aldrich) was injected 1 h before the LPS injection or 20 min photostimulation. LPS (2 μl) or l-α-AA (2 μl) was administrated intracerebroventricularly at a flow rate of 0.5 μl/min to mice.

### Fiber photometry in vivo

Stereotaxic surgery was performed under isoflurane anesthesia to target the hippocampal CA1 region (A −2 mm, L ±1.8 mm from the bregma, and V −1.2 mm from the dura), and 0.5 μl AAV5-hGFAP-GCaMP6 virus (5 × 10^12^/ml) was infused at a rate of 0.1 μl/min and allowed to diffuse for 10 min before the needle was withdrawn. Chronically implantable optic fibers (Doric Lenses), with a 400-μm core, 0.48 NA optic fiber threaded through metal ferrules, were implanted above the viral injection site. Recordings were initiated a minimum of 14 days after surgery to allow sufficient time for recovery and stable and robust virus expression. The fiber photometry setup was adopted from the methods described by Sych and colleagues [165] with minor modifications. A 470 nm LED operating at 211 Hz was used as the light source for the fiber photometry system. The light passed through a green fluorescent protein (GFP) excitation filter, coupled to an NA, core optical fiber patch cord, and a 0.48 NA, 400 μm fiber optic implant in each mouse. The optical fiber first collected the GCaMP6f fluorescence, which then passed through a GFP emission filter and focused onto a photoreceiver (Newport 2151; Doric Lenses Inc.). The software running on a real-time signal processor (Synapse, RZ5P; Tucker Davis Tech.) controlled the LED and demodulated the incoming signal. A 470 nm LED was modulated at 211 Hz and passed through a 470 nm bandpass filter. The fluorescence signal of the optical fiber was collected with an acquisition rate of 1,017 Hz as the amplified photovoltage from the photoreceiver using the Synapse software (TDT) before being “locked in” and demodulated using the software’s fiber photometry tool. Excitation light at 470 nm passed through a fluorescence mini cube (Doric Lenses), and the fluorescence emitted by GCaMP6f was detected within the 528 to 556 nm window of the device before being delivered through the TDT RZ5P Processor for data acquisition. For the Ca^2+^ recordings in freely moving mice, the relative fluorescence changes [*Δ*F/F = (F-F_baseline_)/F_baseline_] were calculated for Ca^2+^ transients. The peak amplitude, duration, or frequency were analyzed using the photometry modular analysis tool pMAT [166] in MATLAB software version R2021b (MathWorks).

### Statistics

All data are presented as mean ± standard error of the mean (SEM) (in vivo data) or mean ± standard deviation (SD) (in vitro data), as indicated in the figure legends. The Student’s *t* test was used to compare 2 experimental groups for the analysis of quantitative PCR. Significant differences in the electrophysiology data were tested using the Student’s unpaired *t* test. All other datasets were analyzed using a one-way analysis of variance (ANOVA) with Bonferroni’s post hoc tests (in vitro experiments and passive avoidance test), one-way ANOVA with a repeated measurement (Ca^2+^ imaging in vitro and in vivo), two-way ANOVA (probe trials of the Barnes maze task), or two-way ANOVA with a repeated measurement (Y-maze and Barnes maze training) using the SPSS software (version 18.0; SPSS Inc., Chicago, Illinois, USA). ANOVAs followed by Tukey’s multiple comparisons were used to test the differences among multiple groups. Statistical significance was established at *p* < 0.05. The sample size for the experiments was chosen to ensure adequate statistical power based on the G*power 3.1 software [167].

## Supporting information

S1 Fig Astrocytes, but not microglia, express ChR2 in the CA1 hippocampus after viral infection. (**A**) Brain tissue samples following photostimulation were subjected to immunofluorescence analysis to identify the localized ChR2-eYFP (green) expression of astrocytes (GFAP, white) and microglia (Iba-1, red). The nuclei were stained with DAPI (blue). Arrowheads (yellow) indicate the colocalization of ChR2-eYFP and GFAP. The quantification of ChR2-eYFP and Iba-1 colocalization is shown in the adjacent graphs. Scale bar: 200 μm. Results are expressed as mean ± SEM (*n* = 9). n.d., not detected. (**B**) Brain tissue samples were subjected to immunofluorescence analysis to localize the expression of eYFP and ChR2-eYFP (green) in astrocytes (GFAP, red). The nuclei were stained with DAPI (blue). Arrowheads (yellow) indicate the colocalization of eYFP or ChR2-eYFP with GFAP in the no-photostimulation control animals. (**C**) ChR2-eYFP expression in astrocytes was further confirmed in reconstructed confocal Z-section images. Scale bar: 20 μm. The results are representative of the 6 experiments (*n* = 6). Source data can be found in S1 Data.
(TIFF)

S2 Fig Either photostimulation of CA1 astrocytes for 20 min without virus injection or no-surgery control had no significant effects on cognitive function of animals. (**A**) Experimental timeline of viral injection, surgery, optogenetic stimulation. Mice were injected with PBS. On day 14 post-injection and surgery, optogenetic stimulations (3 times) were delivered for 20-min to the hippocampal CA1 region. Blue and black arrows indicate the time points for optogenetic stimulation and behavioral testing, respectively. (**B**) The cognitive behavior of no-surgery group or surgery without virus injection group after photostimulation was analyzed by Y-maze tests (*n* = 5). Results are expressed as mean ± SEM (*n* = 5). n.s., not significant (one-way ANOVA). Source data can be found in S1 Data.
(TIFF)

S3 Fig Optogenetic stimulation of CA1 astrocytes for 5- or 10-min had no significant effect on cognitive function. Experimental timeline of viral injection, surgery, optogenetic stimulation, and behavioral analysis. Mice were injected with AAV-GFAP-eYFP or AAV-GFAP-ChR2-eYFP. On day 14 post-injection and surgery, the first (day 1, in the timeline), second (day 2, in the timeline), and third (day 3, in the timeline) optogenetic stimulations were delivered for 5- or 10-min to the hippocampal CA1 region. Blue and black arrows indicate the time points for optogenetic stimulation and behavioral testing, respectively. The cognitive behavior of the eYFP or ChR2-eYFP expressing mice after optogenetic stimulation of the hippocampal astrocytes was analyzed by Y-maze (*n* = 10) (**A**), Barnes maze (*n* = 5) (**B**), and passive avoidance (*n* = 10) (**C**) tests. Results are expressed as mean ± SEM (*n* = 5 or 10). *p < 0.05 between the indicated groups; n.s., not significant (two-way ANOVA in **A** and **B**, one-way ANOVA in **C**). Source data can be found in S1 Data.
(TIFF)

S4 Fig Photostimulation-induced cognitive impairment was not further enhanced through repeated optogenetic stimulation. (**A**) Experimental timeline of viral injection, surgery, optogenetic stimulation, and behavioral analysis. Adenovirus expressing ChR2 under the control of astrocyte-specific GFAP promoter (Ad-GFAP-ChR2-Katushka1.3; Ad-ChR2) was used for the injection. The optic fibers and cannula were implanted above the hippocampal CA1 area for photostimulation. Seven days (day 1, in the timeline) after Ad-GFP or Ad-ChR2 viral injection, photostimulation was delivered for the duration of 20 min through the optogenetic fiber. Blue and black arrows indicate the time points of optogenetic stimulation and behavioral testing, respectively. (**B**) Ad-ChR2 injected WT mice (WT-ChR2) showed an apparent impairment of spatial memory compared with Ad-GFP-injected control animals (WT-GFP) through optogenetic stimulation. The optogenetic stimulation-induced spatial memory impairment was significantly attenuated in the Ad-ChR2 injected *Lcn2*-KO mice (*Lcn2* KO-ChR2). There was no significant difference in the locomotor activity as determined by the number of entries. Results are expressed as mean ± SEM (*n* = 10). *p < 0.05, WT-GFP versus WT-ChR2; #*p* < 0.05, WT-ChR2 versus *Lcn2* KO-ChR2; n.s., not significant (two-way ANOVA). Source data can be found in S1 Data.
(TIFF)

S5 Fig Optogenetic stimulation of the CA1 astrocytes for 20 min during the memory acquisition impaired the cognitive function. (**A**) Experimental timeline of viral injection, surgery, optogenetic stimulation, and behavioral analysis. Mice were injected with AAV-GFAP-eYFP or AAV-GFAP-ChR2-eYFP. On day 14 post-injection, optogenetic stimulations were delivered for 5-, 10-, or 20-min to the hippocampal CA1 region during the memory acquisition in the training phase. Blue and black lines indicate the time points for optogenetic stimulation and behavioral testing, respectively. (**B**–**D**) The cognitive behavior of eYFP or ChR2-eYFP expressing mice after the optogenetic stimulation of hippocampal astrocytes was analyzed by the Barnes maze test. Results are expressed as mean ± SEM (*n* = 5). *p < 0.05 between the indicated groups; n.s., not significant (two-way ANOVA). Source data can be found in S1 Data.
(TIFF)

S6 Fig Optogenetic stimulation of CA1 astrocytes for 20 min after memory acquisition impairs cognitive function. (**A**) Experimental timeline of viral injection, surgery, optogenetic stimulation, and behavioral analysis. Mice were injected with AAV-GFAP-eYFP or AAV-GFAP-ChR2-eYFP. On day 14 post-injection, optogenetic stimulations were delivered for 20 min to the hippocampal CA1 region after memory acquisition in the training phase. Blue and black lines indicate the time points of the optogenetic stimulation and behavioral testing, respectively. (**B**) The cognitive behavior of eYFP or ChR2-eYFP-expressing mice after the optogenetic stimulation of hippocampal astrocytes was analyzed by the Barnes maze test. Results are expressed as mean ± SEM (*n* = 5). *p < 0.05 between the indicated groups; n.s., not significant (two-way ANOVA). Source data can be found in S1 Data.
(TIFF)

S7 Fig Repeated optogenetic stimulation of hippocampal astrocytes either before or after memory acquisition induces cognitive impairment. Experimental timeline of viral injection, surgery, optogenetic stimulation, and behavioral analysis (**A** and **B,** upper). Mice were injected with AAV-GFAP-eYFP or AAV-GFAP-ChR2-eYFP. On day 14 post-injection (day 1, in the timeline), the first (day 1, in the timeline), second (day 2, in the timeline), and third (day 3, in the timeline) optogenetic stimulations were delivered for 20 min to the hippocampal CA1 region, after (**A**) or before (**B**) the training stages. Blue and black arrows indicate the time points for the optogenetic stimulation and behavioral testing, respectively. The cognitive behavior of eYFP or ChR2-eYFP-expressing mice after the optogenetic stimulation of the hippocampal astrocytes was analyzed by passive avoidance (*n* = 6) (**A** and **B,** lower) tests. Results are expressed as mean ± standard deviation of the mean (SEM) (*n* = 6). *p < 0.05 between the indicated groups; n.s., not significant (one-way ANOVA). Source data can be found in S1 Data.
(TIFF)

S8 Fig Optogenetic stimulation of hippocampal astrocytes has no significant effect on the basal excitatory synaptic transmission. (**A**) Experimental timeline of viral injection, surgery, optogenetic stimulation, and electrophysiological experiments. (**B**) Typical traces of the fEPSPs evoked by various strengths of stimuli in the eYFP and ChR2-eYFP expressing groups (left). The relationship between the fiber volley amplitude (input) and fEPSP amplitude (output) in the eYFP (open circles, *n* = 4) and ChR2-eYFP expressing groups (closed circles, *n* = 4) (right). To examine the input–output relationship, the fEPSPs were recorded in the presence of 3 μm CNQX to reduce the fEPSP amplitude. (**C**) Typical traces of the fEPSPs evoked by paired-pulse stimuli with various intervals in the eYFP (left upper) and ChR2-eYFP expressing groups (left lower). The paired-pulse ratio of the fEPSPs in the eYFP (open circles, *n* = 4) and ChR2-eYFP expressing groups (closed circles, *n* = 4) (right). (**D**) Typical traces of the mEPSCs recorded from the hippocampal CA1 neurons in the eYFP (left upper) and ChR2-eYFP expressing groups (left lower). The mean amplitude (middle) and frequency (right) of the mEPSCs in the eYFP (*n* = 7) and ChR2-eYFP expressing groups (*n* = 15). Results are expressed as mean ± SEM. n.s., not significant (unpaired *t* test). Source data can be found in S1 Data.
(TIFF)

S9 Fig Effect of short-term optogenetic stimulation of the hippocampal CA1 astrocytes on the hippocampal LTP. (**A**) Time courses of the fEPSP responses before and after TBS from the hippocampal slices in the eYFP (gray circles, *n* = 10 from 5 mice) and ChR2-eYFP groups (green circles, *n* = 10 from 5 mice). In these experiments, photostimulation was applied for 5 min. TBS was applied for LTP induction at 30 min. Insets represent the typical raw traces from the average of 6 successive fEPSPs recorded at the time indicated by arrowheads with numbered regions (1; black or 2; red). (**B**) TBS-induced LTP in eYFP (*n* = 10 from 5 mice) and ChR2-eYFP (*n* = 10 from 5 mice) groups. The mean fEPSP slope during the 50–60 min after TBS was quantified as the LTP level. Results are expressed as mean ± SEM. n.s., not significant (unpaired *t* test). Source data can be found in S1 Data.
(TIFF)

S10 Fig Effect of 5-min photostimulation of hippocampal CA1 astrocytes on learning, LCN2 protein, and hippocampal LTP. (**A**) Experimental timeline of the viral injection, surgery, optogenetic stimulation, and behavioral analysis. Mice were injected with AAV-GFAP-eYFP or AAV-GFAP-ChR2-eYFP. On day 14 post-injection, optogenetic stimulations were delivered for 5 min to the hippocampal CA1 region during memory acquisition in the training phase. Blue, black, and yellow lines indicate the time points of optogenetic stimulation, behavioral testing, and microdialysis, respectively. (**B**) Cognitive behavior of the eYFP or ChR2-eYFP-expressing mice groups after optogenetic stimulation of the hippocampal astrocytes was analyzed by the Barnes maze test. Results are expressed as mean ± SEM (*n* = 4). *p < 0.05, eYFP versus ChR2-eYFP groups; n.s., not significant (two-way ANOVA). (**C**) The 5-min photostimulation during memory acquisition of hippocampal CA1 astrocytes did not significantly affect the extracellular LCN2 release at day 2. The LCN2 levels in the dialysate were measured by ELISA. Results are expressed as mean ± SEM (*n* = 5). n.s., not significant (one-way ANOVA). (**D**) Time courses of the fEPSP responses before and after the TBS from the hippocampal sections in the eYFP (gray, *n* = 10 from 5 mice) and ChR2-eYFP (green, *n* = 10 from 5 mice) groups. In these experiments, photostimulation was applied for 5 min before behavioral training. TBS was applied for LTP induction at 30 min. Insets represent the typical raw traces from the average of 6 successive fEPSPs recorded at the time indicated by the arrowheads with numbered regions (1; black or 2; red). (**E**) TBS-induced LTP in the eYFP (*n* = 10 from 5 mice) and ChR2-eYFP (*n* = 9 from 5 mice) groups. The mean fEPSP slope during the 50 to 60 min after TBS was quantified as the LTP level. Results are expressed as mean ± SEM. n.s., not significant (unpaired *t* test). Source data can be found in S1 Data.
(TIFF)

S11 Fig Optogenetic stimulation of the hippocampal CA1 astrocytes does not significantly affect the expression of AMPA receptors or VGLUT2. (**A**) Experimental timeline of the viral injection, surgery, optogenetic stimulation, and behavioral analysis. (**B**) AMPA receptors (GLUR1 and GLUR2) and vesicular glutamate transporter 2 (VGLUT2) protein levels were measured by western blotting. Quantification of the band intensities is presented in the adjacent graphs. Results are expressed as mean ± SD (*n* = 4). n.s., not significant (one-way ANOVA). (**C**) Western blot analysis of GLUR1, GLUR2, and VGLUT2 present in the subcellular fractions (PNS and SPM) obtained from the hippocampal tissues of the eYFP and ChR2-eYFP expressing mice. Quantification of GLUR1, GLUR2, or VGLUT2 in the PNS was based on normalization against the internal control β-actin and that of GLUR1, GLUR2, or VGLUT2 in the SPM was normalized against the internal control Ponceau S (S13B Fig) and are represented as graphs for the blots. Results are expressed as mean ± SD (*n* = 4). n.s., not significant (one-way ANOVA). Source data can be found in S1 Data.
(TIFF)

S12 Fig Assessment of the membrane NMDA and AMPA receptor expression after optogenetic stimulation of hippocampal CA1 astrocytes. (**A**) Schematic depiction of the centrifugation steps to produce the PNS, SPM, and S2 from hippocampal tissue samples. Western blot analysis of p-NR1, NR1, NR2A, NR2B, GLUR1, and GLUR2 present in each subcellular fraction (PNS, SPM, and S2) obtained from the hippocampal tissues of eYFP- or ChR2-eYFP-expressing mice (at 5-, 10-, and 20-min photostimulation). Quantification of p-NR1, NR1, NR2A, or NR2B in the PNS and S2 was based on the normalization against the internal control β-actin, and that for p-NR1, NR1, NR2A, NR2B, GLUR1, or GLUR2 in the SPM was normalized against the internal control Ponceau S and represented as graphs for the blots. The Ponceau S-stained membranes are shown in S13E Fig. Results are expressed as mean ± SD (*n* = 3). *p < 0.05 versus eYFP (20 min) control groups; n.s., not significant (one-way ANOVA). (**B**) mRNA levels of NMDA receptors (*Grin1*, *Grin2a*, and *Grin2b*) were measured by qPCR. Results are expressed as mean ± SD (*n* = 5). n.s., not significant (one-way ANOVA). The SPM contains cell surface membranes, and S2 contains intracellular organelle membranes and cytosol. Source data can be found in S1 Data.
(TIFF)

S13 Fig Total protein staining (Ponceau S) confirmed equal loading in western blot analysis. The same amounts of PNS, SPM, and S2 from the hippocampal tissue (**A, B,** and **E**) or cultured hippocampal neurons (**C**, **D**, and **F**) were resolved by SDS-PAGE, as shown on Ponceau S-stained blots. Quantification of each protein in the SPM was normalized to Ponceau S and represented as graphs in each figure indicated. (**A**) Ponceau S staining of Fig 2H. (**B**) Ponceau S staining of S11C Fig. (**C**) Ponceau S staining of S16C Fig. (**D**) Ponceau S staining of Fig 4C. (**E**) Ponceau S staining of S12A Fig, (**F**) and Ponceau S staining of S16B Fig.
(TIFF)

S14 Fig Optogenetic stimulation of cultured astrocytes increases the expression of *Lcn2* and proinflammatory cytokines. (**A**) Experimental timeline of viral injection, surgery, optogenetic stimulation, and behavioral analysis. (**B**) Phase contrast (Ph) and fluorescence images of astrocytes in the primary cultures infected with AAV-GFAP-eYFP (green) or AAV-GFAP-ChR2-eYFP (green). The number of cells and their co-localization (%) are shown. Quantification of the eYFP-positive cell colocalization is shown in the adjacent graphs. Scale bar: 200 μm. Results are expressed as mean ± SEM (*n* = 3). n.d., not detected. (**C**) Primary astrocyte cultures were illuminated using the LED device for 20 min, and total RNA was extracted after 6 h. The mRNA levels of *Lcn2*, *Il1b*, and *Tnf* were determined by qPCR (upper, no photostimulation; lower, photostimulation). Data were normalized to the internal control *Gapdh*, and results are expressed as mean ± SD (*n* = 3) in the graphs. *p < 0.05 between the indicated groups; n.s., not significant (one-way ANOVA). (**D**) The levels of LCN2 protein secretion in the culture media were measured by ELISA at 24 h after photostimulation. Results are expressed as mean ± SEM (*n* = 6). *p < 0.05 between the indicated groups; n.s., not significant (one-way ANOVA). (**E**) Cell viability was measured using an MTT assay at 24 h after the photostimulation. Results are expressed as mean ± SEM (*n* = 5). *p < 0.05 between the indicated groups; n.s., not significant (one-way ANOVA). Source data can be found in S1 Data.
(TIFF)

S15 Fig Ca^2+^ imaging in the hippocampal neurons treated with LCN2 protein. (**A**) Experimental timeline of viral injection, surgery, optogenetic stimulation, and behavioral analysis. (**B**) Fluo-4 AM-loaded neurons were stimulated with glycine (200 μm) (for each group in the presence of PBS), denatured LCN2 (dLCN2, 10 ng/ml), and LCN2 (10 pg/ml, 1 ng/ml, and 10 ng/ml), and Ca^2+^ transient was analyzed by Lionheart FX automated imaging analyzer. Results are expressed as mean ± SD (*n* = 4 or 7). n.s., not significant, PBS versus dLCN2; *p < 0.05, dLCN2 versus LCN2 (10 pg/mL); #*p* < 0.05, dLCN2 versus LCN2 (1 ng/ml); §p < 0.05, dLCN2 versus LCN2 (1 ng/ml) (one-way ANOVA). Source data can be found in S1 Data.
(TIFF)

S16 Fig LCN2 treatment does not significantly alter the membrane expression of AMPA receptors or VGLUT2 in the hippocampal neurons. (**A**) Experimental timeline for cultured hippocampal neurons were stimulated with vehicle or glycine (200 μm) after incubation with PBS, denatured LCN2 (dLCN2, 10 ng/ml), or LCN2 (1 ng/ml) protein. (**B**) Western blot analysis of GLUR1, GLUR2, Homer, or VGLUT2 present in PNS and SPM obtained from the hippocampal neurons under each condition. Quantification of GluR1, GluR2, Homer, or VGLUT2 in PNS was based on normalization against the internal control β-actin, and that of GLUR1, GLUR2, Homer, or VGLUT2 in SPM was normalized against the internal control Ponceau S (S13C Fig) and represented as graphs for the blots. Results are expressed as mean ± SD (*n* = 4). *p < 0.05 between the indicated groups; n.s., not significant (one-way ANOVA). (**C**) Western blot analysis of GLUR1 or GLUR2 present in SPM obtained from the hippocampal neurons after glycine treatment. Quantification of GLUR1 or GLUR2 in SPM was normalized against the internal control Ponceau S (S13F Fig) and represented as graphs for the blots. Results are expressed as mean ± SD (*n* = 4). *p < 0.05 between the indicated groups (one-way ANOVA). Source data can be found in S1 Data.
(TIFF)

S17 Fig LCN2 has no significant effect on neurite length or neurotoxicity in the cultured hippocampal neurons. (**A**) Experimental timeline. Hippocampal neurons in media with or without glycine (200 μm), in the presence of PBS, denatured LCN2 (dLCN2, 10 ng/ml), or LCN2 (1 ng/ml) protein. Scale bar: 50 μm. Mean neurite length was determined from 6–7 cells under each culture condition obtained from 3 separate experiments. LCN2 (1 ng/ml) had no effect on neurite length in the hippocampal neurons. Results are expressed as mean ± SD (*n* = 6 or 7). n.s., not significant, between the indicated groups (one-way ANOVA). (**B**) No significant effect of LCN2 (10 pg/ml and 1 ng/ml) on cell viability. Hydrogen peroxide (H_2_O_2_, 500 μm) or LCN2 (1 μg/ml) was used as a positive control. Results are expressed as mean ± SD (*n* = 6). *p < 0.05 between the indicated groups; n.s., not significant (one-way ANOVA). Source data can be found in S1 Data.
(TIFF)

S18 Fig Validation of the ChR2 expression within the hippocampal CA1 region in wild-type (WT) and *Lcn2*-KO mice and the optogenetic induction of *Lcn2* expression in WT mice. (**A**) Experimental timeline of viral injection, surgery, optogenetic stimulation, and behavioral analysis. (**B**) Brain tissue samples from the WT and *Lcn2*-KO mice were subjected to immunofluorescence analysis to localize the expression of the eYFP (green) and ChR2-eYFP (green) in astrocytes (GFAP, red). The nuclei were stained with DAPI (blue). Arrowheads (yellow) indicate the colocalization of eYFP, ChR2-eYFP, and GFAP. Quantification of the eYFP, ChR2-eYFP, and GFAP colocalization is shown in the adjacent graphs. Scale bar: 100 μm. Results are expressed as mean ± SEM (*n* = 9). (**C**) Total mRNA was extracted from the hippocampal tissue of each group after photostimulation in the eYFP and ChR2-eYFP expressing WT mice and subjected to qPCR to evaluate the expression levels of *Lcn2*. *Gapdh* was used as an internal control. Results are expressed as mean ± SEM (*n* = 9). *p < 0.05 between the indicated groups (Student’s *t* test). Source data can be found in S1 Data.
(TIFF)

S19 Fig Selective expression of ChR2-eYFP in the hippocampal CA1 astrocytes. Brain tissue samples were subjected to immunofluorescence analysis to localize the expression of the ChR2-eYFP (green) in astrocytes (GFAP, red, **A**) and microglia (Iba-1, red, **B**). The nuclei were stained with DAPI (blue). Arrowheads (yellow) indicate the colocalization of ChR2-eYFP and GFAP-positive astrocytes. The adjacent graph displays the quantification of fluorescence intensity (astrocytes, **A**; microglia, **B**) in the whole hippocampus, CA1, or DG region. Scale bar: 500 μm (low magnification), and 100 μm (high magnification). Results are expressed as mean ± SEM (*n* = 4). *p < 0.05 between the indicated groups; n.s., not significant (one-way ANOVA). Source data can be found in S1 Data.
(TIFF)

S20 Fig Alteration in microglial morphological features following optogenetic astrocyte stimulation. (**A**) Experimental timeline. (**B**) Hippocampal CA1 sections stained with anti-Iba-1 antibody and DAPI show various morphological changes (somatic volume, process number, and total process length) in microglia following optogenetic astrocyte stimulation. These morphological changes in microglia are attenuated by *Lcn2* deficiency. Scale bar: 200 μm. Results are presented as mean ± SEM (*n* = 4). *p < 0.05 between the indicated groups; n.s., not significant (one-way ANOVA). Source data can be found in S1 Data.
(TIFF)

S21 Fig Characterization of the short- and long-term synaptic plasticity in *Lcn2*-KO mice. (**A**) Typical traces of the fEPSPs evoked by various strengths of stimuli in the WT (left) and *Lcn2*-KO (right) mice. To examine the input–output relationship, the fEPSPs were recorded in the presence of 3 μm CNQX to reduce the fEPSP amplitude. The relationship between the fiber volley amplitude (input) and fEPSP amplitude (output) in the WT (gray circles, *n* = 6 from 5 mice) and *Lcn2*-KO (yellow circles, *n* = 6 from 5 mice) mice. Results are expressed as mean ± SEM. (**B**) Typical traces of fEPSPs evoked by the paired-pulse stimuli with various intervals in the WT (upper) and *Lcn2*-KO (lower) mice. The paired-pulse ratio of fEPSPs in WT (gray circles, *n* = 6 from 5 mice) and *Lcn2*-KO (yellow circles, *n* = 6 from 5 mice) mice. Results are expressed as mean ± SEM. (**C**) Time courses of the fEPSP responses before and after the TBS from the hippocampal sections of the WT (gray circles, *n* = 7 from 5 mice) or *Lcn2*-KO (yellow circles, *n* = 8 from 5 mice) mice. The values were normalized in each experiment to the mean amplitude value measured during the control period (20–30 min). TBS was applied for the LTP induction at 30 min. Insets represent the typical raw traces from the average of 6 successive fEPSPs recorded at the time indicated by the arrowheads with numbered regions (1; black or 2; red). TBS-induced LTP in the WT (gray circles, *n* = 7 from 5 mice) or *Lcn2*-KO (yellow circles, *n* = 8 from 5 mice) mice. The mean fEPSP slope during 50–60 min after TBS was quantified as the LTP level. Results are expressed as mean ± SEM. n.s., not significant (unpaired *t* test). (**D**) Time courses of the fEPSP responses before and after PP-LFS from the hippocampal sections of the WT (gray circles, *n* = 8 from 5 mice) or *Lcn2*-KO (yellow circles, *n* = 8 from 5 mice) mice. PP-LFS was applied for LTD induction at 20 min. Insets represent the typical raw traces from the average of 6 successive fEPSPs were recorded at the time indicated by the arrowheads with numbered regions (1; black or 2; red). The values were normalized in each experiment to the mean amplitude value measured during the control period (10–20 min). PP-LFS-induced LTD in the WT (gray bar, *n* = 8 from 5 mice) or *Lcn2*-KO (yellow bar, *n* = 8 from 5 mice) mice. The mean fEPSP slope during 35–40 min after PP-LFS was quantified as the LTD level. Results are expressed as mean ± SEM. n.s., not significant (unpaired *t* test). (**E**) Typical traces of mEPSCs recorded from the CA1 pyramidal neurons of the WT (upper) and *Lcn2*-KO (lower) mice. The mean frequency (left) and amplitude (right) of mEPSCs were recorded from the CA1 pyramidal neurons of the WT (*n* = 7 from 5 mice) and *Lcn2*-KO (*n* = 7 from 5 mice) mice. Results are expressed as mean ± SEM. n.s., not significant (unpaired *t* test). (**F**) Typical traces of action potential-dependent EPSCs were recorded from the CA1 pyramidal neurons of the WT (upper) and *Lcn2*-KO (lower) mice. The AMPA receptor-mediated (inward currents; EPSC_AMPA_) and NMDA receptor-mediated (outward currents; EPSC_NMDA_) of the EPSCs were recorded at a holding potential of −60 and +20 mV, respectively. The amplitude ratio of AMPA and NMDA receptor-mediated currents in the WT (*n* = 7 from 5 mice) and *Lcn2*-KO (*n* = 7 from 5 mice) mice. Results are expressed as mean ± SEM. n.s., not significant (unpaired *t* test). (**G** and **H**) Optogenetic stimulation of hippocampal CA1 astrocytes does not significantly affect LTP expression levels in *Lcn2*-KO mice. Time courses of fEPSP responses before and after TBS from hippocampal sections in eYFP (gray) and ChR2-eYFP (green) groups. In these experiments, photostimulation was applied for 0 min (**G**: eYFP, gray, *n* = 7 from 3 *Lcn2*-KO mice; ChR2-eYFP, green, *n* = 6 from 3 *Lcn2*-KO mice) or 20 min (**H**: eYFP, gray, *n* = 7 from 3 *Lcn2*-KO mice; ChR2-eYFP, green, *n* = 7 from 3 *Lcn2*-KO mice). TBS was applied for LTP induction at 30 min. Insets show typical raw traces averaged from 6 successive fEPSPs recorded at times indicated by arrowheads in numbered regions (1; black or 2; green). The mean fEPSP slope (left) during the 50–60 min following TBS was quantified as the level of LTP (%) shown in the graph (right). Results are presented as mean ± SEM. n.s., not significant (unpaired *t* test). Source data can be found in S1 Data.
(TIFF)

S22 Fig Chemogenetic stimulation of the cultured astrocytes increased the intracellular Ca^2+^ levels. (**A**) Experimental timeline. The hM3Dq-mCherry expressing primary astrocytes loaded with Fluo-4 AM exhibited transient Ca^2+^ after treatment with CNO (10 μm). CNO was added at 10 s. Ca^2+^ levels in the non-infected or eYFP groups were not significantly altered. Results are expressed as mean ± SD (*n* = 3). *p < 0.05, eYFP versus hM3Dq-mCherry (10–160 s) (one-way ANOVA). (**B**) Representative images showing the Ca^2+^ transients. Scale bar: 500 μm. Source data can be found in S1 Data.
(TIFF)

S23 Fig Ca^2+^ transient activities of hippocampal astrocytes in the neuroinflammation model. (**A**) Experimental timeline of virus injection to the hippocampal CA1 region, optical fiber implantation (for fiber photometry), and LPS injection (2 μg, i.c.v.). (**B**) Heatmap illustrates the GCaMP6f fluorescence (*ΔF/F* %) of the individual mice from −5 s to 35 s in response to PBS or LPS injection (yellow arrows and vertical dotted lines) (left). The plots illustrate the average fluorescence of 3 mice for each group (middle). The curves and shaded regions indicate the mean ± SEM (*n* = 3). The adjacent graph shows the averaged peak amplitude (right); average peak amplitude from 5 to 15 s (indicated by red line in the plot) was normalized to a baseline value from −5 to 0 s (indicated by blue line in the plot). Results are expressed as mean ± SEM (*n* = 3). *p < 0.05 between the indicated groups (one-way ANOVA). (**C**) Experimental timeline of GCaMP6f fluorescence recording in the hippocampal CA1 region after LPS administration. The heatmap and traces show the GCaMP6f fluorescence changes at baseline (at −24 h before LPS injection; recorded for 5 min) and after LPS injection (at 0, 3, and 24 h after LPS injection; recorded for 5 min). The plots show the *Δ*F/F (green trace), peak threshold (red dotted line), and the start and end of each peak that was detected (blue trace; lower). The adjacent graph displays the average peak frequency, duration, and amplitude before and after LPS administration. Results are expressed as mean ± SEM (*n* = 3). *p < 0.05 between the indicated groups; n.s., not significant (one-way ANOVA). (**D**) A schematic illustration of the experimental setup (left). Brain tissue samples were subjected to immunofluorescence analysis to localize the GCaMP6f (green) expression in astrocytes (GFAP, white) (right). The nuclei were stained with DAPI (blue). Arrowheads indicate the colocalization of GCaMP6f and GFAP. Quantification of GFAP and GCaMP6f colocalization is shown in the bar graphs. Scale bar: 200 μm. Results are expressed as mean ± SEM (*n* = 3). n.d., not detected. Source data can be found in S1 Data.
(TIFF)

S1 Table Comparison of current and previous studies using optogenetics or chemogenetics to manipulate astrocytes for interrogating cognitive function. (PDF)

S1 Data Presentation in graphs. (XLSX)

S1 Raw Images Raw gel images. (PDF)

## Abbreviations

AD: Alzheimer’s disease
AAV: adeno-associated virus
cLTP: chemical long-term potentiation
CNO: clozapine-N-oxide
CNS: central nervous system
DG: dentate gyrus
ECF: extracellular fluid
ELISA: enzyme-linked immunosorbent assay
EPSC: excitatory postsynaptic current
fEPSP: field excitatory postsynaptic potential
GFP: green fluorescent protein
ICC: immunocytochemistry
LCN2: lipocalin-2
LED: light-emitting diode
LPS: lipopolysaccharide
LTD: long-term depression
LTP: long-term potentiation
mEPSC: miniature excitatory postsynaptic current
PBS: phosphate buffered saline
PNS: postnuclear supernatant
PP-LFS: paired-pulse low-frequency stimulation
PSD: postsynaptic density
PVDF: polyvinylidene difluoride
SD: standard deviation
SEM: standard error of the mean
SPM: synaptic plasma membrane
TBS: theta burst stimulation
WT: wild-type
