# Supplementary figures and images for: Aberrant activation of hippocampal astrocytes causes neuroinflammation and cognitive decline in mice
Source: PLoS Biol. 2024 Jul 11;22(7):e3002687. doi: 10.1371/journal.pbio.3002687 (PMC11239238; doi:10.1371/journal.pbio.3002687)

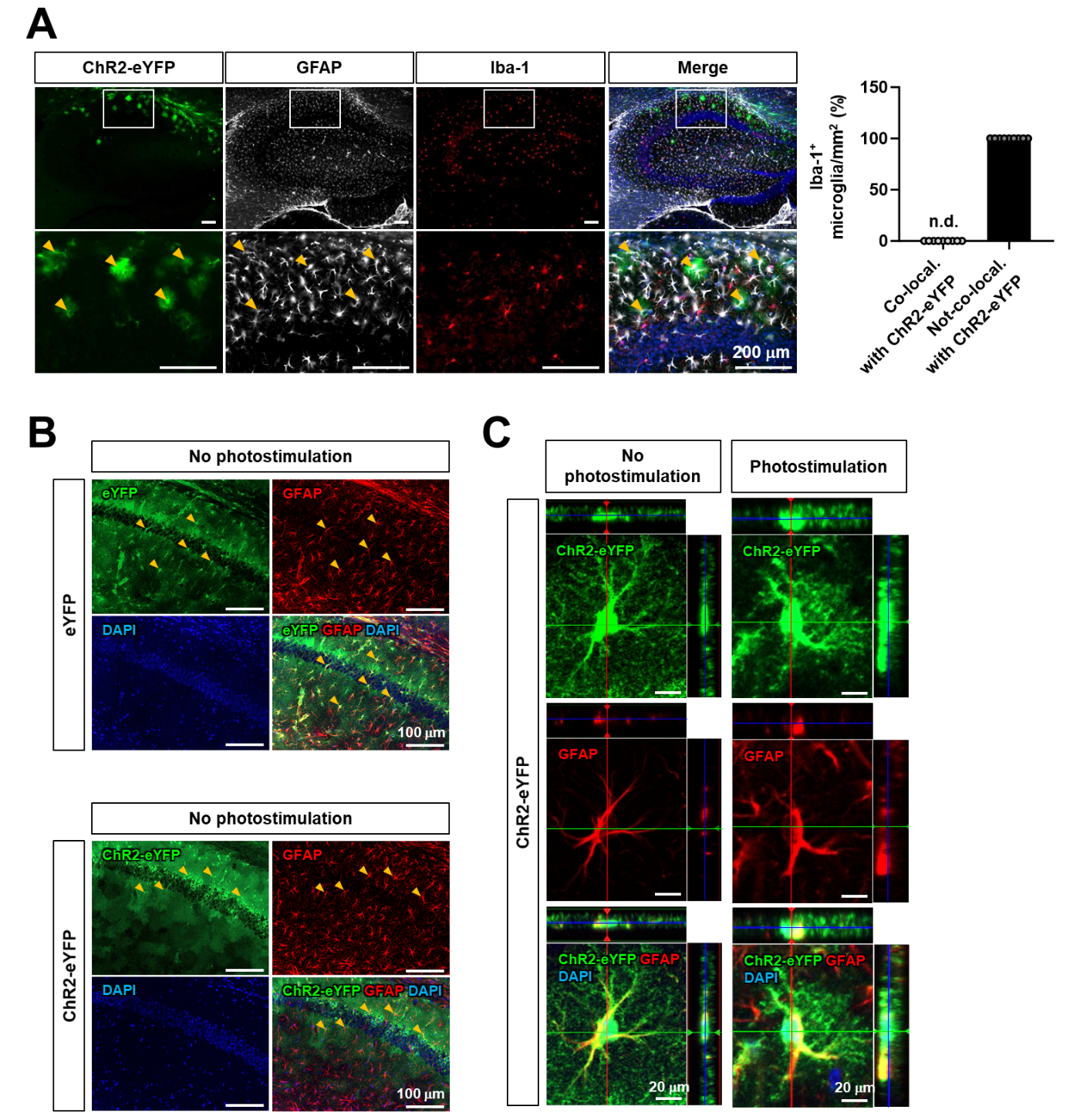

Supplement: S1 Fig — (A) Brain tissue samples following photostimulation were subjected to immunofluorescence analysis to identify the localized ChR2-eYFP (green) expression of astrocytes (GFAP, white) and microglia (Iba-1, red). The nuclei were stained with DAPI (blue). Arrowheads (yellow) indicate the colocalization of ChR2-eYFP and GFAP. The quantification of ChR2-eYFP and Iba-1 colocalization is shown in the adjacent graphs. Scale bar: 200 μm. Results are expressed as mean ± SEM (n = 9). n.d., not detected. (B) Brain tissue samples were subjected to immunofluorescence analysis to localize the expression of eYFP and ChR2-eYFP (green) in astrocytes (GFAP, red). The nuclei were stained with DAPI (blue). Arrowheads (yellow) indicate the colocalization of eYFP or ChR2-eYFP with GFAP in the no-photostimulation control animals. (C) ChR2-eYFP expression in astrocytes was further confirmed in reconstructed confocal Z-section images. Scale bar: 20 μm. The results are representative of the 6 experiments (n = 6). Source data can be found in S1 Data. (TIFF) [file pbio.3002687.s001.tiff]

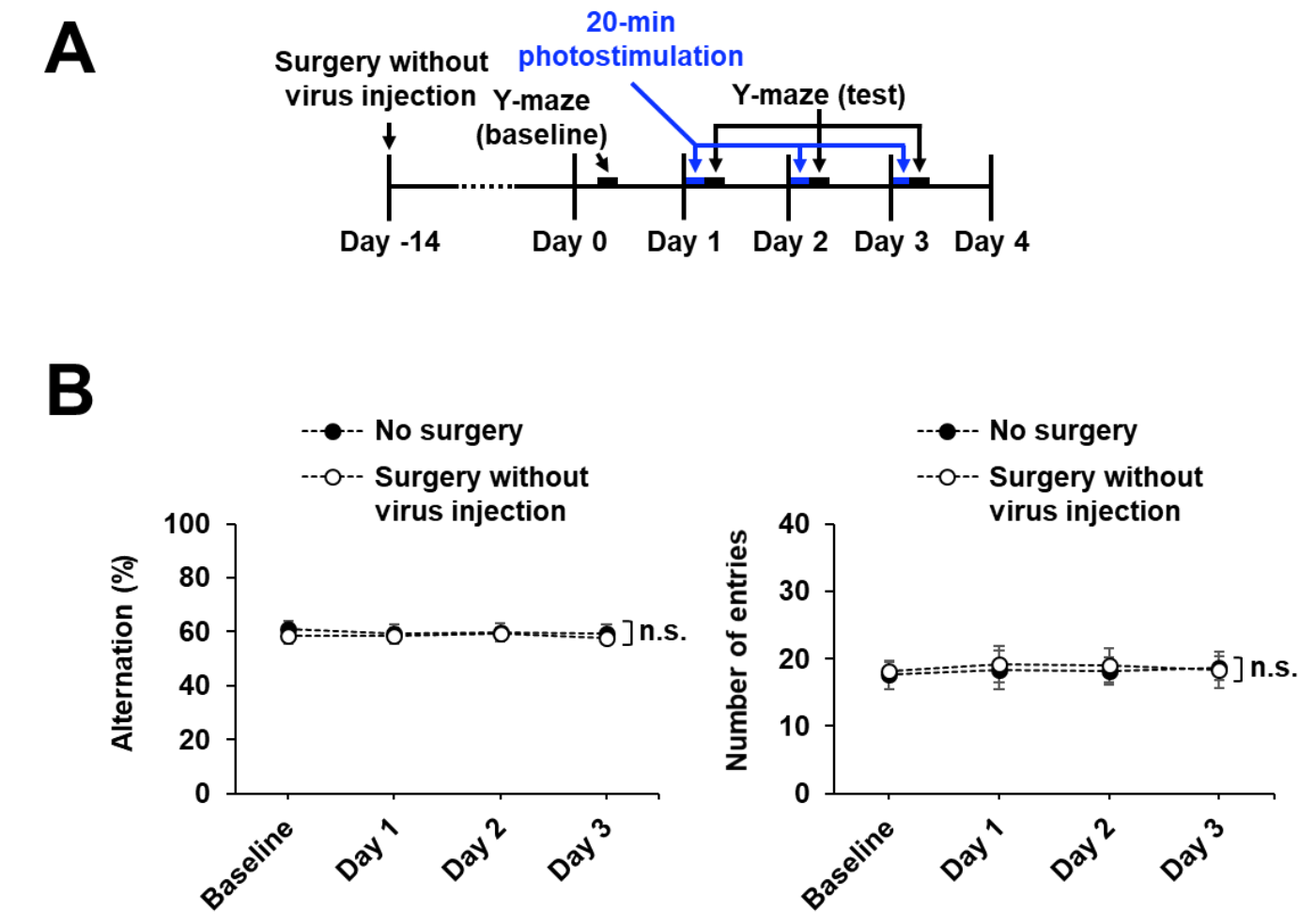

Supplement: S2 Fig — (A) Experimental timeline of viral injection, surgery, optogenetic stimulation. Mice were injected with PBS. On day 14 post-injection and surgery, optogenetic stimulations (3 times) were delivered for 20-min to the hippocampal CA1 region. Blue and black arrows indicate the time points for optogenetic stimulation and behavioral testing, respectively. (B) The cognitive behavior of no-surgery group or surgery without virus injection group after photostimulation was analyzed by Y-maze tests (n = 5). Results are expressed as mean ± SEM (n = 5). n.s., not significant (one-way ANOVA). Source data can be found in S1 Data. (TIFF) [file pbio.3002687.s002.tiff]

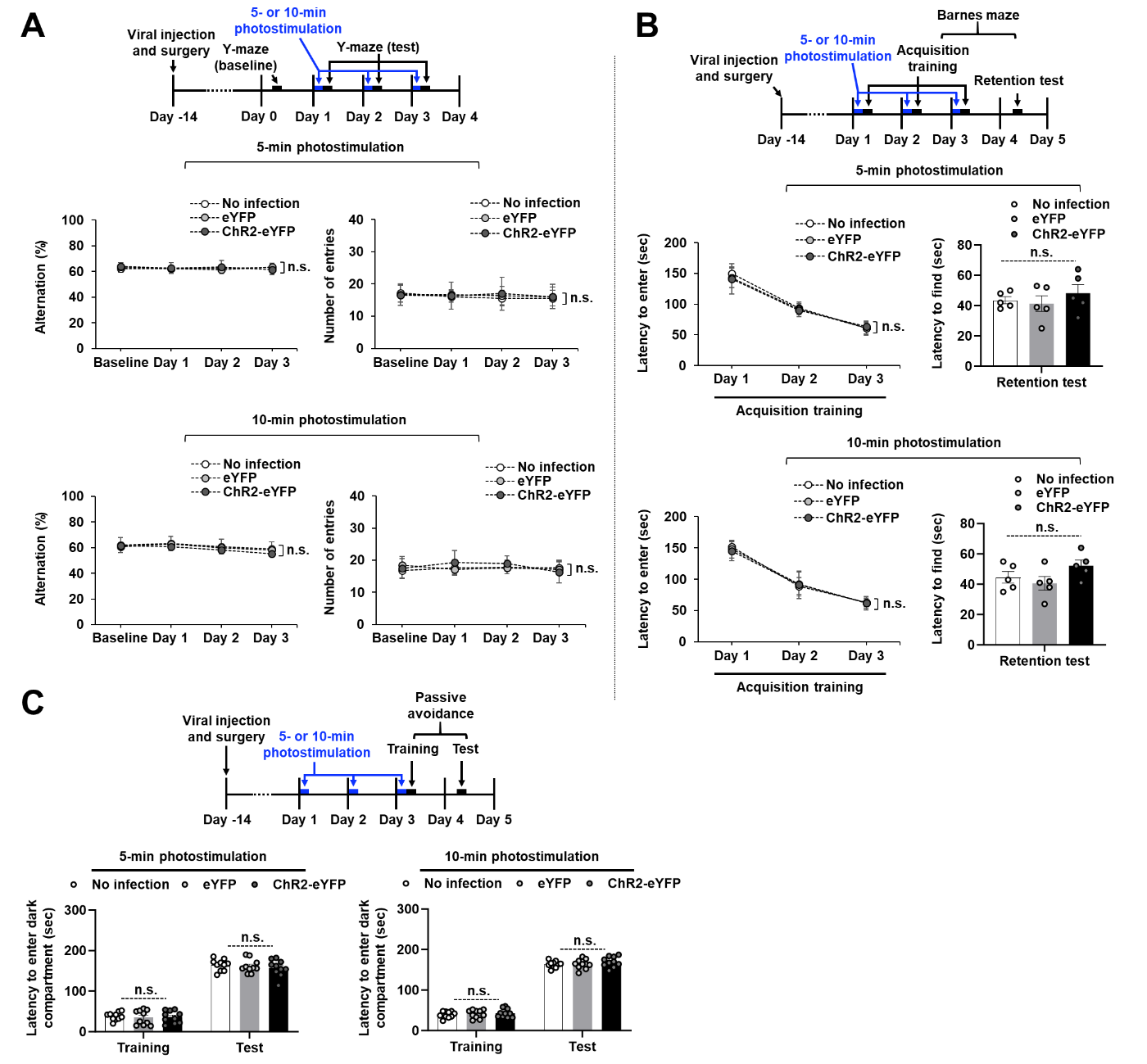

Supplement: S3 Fig — Experimental timeline of viral injection, surgery, optogenetic stimulation, and behavioral analysis. Mice were injected with AAV-GFAP-eYFP or AAV-GFAP-ChR2-eYFP. On day 14 post-injection and surgery, the first (day 1, in the timeline), second (day 2, in the timeline), and third (day 3, in the timeline) optogenetic stimulations were delivered for 5- or 10-min to the hippocampal CA1 region. Blue and black arrows indicate the time points for optogenetic stimulation and behavioral testing, respectively. The cognitive behavior of the eYFP or ChR2-eYFP expressing mice after optogenetic stimulation of the hippocampal astrocytes was analyzed by Y-maze (n = 10) (A), Barnes maze (n = 5) (B), and passive avoidance (n = 10) (C) tests. Results are expressed as mean ± SEM (n = 5 or 10). *p < 0.05 between the indicated groups; n.s., not significant (two-way ANOVA in A and B, one-way ANOVA in C). Source data can be found in S1 Data. (TIFF) [file pbio.3002687.s003.tiff]

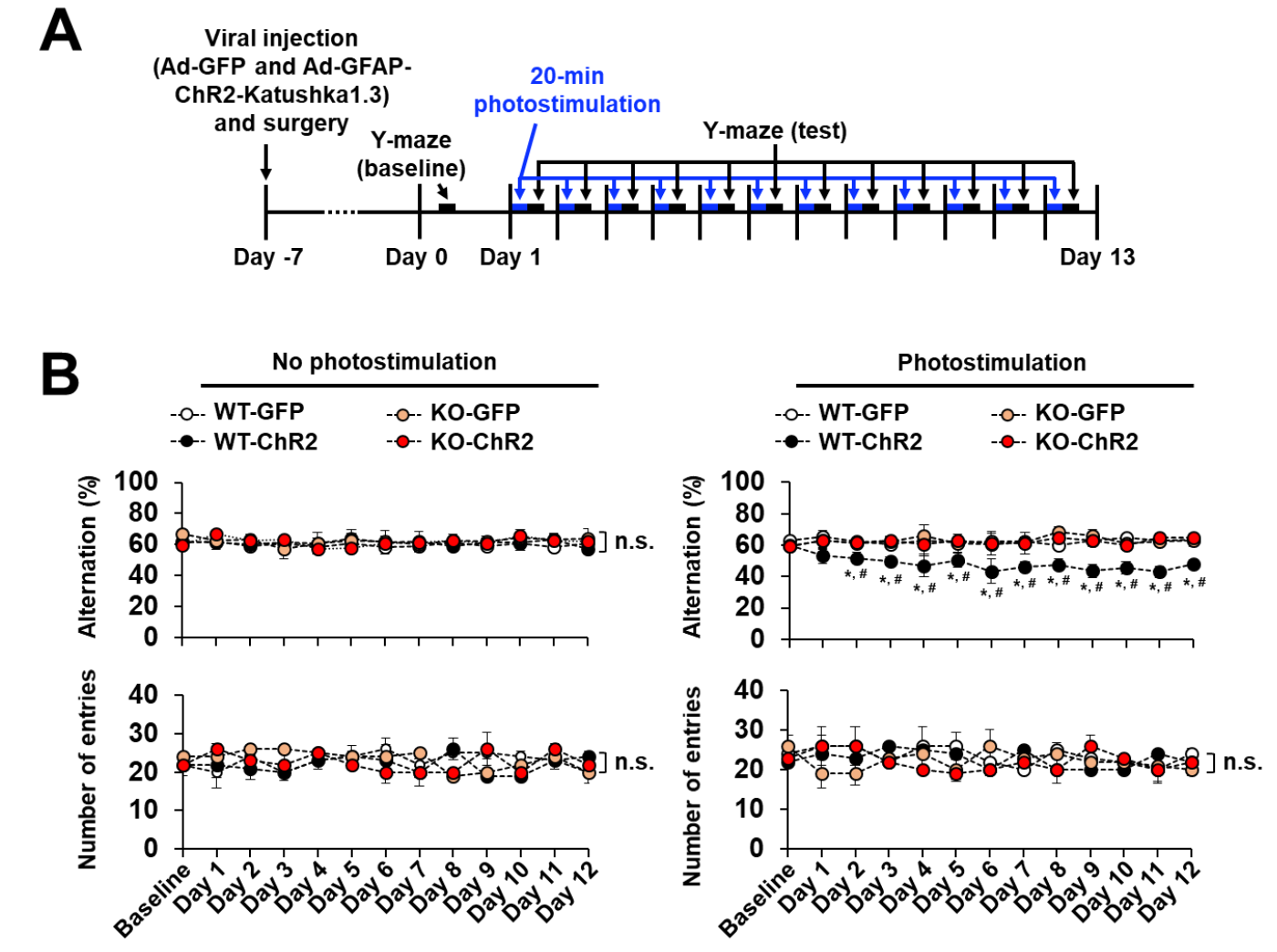

Supplement: S4 Fig — (A) Experimental timeline of viral injection, surgery, optogenetic stimulation, and behavioral analysis. Adenovirus expressing ChR2 under the control of astrocyte-specific GFAP promoter (Ad-GFAP-ChR2-Katushka1.3; Ad-ChR2) was used for the injection. The optic fibers and cannula were implanted above the hippocampal CA1 area for photostimulation. Seven days (day 1, in the timeline) after Ad-GFP or Ad-ChR2 viral injection, photostimulation was delivered for the duration of 20 min through the optogenetic fiber. Blue and black arrows indicate the time points of optogenetic stimulation and behavioral testing, respectively. (B) Ad-ChR2 injected WT mice (WT-ChR2) showed an apparent impairment of spatial memory compared with Ad-GFP-injected control animals (WT-GFP) through optogenetic stimulation. The optogenetic stimulation-induced spatial memory impairment was significantly attenuated in the Ad-ChR2 injected Lcn2-KO mice (Lcn2 KO-ChR2). There was no significant difference in the locomotor activity as determined by the number of entries. Results are expressed as mean ± SEM (n = 10). *p < 0.05, WT-GFP versus WT-ChR2; #p < 0.05, WT-ChR2 versus Lcn2 KO-ChR2; n.s., not significant (two-way ANOVA). Source data can be found in S1 Data. (TIFF) [file pbio.3002687.s004.tiff]

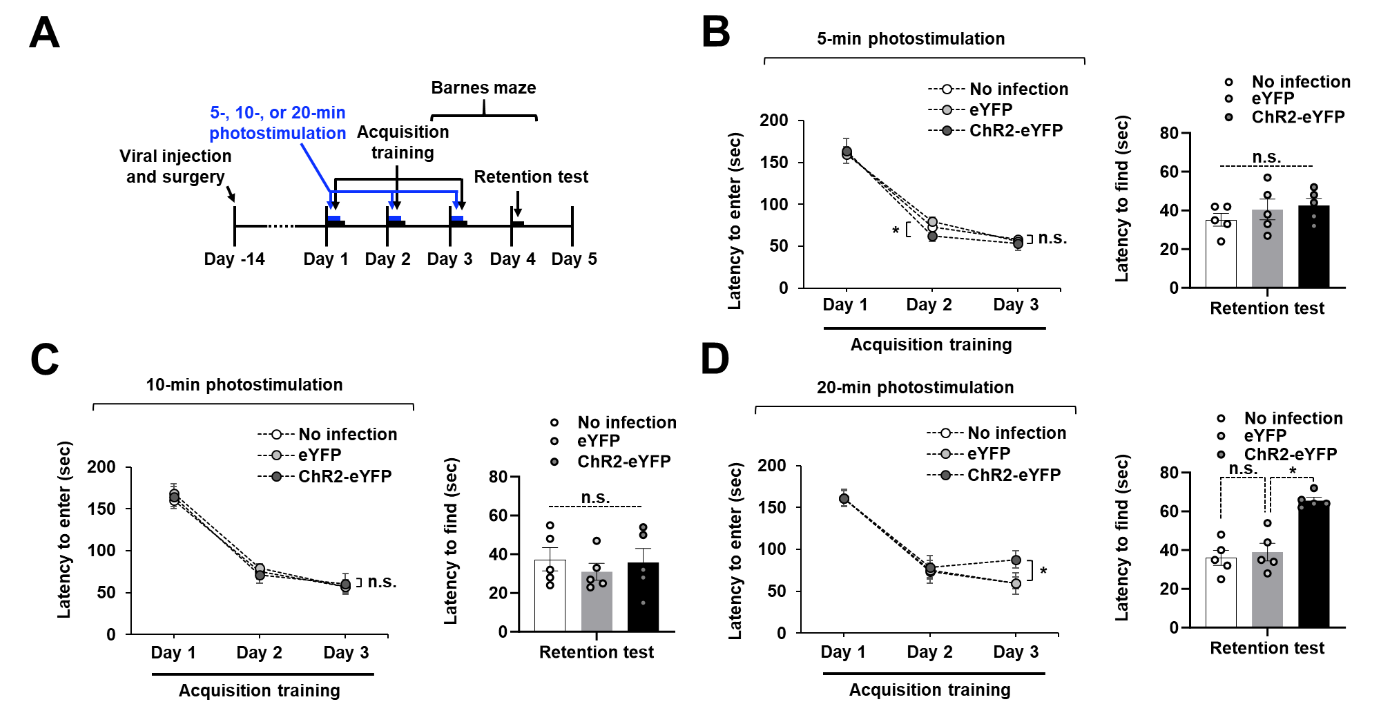

Supplement: S5 Fig — (A) Experimental timeline of viral injection, surgery, optogenetic stimulation, and behavioral analysis. Mice were injected with AAV-GFAP-eYFP or AAV-GFAP-ChR2-eYFP. On day 14 post-injection, optogenetic stimulations were delivered for 5-, 10-, or 20-min to the hippocampal CA1 region during the memory acquisition in the training phase. Blue and black lines indicate the time points for optogenetic stimulation and behavioral testing, respectively. (B–D) The cognitive behavior of eYFP or ChR2-eYFP expressing mice after the optogenetic stimulation of hippocampal astrocytes was analyzed by the Barnes maze test. Results are expressed as mean ± SEM (n = 5). *p < 0.05 between the indicated groups; n.s., not significant (two-way ANOVA). Source data can be found in S1 Data. (TIFF) [file pbio.3002687.s005.tiff]

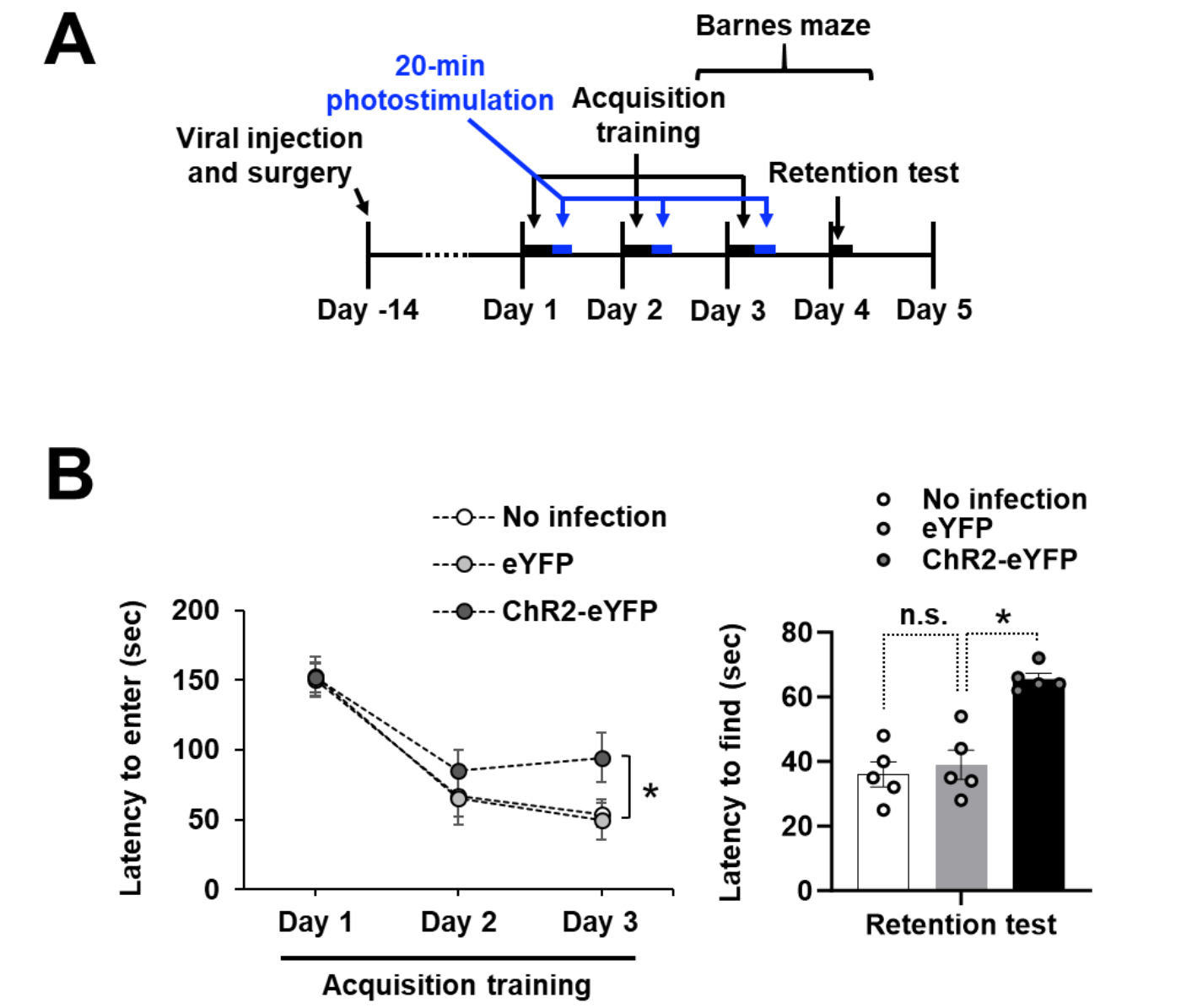

Supplement: S6 Fig — (A) Experimental timeline of viral injection, surgery, optogenetic stimulation, and behavioral analysis. Mice were injected with AAV-GFAP-eYFP or AAV-GFAP-ChR2-eYFP. On day 14 post-injection, optogenetic stimulations were delivered for 20 min to the hippocampal CA1 region after memory acquisition in the training phase. Blue and black lines indicate the time points of the optogenetic stimulation and behavioral testing, respectively. (B) The cognitive behavior of eYFP or ChR2-eYFP-expressing mice after the optogenetic stimulation of hippocampal astrocytes was analyzed by the Barnes maze test. Results are expressed as mean ± SEM (n = 5). *p < 0.05 between the indicated groups; n.s., not significant (two-way ANOVA). Source data can be found in S1 Data. (TIFF) [file pbio.3002687.s006.tiff]

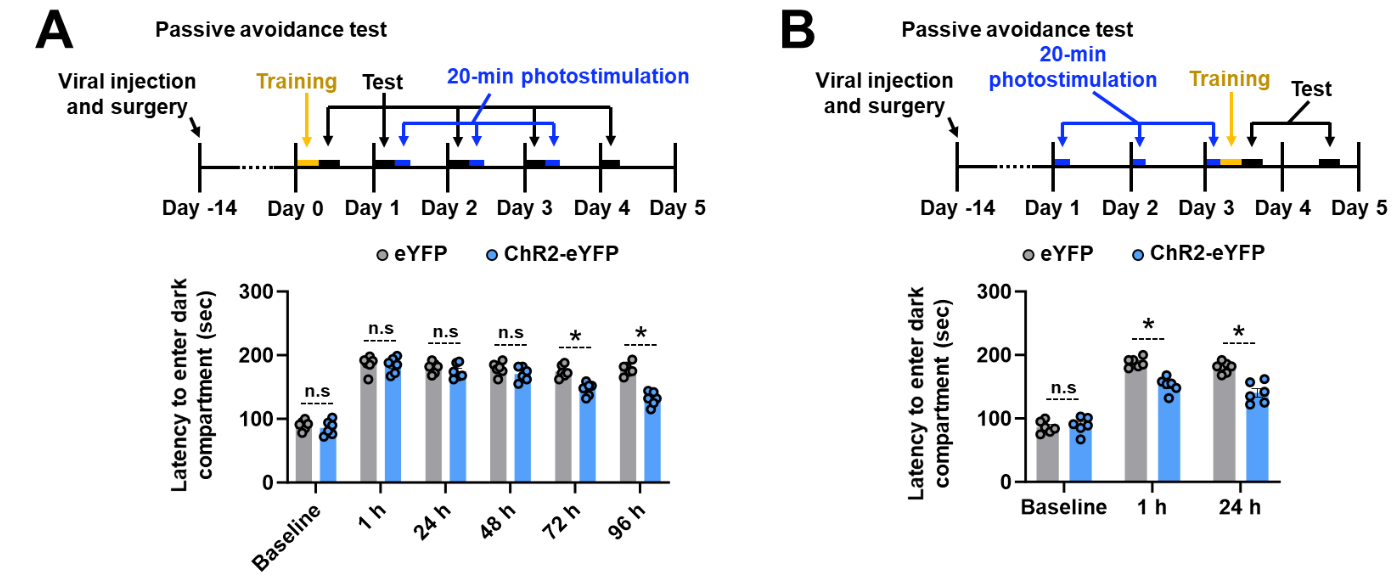

Supplement: S7 Fig — Experimental timeline of viral injection, surgery, optogenetic stimulation, and behavioral analysis (A and B, upper). Mice were injected with AAV-GFAP-eYFP or AAV-GFAP-ChR2-eYFP. On day 14 post-injection (day 1, in the timeline), the first (day 1, in the timeline), second (day 2, in the timeline), and third (day 3, in the timeline) optogenetic stimulations were delivered for 20 min to the hippocampal CA1 region, after (A) or before (B) the training stages. Blue and black arrows indicate the time points for the optogenetic stimulation and behavioral testing, respectively. The cognitive behavior of eYFP or ChR2-eYFP-expressing mice after the optogenetic stimulation of the hippocampal astrocytes was analyzed by passive avoidance (n = 6) (A and B, lower) tests. Results are expressed as mean ± standard deviation of the mean (SEM) (n = 6). *p < 0.05 between the indicated groups; n.s., not significant (one-way ANOVA). Source data can be found in S1 Data. (TIFF) [file pbio.3002687.s007.tiff]

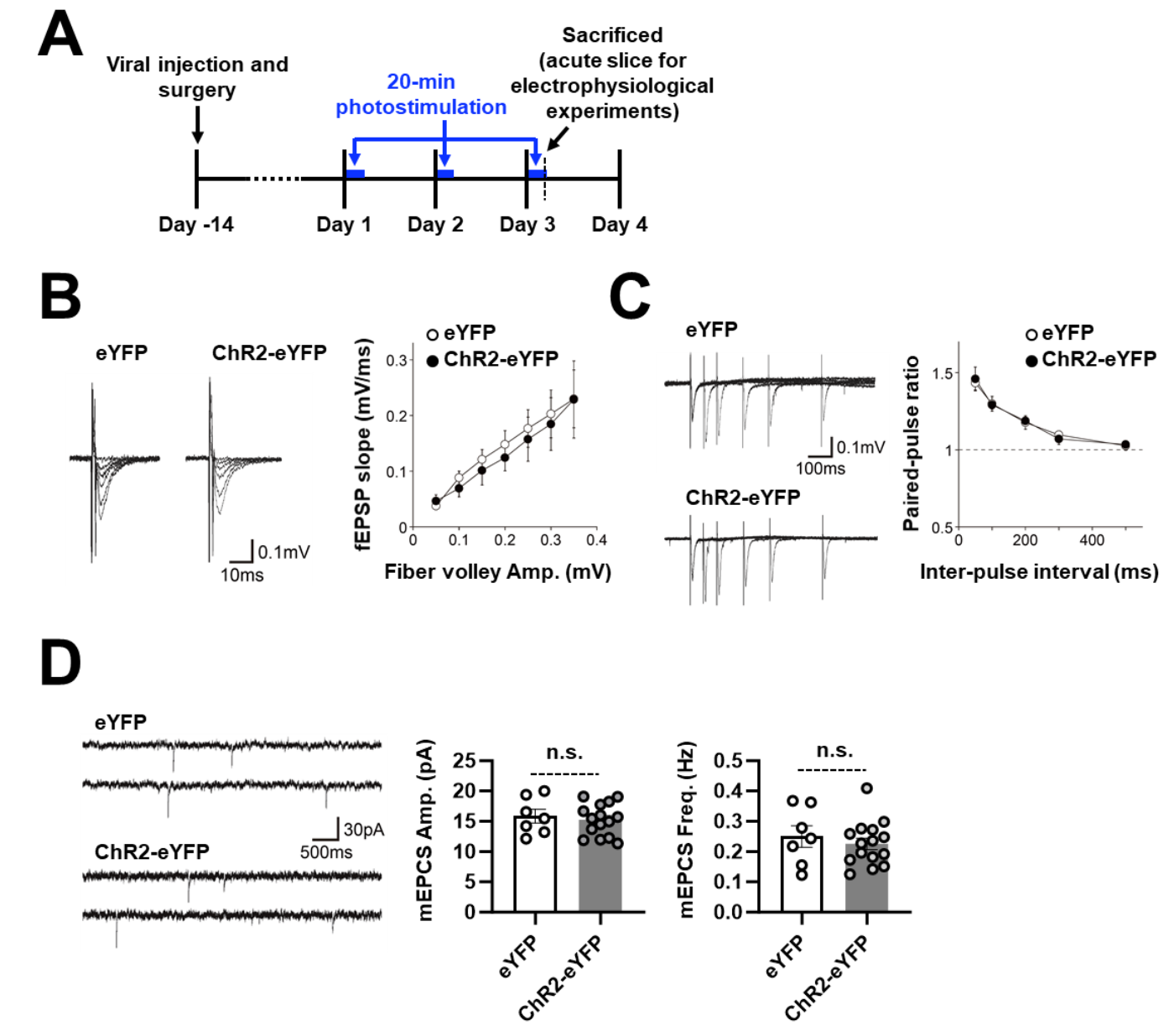

Supplement: S8 Fig — (A) Experimental timeline of viral injection, surgery, optogenetic stimulation, and electrophysiological experiments. (B) Typical traces of the fEPSPs evoked by various strengths of stimuli in the eYFP and ChR2-eYFP expressing groups (left). The relationship between the fiber volley amplitude (input) and fEPSP amplitude (output) in the eYFP (open circles, n = 4) and ChR2-eYFP expressing groups (closed circles, n = 4) (right). To examine the input–output relationship, the fEPSPs were recorded in the presence of 3 μm CNQX to reduce the fEPSP amplitude. (C) Typical traces of the fEPSPs evoked by paired-pulse stimuli with various intervals in the eYFP (left upper) and ChR2-eYFP expressing groups (left lower). The paired-pulse ratio of the fEPSPs in the eYFP (open circles, n = 4) and ChR2-eYFP expressing groups (closed circles, n = 4) (right). (D) Typical traces of the mEPSCs recorded from the hippocampal CA1 neurons in the eYFP (left upper) and ChR2-eYFP expressing groups (left lower). The mean amplitude (middle) and frequency (right) of the mEPSCs in the eYFP (n = 7) and ChR2-eYFP expressing groups (n = 15). Results are expressed as mean ± SEM. n.s., not significant (unpaired t test). Source data can be found in S1 Data. (TIFF) [file pbio.3002687.s008.tiff]

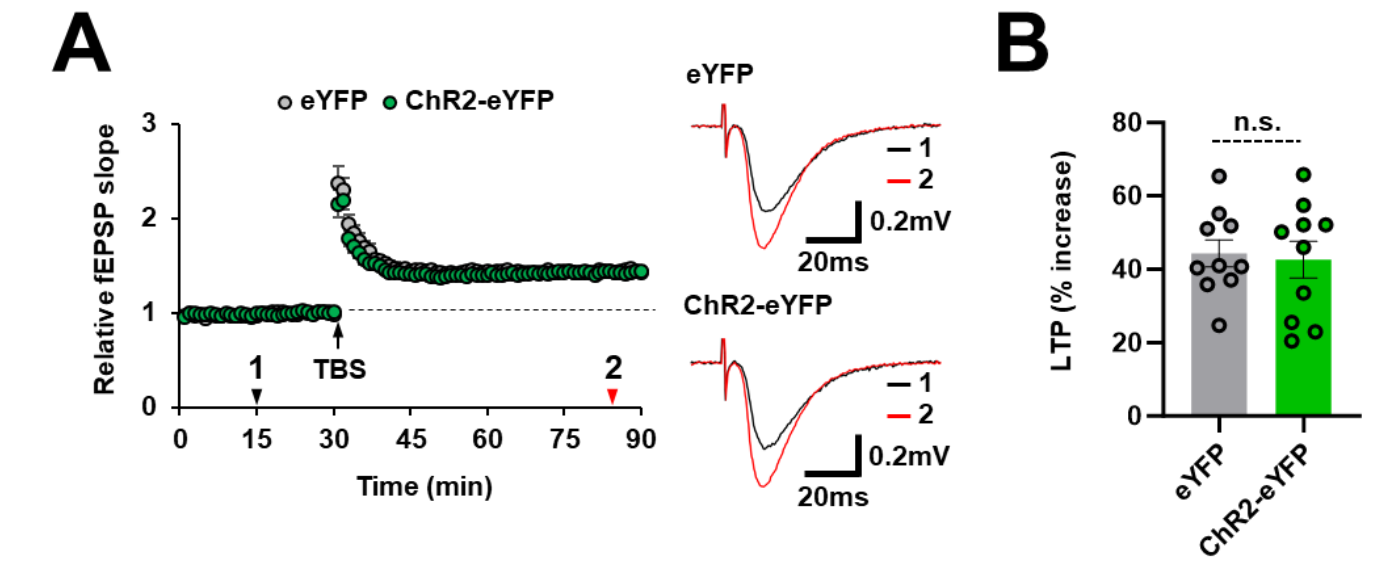

Supplement: S9 Fig — (A) Time courses of the fEPSP responses before and after TBS from the hippocampal slices in the eYFP (gray circles, n = 10 from 5 mice) and ChR2-eYFP groups (green circles, n = 10 from 5 mice). In these experiments, photostimulation was applied for 5 min. TBS was applied for LTP induction at 30 min. Insets represent the typical raw traces from the average of 6 successive fEPSPs recorded at the time indicated by arrowheads with numbered regions (1; black or 2; red). (B) TBS-induced LTP in eYFP (n = 10 from 5 mice) and ChR2-eYFP (n = 10 from 5 mice) groups. The mean fEPSP slope during the 50–60 min after TBS was quantified as the LTP level. Results are expressed as mean ± SEM. n.s., not significant (unpaired t test). Source data can be found in S1 Data. (TIFF) [file pbio.3002687.s009.tiff]

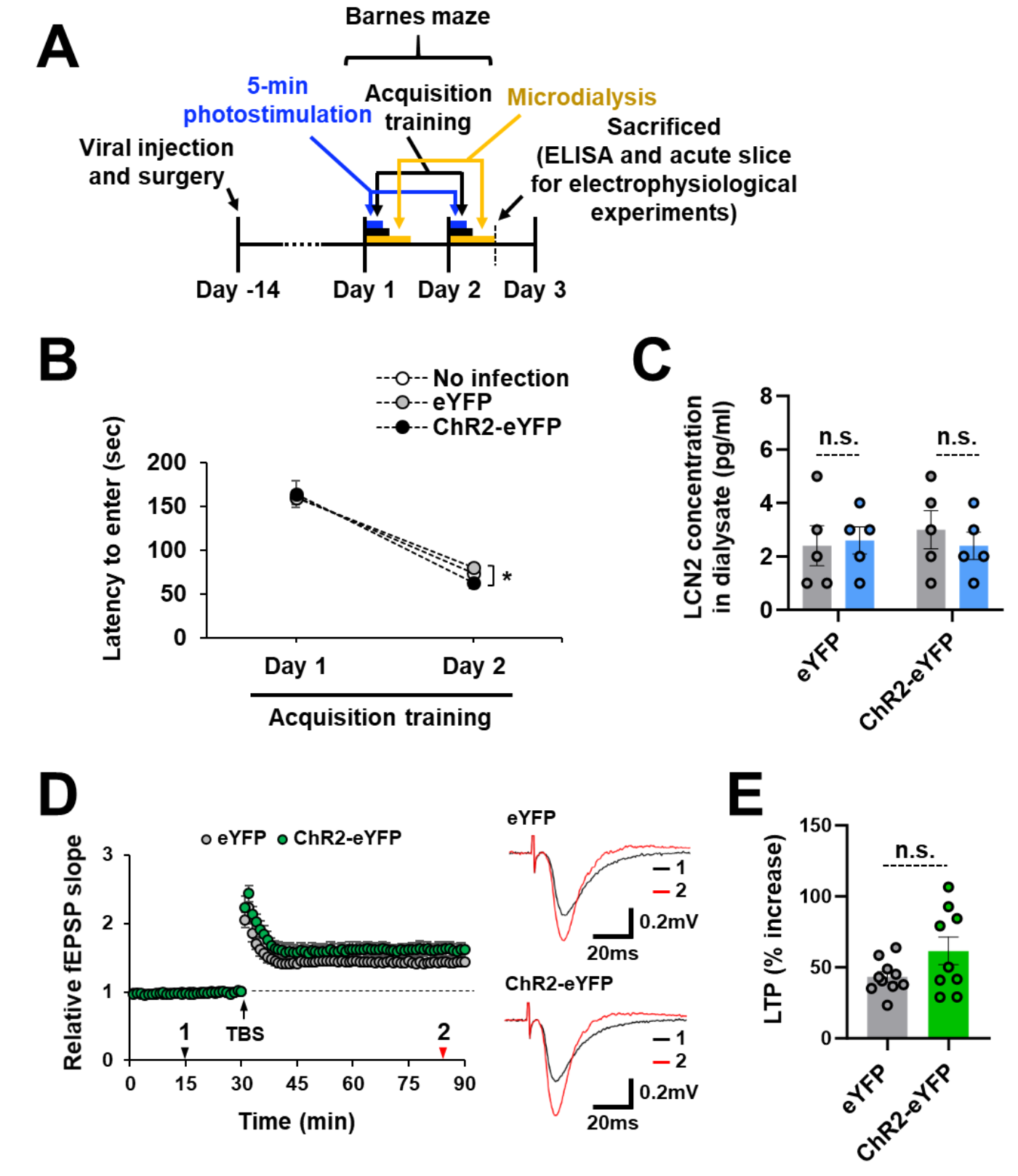

Supplement: S10 Fig — (A) Experimental timeline of the viral injection, surgery, optogenetic stimulation, and behavioral analysis. Mice were injected with AAV-GFAP-eYFP or AAV-GFAP-ChR2-eYFP. On day 14 post-injection, optogenetic stimulations were delivered for 5 min to the hippocampal CA1 region during memory acquisition in the training phase. Blue, black, and yellow lines indicate the time points of optogenetic stimulation, behavioral testing, and microdialysis, respectively. (B) Cognitive behavior of the eYFP or ChR2-eYFP-expressing mice groups after optogenetic stimulation of the hippocampal astrocytes was analyzed by the Barnes maze test. Results are expressed as mean ± SEM (n = 4). *p < 0.05, eYFP versus ChR2-eYFP groups; n.s., not significant (two-way ANOVA). (C) The 5-min photostimulation during memory acquisition of hippocampal CA1 astrocytes did not significantly affect the extracellular LCN2 release at day 2. The LCN2 levels in the dialysate were measured by ELISA. Results are expressed as mean ± SEM (n = 5). n.s., not significant (one-way ANOVA). (D) Time courses of the fEPSP responses before and after the TBS from the hippocampal sections in the eYFP (gray, n = 10 from 5 mice) and ChR2-eYFP (green, n = 10 from 5 mice) groups. In these experiments, photostimulation was applied for 5 min before behavioral training. TBS was applied for LTP induction at 30 min. Insets represent the typical raw traces from the average of 6 successive fEPSPs recorded at the time indicated by the arrowheads with numbered regions (1; black or 2; red). (E) TBS-induced LTP in the eYFP (n = 10 from 5 mice) and ChR2-eYFP (n = 9 from 5 mice) groups. The mean fEPSP slope during the 50 to 60 min after TBS was quantified as the LTP level. Results are expressed as mean ± SEM. n.s., not significant (unpaired t test). Source data can be found in S1 Data. (TIFF) [file pbio.3002687.s010.tiff]

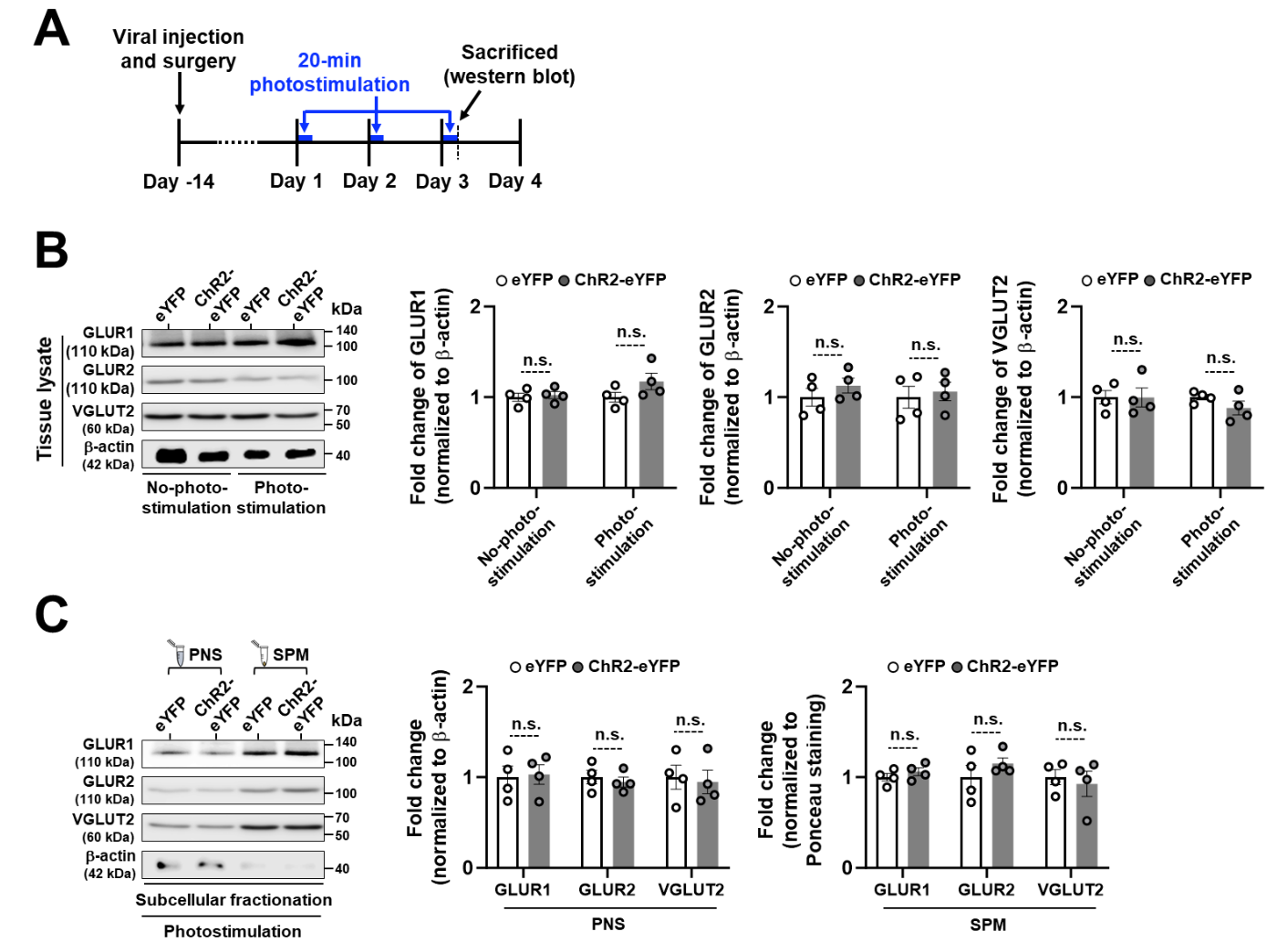

Supplement: S11 Fig — (A) Experimental timeline of the viral injection, surgery, optogenetic stimulation, and behavioral analysis. (B) AMPA receptors (GLUR1 and GLUR2) and vesicular glutamate transporter 2 (VGLUT2) protein levels were measured by western blotting. Quantification of the band intensities is presented in the adjacent graphs. Results are expressed as mean ± SD (n = 4). n.s., not significant (one-way ANOVA). (C) Western blot analysis of GLUR1, GLUR2, and VGLUT2 present in the subcellular fractions (PNS and SPM) obtained from the hippocampal tissues of the eYFP and ChR2-eYFP expressing mice. Quantification of GLUR1, GLUR2, or VGLUT2 in the PNS was based on normalization against the internal control β-actin and that of GLUR1, GLUR2, or VGLUT2 in the SPM was normalized against the internal control Ponceau S (S13B Fig) and are represented as graphs for the blots. Results are expressed as mean ± SD (n = 4). n.s., not significant (one-way ANOVA). Source data can be found in S1 Data. (TIFF) [file pbio.3002687.s011.tiff]

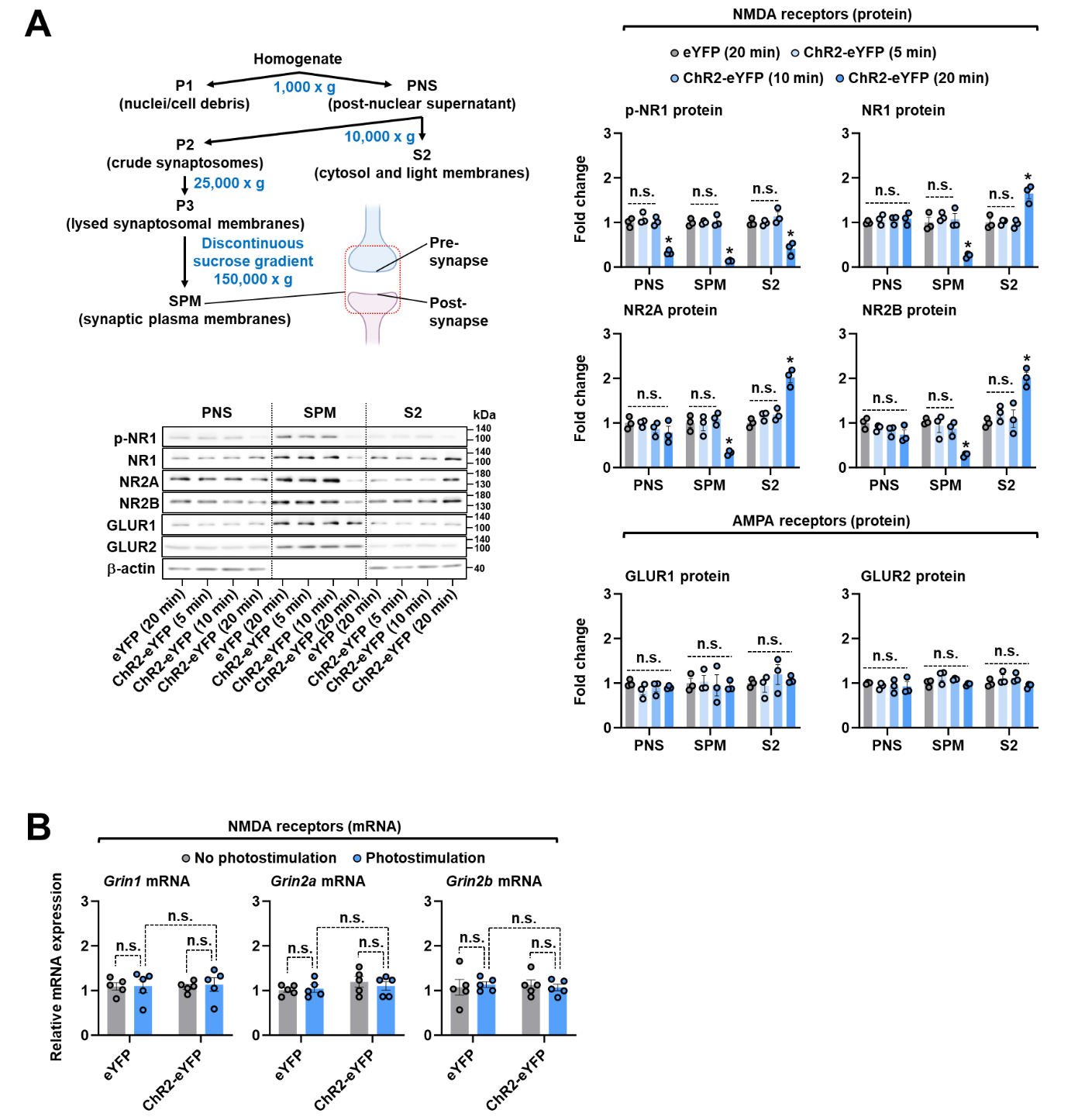

Supplement: S12 Fig — (A) Schematic depiction of the centrifugation steps to produce the PNS, SPM, and S2 from hippocampal tissue samples. Western blot analysis of p-NR1, NR1, NR2A, NR2B, GLUR1, and GLUR2 present in each subcellular fraction (PNS, SPM, and S2) obtained from the hippocampal tissues of eYFP- or ChR2-eYFP-expressing mice (at 5-, 10-, and 20-min photostimulation). Quantification of p-NR1, NR1, NR2A, or NR2B in the PNS and S2 was based on the normalization against the internal control β-actin, and that for p-NR1, NR1, NR2A, NR2B, GLUR1, or GLUR2 in the SPM was normalized against the internal control Ponceau S and represented as graphs for the blots. The Ponceau S-stained membranes are shown in S13E Fig. Results are expressed as mean ± SD (n = 3). *p < 0.05 versus eYFP (20 min) control groups; n.s., not significant (one-way ANOVA). (B) mRNA levels of NMDA receptors (Grin1, Grin2a, and Grin2b) were measured by qPCR. Results are expressed as mean ± SD (n = 5). n.s., not significant (one-way ANOVA). The SPM contains cell surface membranes, and S2 contains intracellular organelle membranes and cytosol. Source data can be found in S1 Data. (TIFF) [file pbio.3002687.s012.tiff]

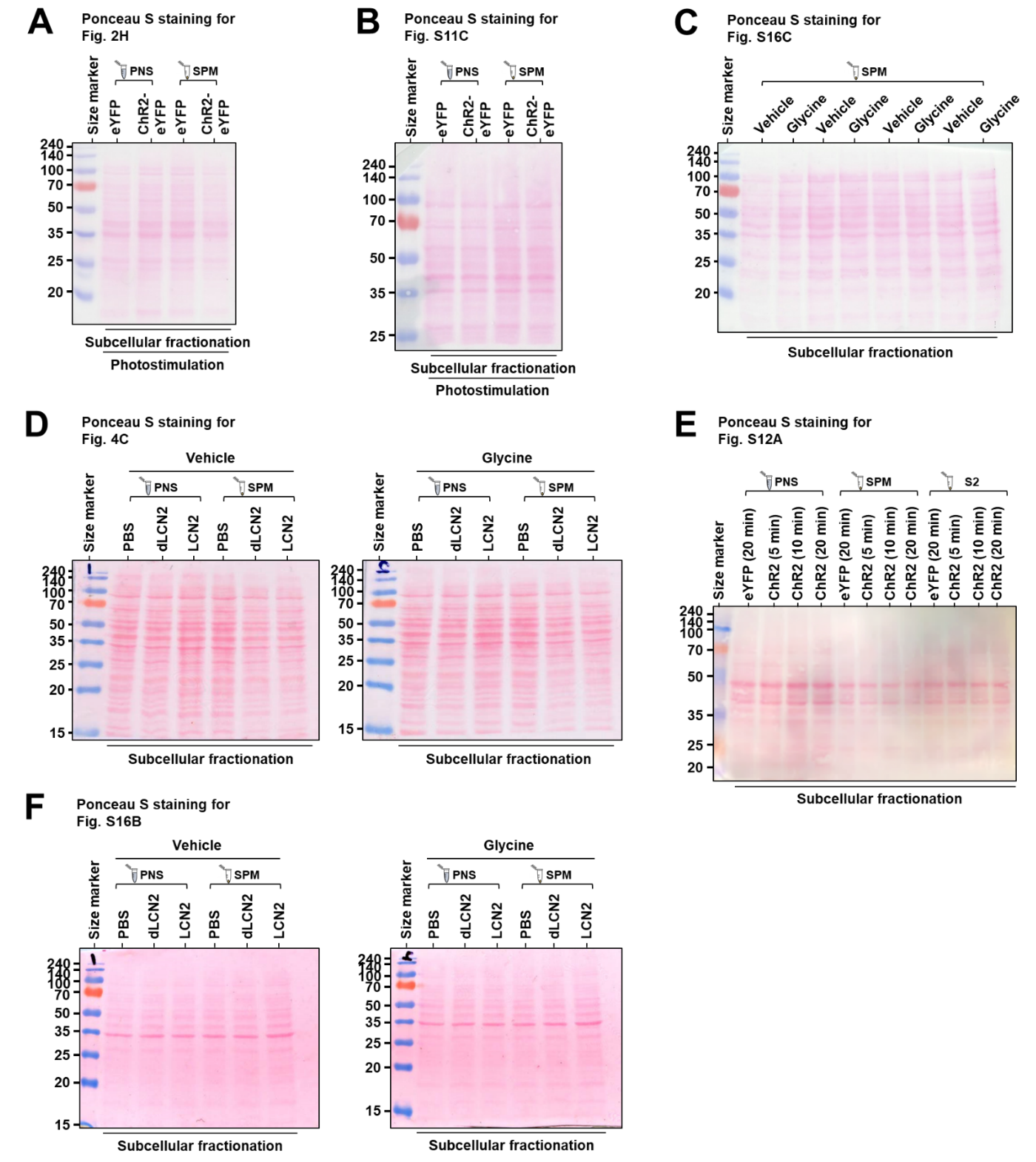

Supplement: S13 Fig — The same amounts of PNS, SPM, and S2 from the hippocampal tissue (A, B, and E) or cultured hippocampal neurons (C, D, and F) were resolved by SDS-PAGE, as shown on Ponceau S-stained blots. Quantification of each protein in the SPM was normalized to Ponceau S and represented as graphs in each figure indicated. (A) Ponceau S staining of Fig 2H. (B) Ponceau S staining of S11C Fig. (C) Ponceau S staining of S16C Fig. (D) Ponceau S staining of Fig 4C. (E) Ponceau S staining of S12A Fig, (F) and Ponceau S staining of S16B Fig. (TIFF) [file pbio.3002687.s013.tiff]

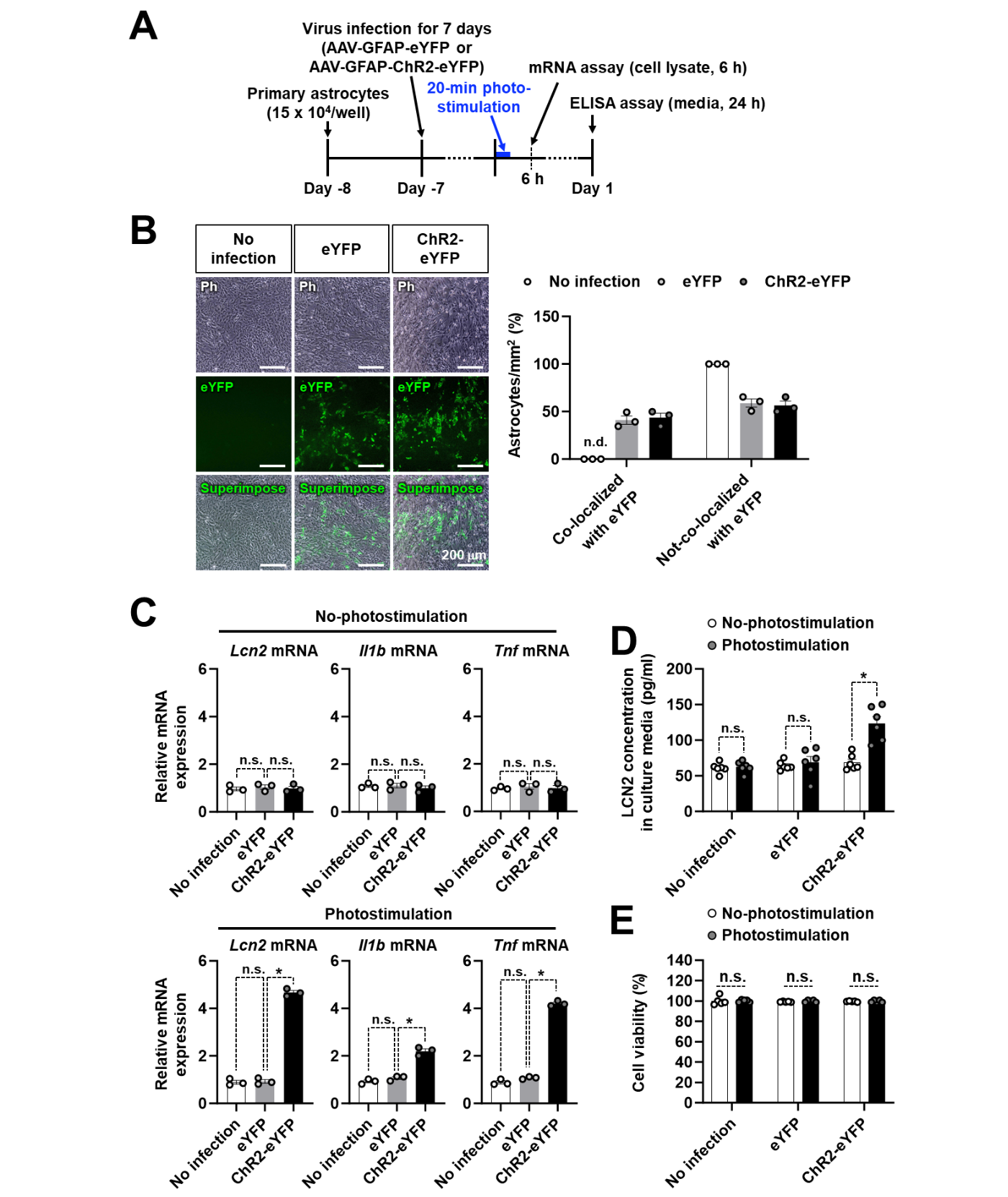

Supplement: S14 Fig — (A) Experimental timeline of viral injection, surgery, optogenetic stimulation, and behavioral analysis. (B) Phase contrast (Ph) and fluorescence images of astrocytes in the primary cultures infected with AAV-GFAP-eYFP (green) or AAV-GFAP-ChR2-eYFP (green). The number of cells and their co-localization (%) are shown. Quantification of the eYFP-positive cell colocalization is shown in the adjacent graphs. Scale bar: 200 μm. Results are expressed as mean ± SEM (n = 3). n.d., not detected. (C) Primary astrocyte cultures were illuminated using the LED device for 20 min, and total RNA was extracted after 6 h. The mRNA levels of Lcn2, Il1b, and Tnf were determined by qPCR (upper, no photostimulation; lower, photostimulation). Data were normalized to the internal control Gapdh, and results are expressed as mean ± SD (n = 3) in the graphs. *p < 0.05 between the indicated groups; n.s., not significant (one-way ANOVA). (D) The levels of LCN2 protein secretion in the culture media were measured by ELISA at 24 h after photostimulation. Results are expressed as mean ± SEM (n = 6). *p < 0.05 between the indicated groups; n.s., not significant (one-way ANOVA). (E) Cell viability was measured using an MTT assay at 24 h after the photostimulation. Results are expressed as mean ± SEM (n = 5). *p < 0.05 between the indicated groups; n.s., not significant (one-way ANOVA). Source data can be found in S1 Data. (TIFF) [file pbio.3002687.s014.tiff]

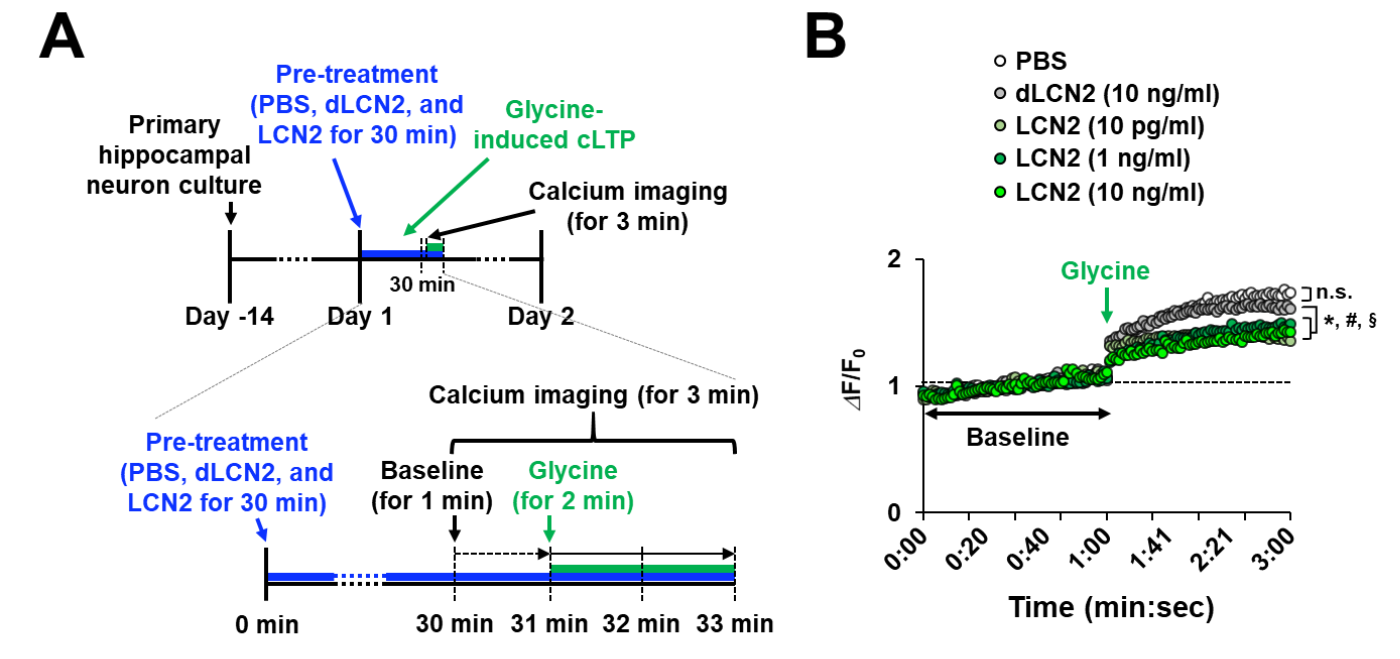

Supplement: S15 Fig — (A) Experimental timeline of viral injection, surgery, optogenetic stimulation, and behavioral analysis. (B) Fluo-4 AM-loaded neurons were stimulated with glycine (200 μm) (for each group in the presence of PBS), denatured LCN2 (dLCN2, 10 ng/ml), and LCN2 (10 pg/ml, 1 ng/ml, and 10 ng/ml), and Ca2+ transient was analyzed by Lionheart FX automated imaging analyzer. Results are expressed as mean ± SD (n = 4 or 7). n.s., not significant, PBS versus dLCN2; *p < 0.05, dLCN2 versus LCN2 (10 pg/mL); #p < 0.05, dLCN2 versus LCN2 (1 ng/ml); §p < 0.05, dLCN2 versus LCN2 (1 ng/ml) (one-way ANOVA). Source data can be found in S1 Data. (TIFF) [file pbio.3002687.s015.tiff]

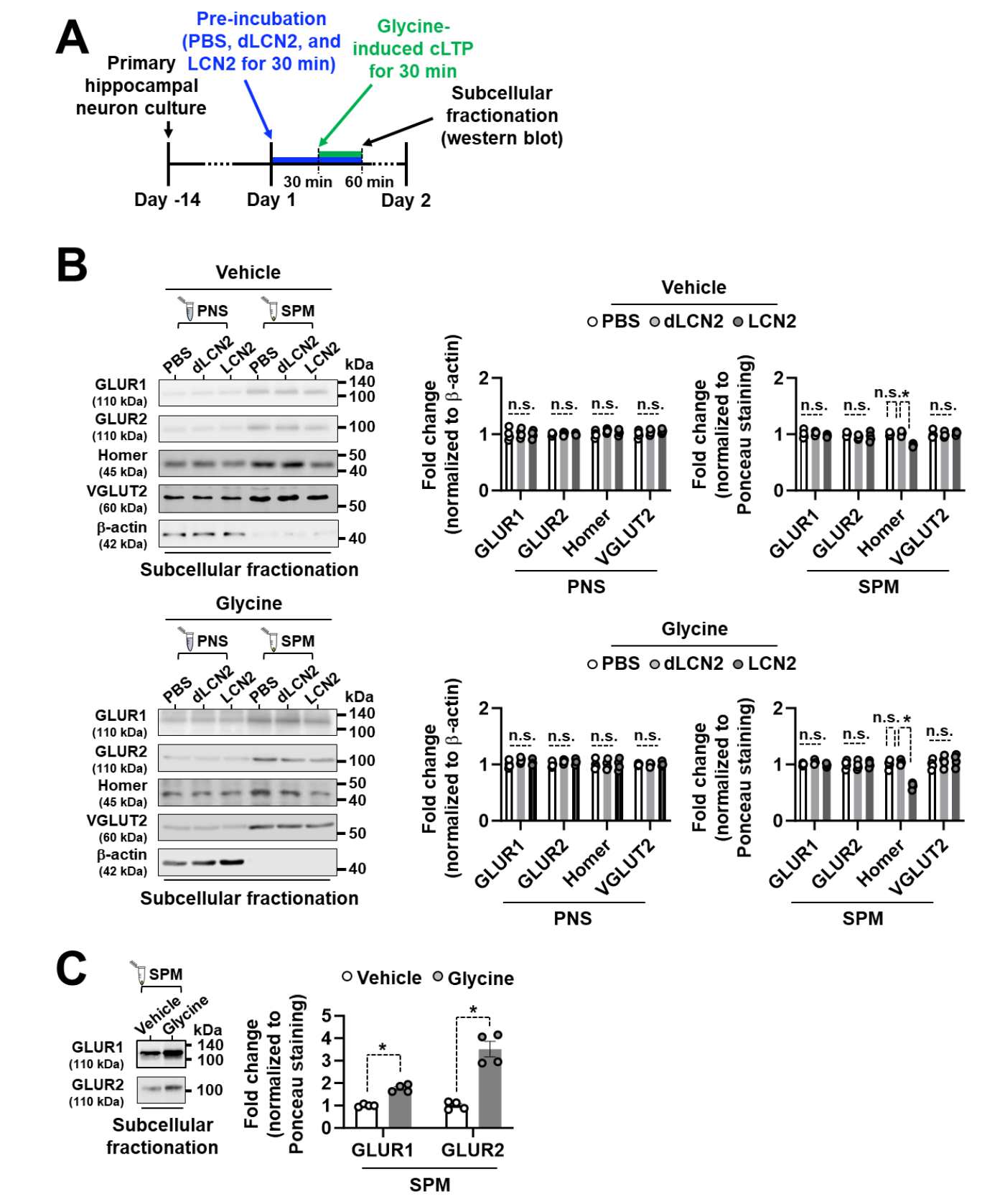

Supplement: S16 Fig — (A) Experimental timeline for cultured hippocampal neurons were stimulated with vehicle or glycine (200 μm) after incubation with PBS, denatured LCN2 (dLCN2, 10 ng/ml), or LCN2 (1 ng/ml) protein. (B) Western blot analysis of GLUR1, GLUR2, Homer, or VGLUT2 present in PNS and SPM obtained from the hippocampal neurons under each condition. Quantification of GluR1, GluR2, Homer, or VGLUT2 in PNS was based on normalization against the internal control β-actin, and that of GLUR1, GLUR2, Homer, or VGLUT2 in SPM was normalized against the internal control Ponceau S (S13C Fig) and represented as graphs for the blots. Results are expressed as mean ± SD (n = 4). *p < 0.05 between the indicated groups; n.s., not significant (one-way ANOVA). (C) Western blot analysis of GLUR1 or GLUR2 present in SPM obtained from the hippocampal neurons after glycine treatment. Quantification of GLUR1 or GLUR2 in SPM was normalized against the internal control Ponceau S (S13F Fig) and represented as graphs for the blots. Results are expressed as mean ± SD (n = 4). *p < 0.05 between the indicated groups (one-way ANOVA). Source data can be found in S1 Data. (TIFF) [file pbio.3002687.s016.tiff]

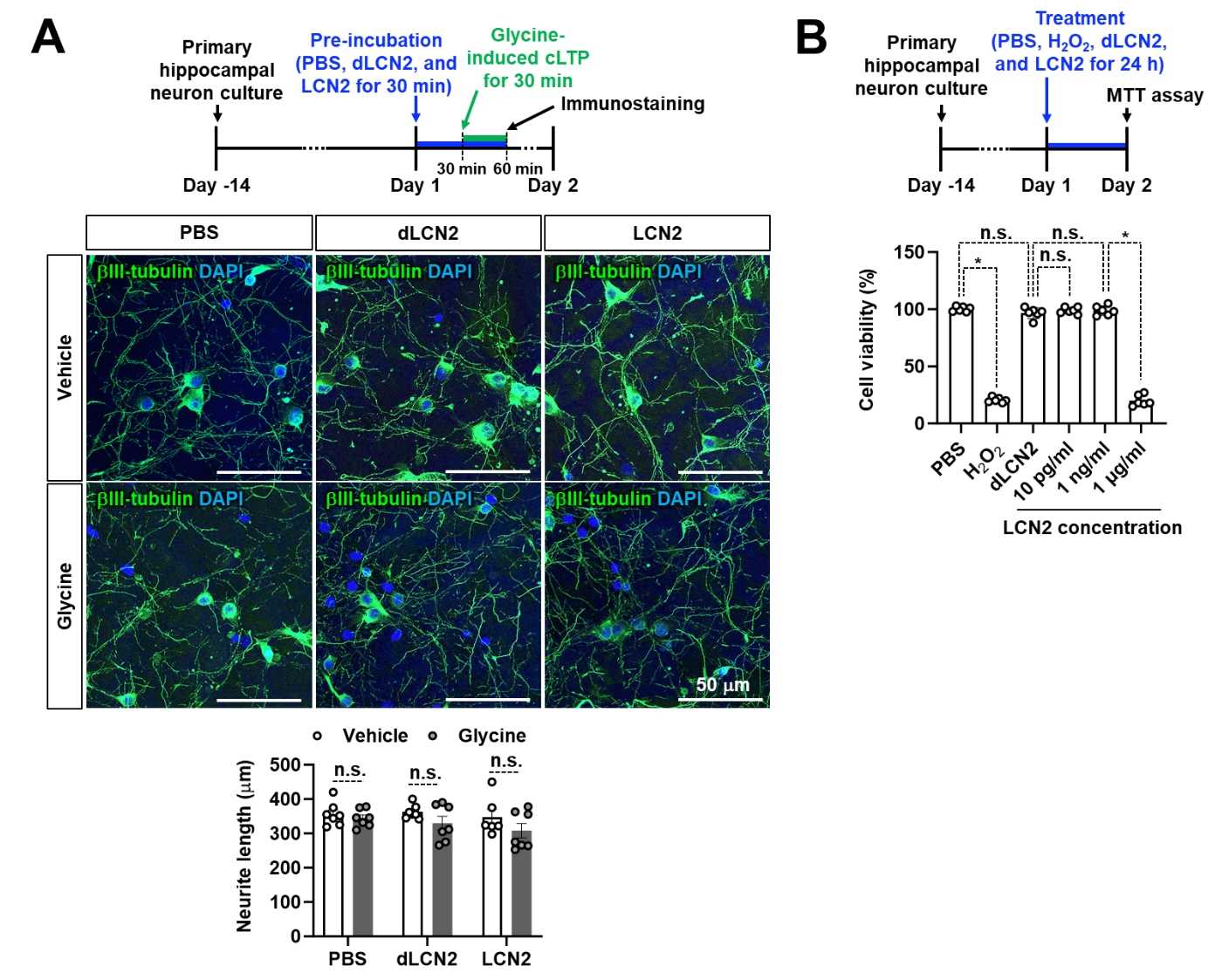

Supplement: S17 Fig — (A) Experimental timeline. Hippocampal neurons in media with or without glycine (200 μm), in the presence of PBS, denatured LCN2 (dLCN2, 10 ng/ml), or LCN2 (1 ng/ml) protein. Scale bar: 50 μm. Mean neurite length was determined from 6–7 cells under each culture condition obtained from 3 separate experiments. LCN2 (1 ng/ml) had no effect on neurite length in the hippocampal neurons. Results are expressed as mean ± SD (n = 6 or 7). n.s., not significant, between the indicated groups (one-way ANOVA). (B) No significant effect of LCN2 (10 pg/ml and 1 ng/ml) on cell viability. Hydrogen peroxide (H2O2, 500 μm) or LCN2 (1 μg/ml) was used as a positive control. Results are expressed as mean ± SD (n = 6). *p < 0.05 between the indicated groups; n.s., not significant (one-way ANOVA). Source data can be found in S1 Data. (TIFF) [file pbio.3002687.s017.tiff]

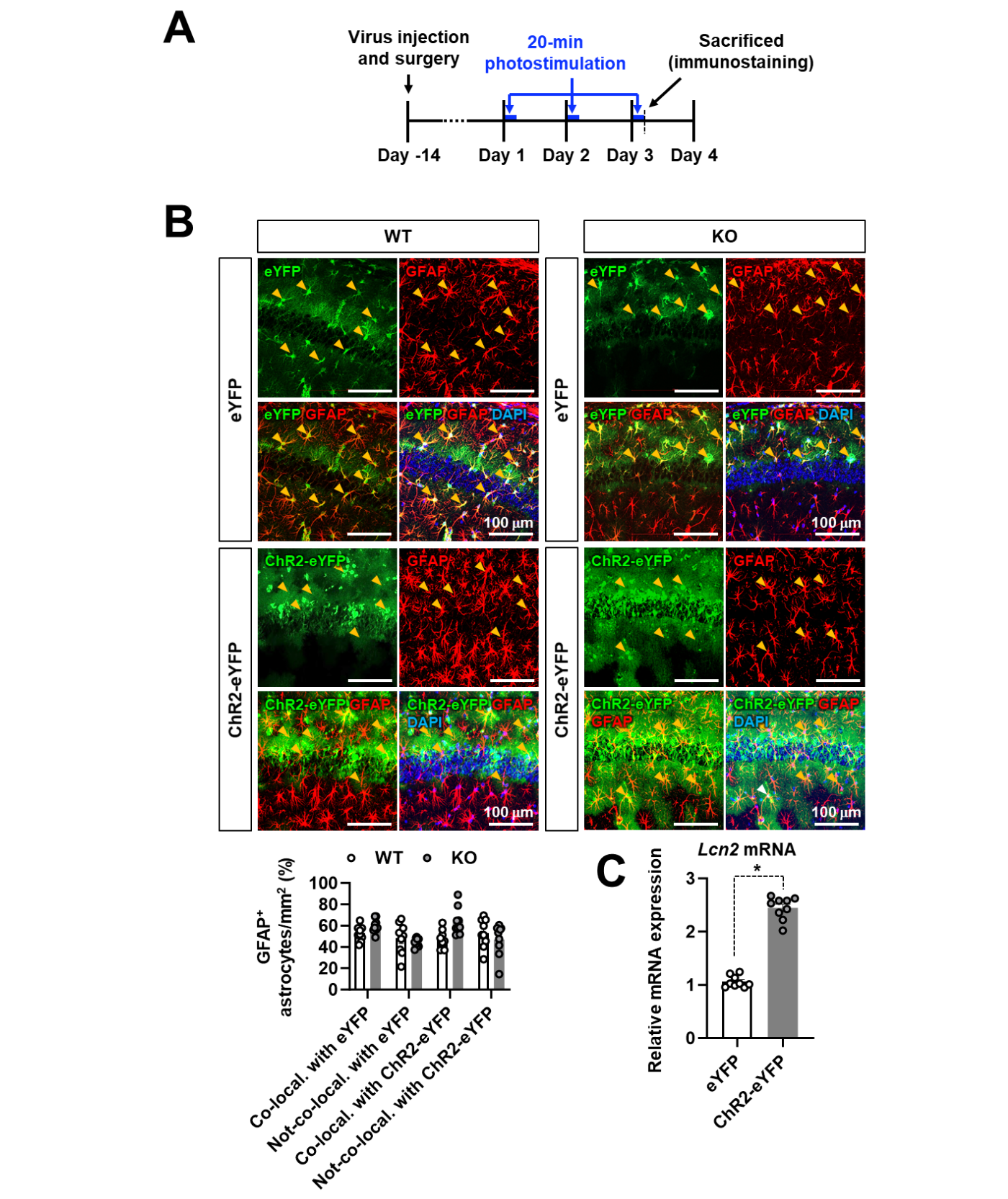

Supplement: S18 Fig — (A) Experimental timeline of viral injection, surgery, optogenetic stimulation, and behavioral analysis. (B) Brain tissue samples from the WT and Lcn2-KO mice were subjected to immunofluorescence analysis to localize the expression of the eYFP (green) and ChR2-eYFP (green) in astrocytes (GFAP, red). The nuclei were stained with DAPI (blue). Arrowheads (yellow) indicate the colocalization of eYFP, ChR2-eYFP, and GFAP. Quantification of the eYFP, ChR2-eYFP, and GFAP colocalization is shown in the adjacent graphs. Scale bar: 100 μm. Results are expressed as mean ± SEM (n = 9). (C) Total mRNA was extracted from the hippocampal tissue of each group after photostimulation in the eYFP and ChR2-eYFP expressing WT mice and subjected to qPCR to evaluate the expression levels of Lcn2. Gapdh was used as an internal control. Results are expressed as mean ± SEM (n = 9). *p < 0.05 between the indicated groups (Student’s t test). Source data can be found in S1 Data. (TIFF) [file pbio.3002687.s018.tiff]

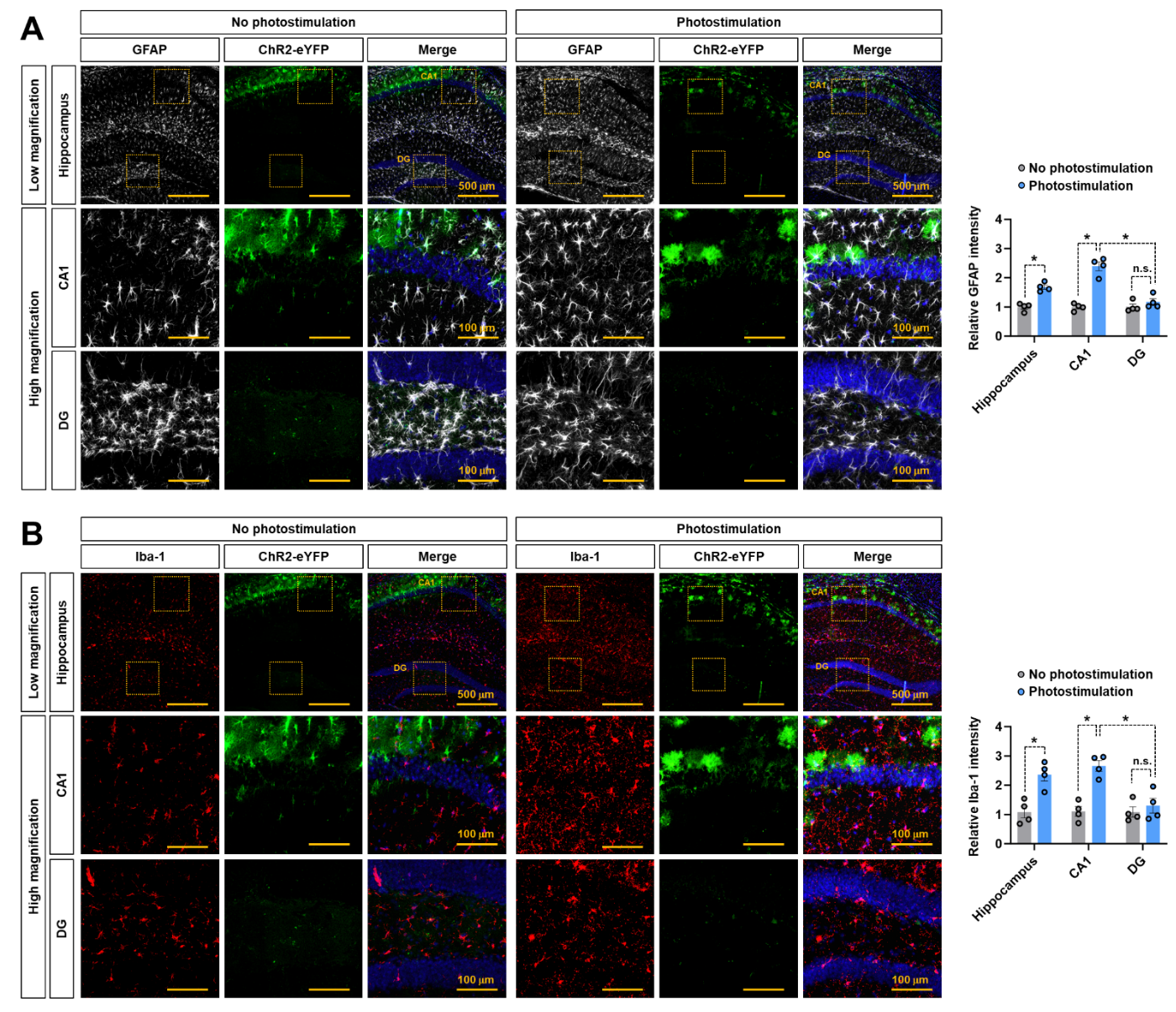

Supplement: S19 Fig — Brain tissue samples were subjected to immunofluorescence analysis to localize the expression of the ChR2-eYFP (green) in astrocytes (GFAP, red, A) and microglia (Iba-1, red, B). The nuclei were stained with DAPI (blue). Arrowheads (yellow) indicate the colocalization of ChR2-eYFP and GFAP-positive astrocytes. The adjacent graph displays the quantification of fluorescence intensity (astrocytes, A; microglia, B) in the whole hippocampus, CA1, or DG region. Scale bar: 500 μm (low magnification), and 100 μm (high magnification). Results are expressed as mean ± SEM (n = 4). *p < 0.05 between the indicated groups; n.s., not significant (one-way ANOVA). Source data can be found in S1 Data. (TIFF) [file pbio.3002687.s019.tiff]

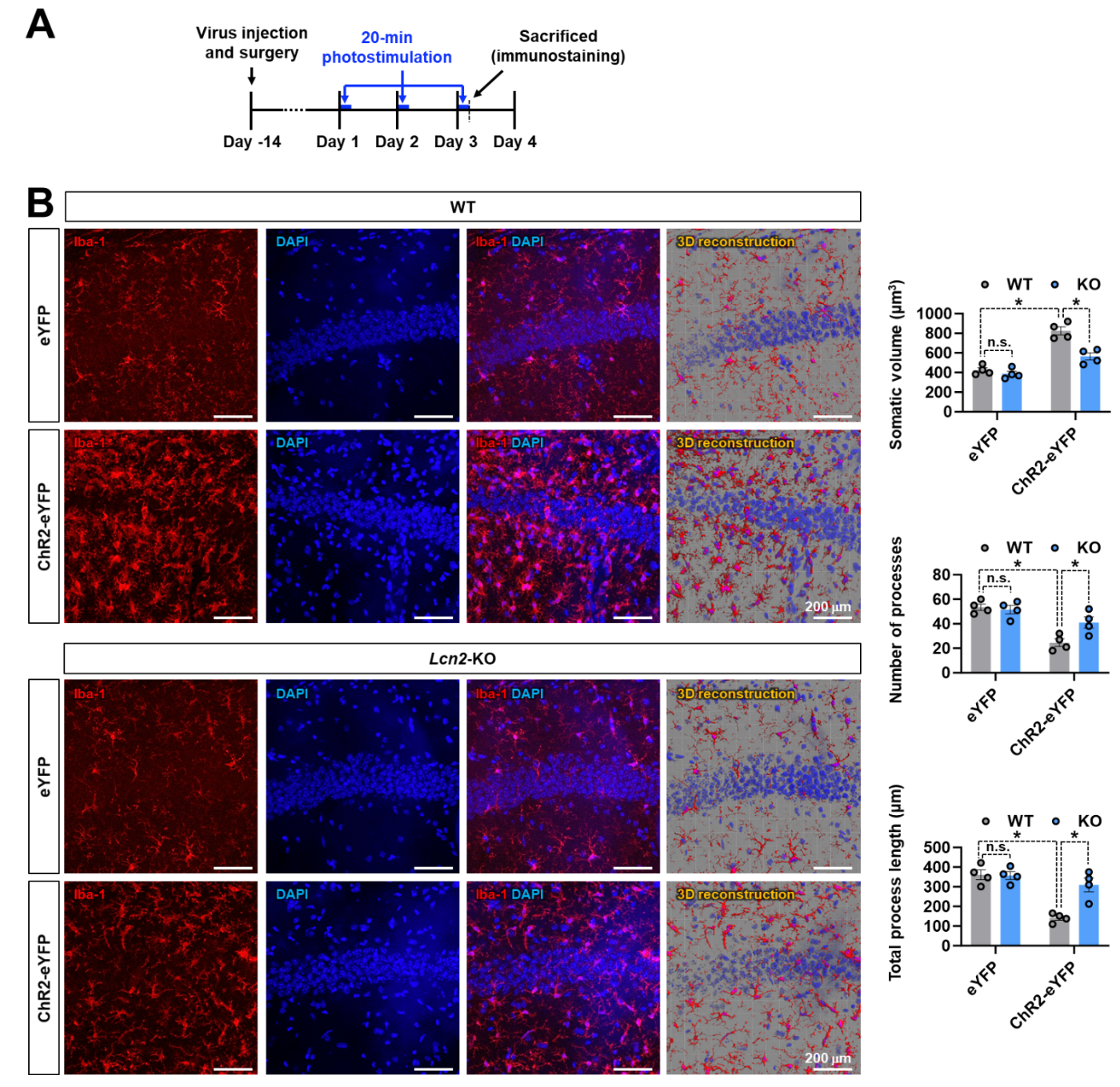

Supplement: S20 Fig — (A) Experimental timeline. (B) Hippocampal CA1 sections stained with anti-Iba-1 antibody and DAPI show various morphological changes (somatic volume, process number, and total process length) in microglia following optogenetic astrocyte stimulation. These morphological changes in microglia are attenuated by Lcn2 deficiency. Scale bar: 200 μm. Results are presented as mean ± SEM (n = 4). *p < 0.05 between the indicated groups; n.s., not significant (one-way ANOVA). Source data can be found in S1 Data. (TIFF) [file pbio.3002687.s020.tiff]

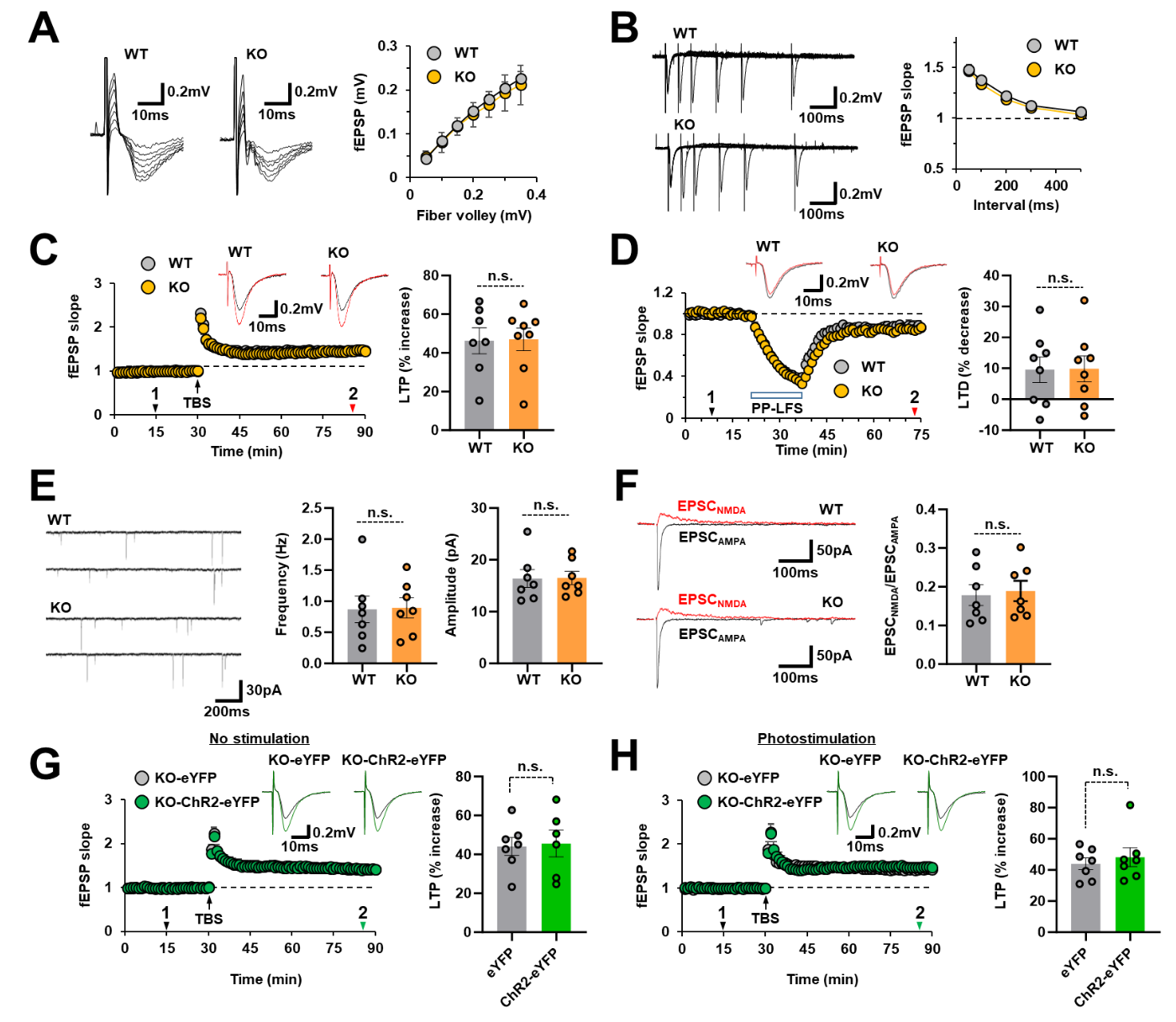

Supplement: S21 Fig — (A) Typical traces of the fEPSPs evoked by various strengths of stimuli in the WT (left) and Lcn2-KO (right) mice. To examine the input–output relationship, the fEPSPs were recorded in the presence of 3 μm CNQX to reduce the fEPSP amplitude. The relationship between the fiber volley amplitude (input) and fEPSP amplitude (output) in the WT (gray circles, n = 6 from 5 mice) and Lcn2-KO (yellow circles, n = 6 from 5 mice) mice. Results are expressed as mean ± SEM. (B) Typical traces of fEPSPs evoked by the paired-pulse stimuli with various intervals in the WT (upper) and Lcn2-KO (lower) mice. The paired-pulse ratio of fEPSPs in WT (gray circles, n = 6 from 5 mice) and Lcn2-KO (yellow circles, n = 6 from 5 mice) mice. Results are expressed as mean ± SEM. (C) Time courses of the fEPSP responses before and after the TBS from the hippocampal sections of the WT (gray circles, n = 7 from 5 mice) or Lcn2-KO (yellow circles, n = 8 from 5 mice) mice. The values were normalized in each experiment to the mean amplitude value measured during the control period (20–30 min). TBS was applied for the LTP induction at 30 min. Insets represent the typical raw traces from the average of 6 successive fEPSPs recorded at the time indicated by the arrowheads with numbered regions (1; black or 2; red). TBS-induced LTP in the WT (gray circles, n = 7 from 5 mice) or Lcn2-KO (yellow circles, n = 8 from 5 mice) mice. The mean fEPSP slope during 50–60 min after TBS was quantified as the LTP level. Results are expressed as mean ± SEM. n.s., not significant (unpaired t test). (D) Time courses of the fEPSP responses before and after PP-LFS from the hippocampal sections of the WT (gray circles, n = 8 from 5 mice) or Lcn2-KO (yellow circles, n = 8 from 5 mice) mice. PP-LFS was applied for LTD induction at 20 min. Insets represent the typical raw traces from the average of 6 successive fEPSPs were recorded at the time indicated by the arrowheads with numbered regions (1; black or 2; red). The values wer [file pbio.3002687.s021.tiff]

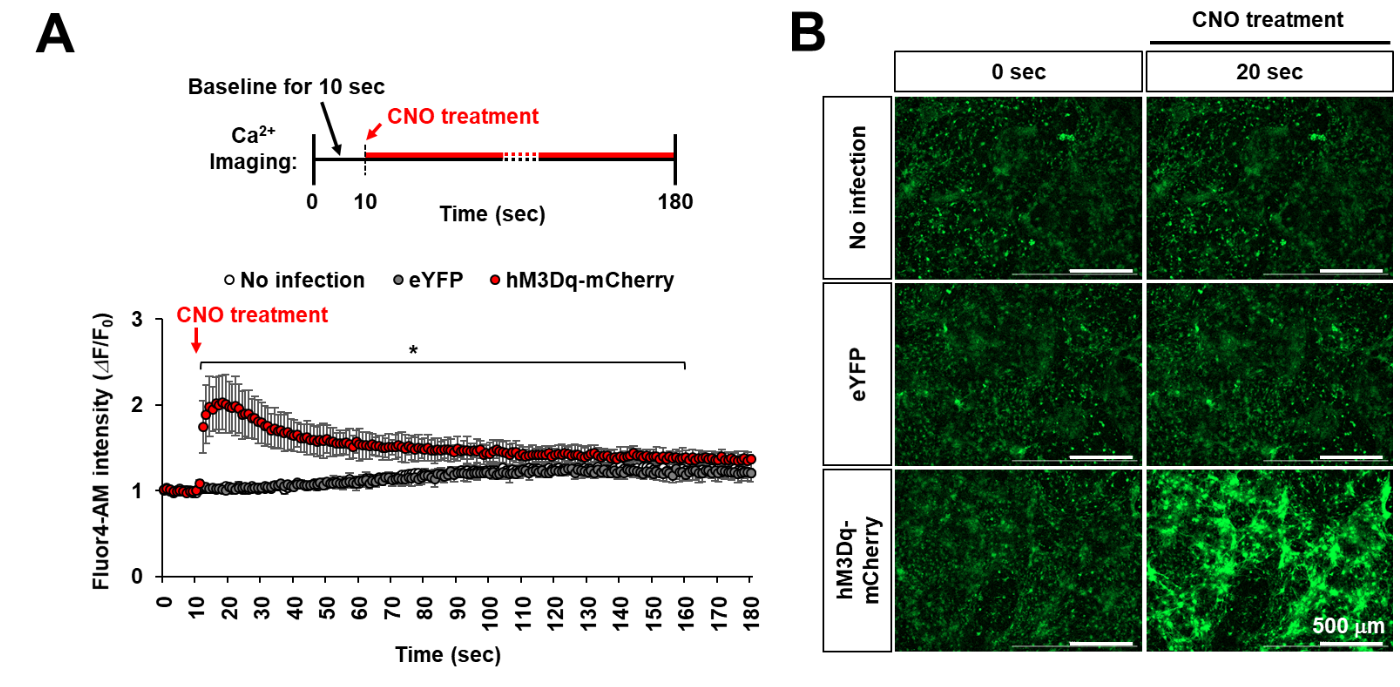

Supplement: S22 Fig — (A) Experimental timeline. The hM3Dq-mCherry expressing primary astrocytes loaded with Fluo-4 AM exhibited transient Ca2+ after treatment with CNO (10 μm). CNO was added at 10 s. Ca2+ levels in the non-infected or eYFP groups were not significantly altered. Results are expressed as mean ± SD (n = 3). *p < 0.05, eYFP versus hM3Dq-mCherry (10–160 s) (one-way ANOVA). (B) Representative images showing the Ca2+ transients. Scale bar: 500 μm. Source data can be found in S1 Data. (TIFF) [file pbio.3002687.s022.tiff]

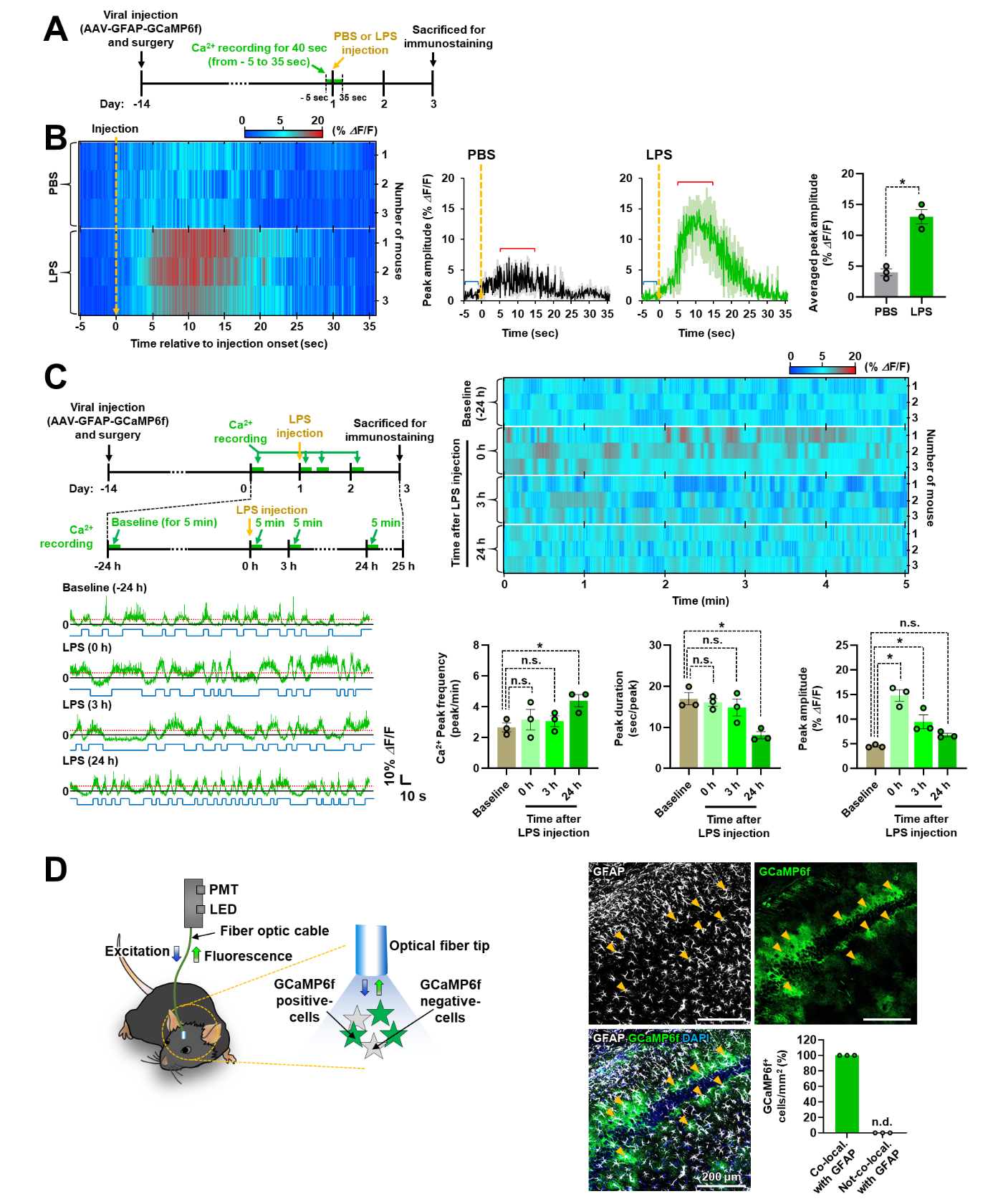

Supplement: S23 Fig — (A) Experimental timeline of virus injection to the hippocampal CA1 region, optical fiber implantation (for fiber photometry), and LPS injection (2 μg, i.c.v.). (B) Heatmap illustrates the GCaMP6f fluorescence (ΔF/F %) of the individual mice from −5 s to 35 s in response to PBS or LPS injection (yellow arrows and vertical dotted lines) (left). The plots illustrate the average fluorescence of 3 mice for each group (middle). The curves and shaded regions indicate the mean ± SEM (n = 3). The adjacent graph shows the averaged peak amplitude (right); average peak amplitude from 5 to 15 s (indicated by red line in the plot) was normalized to a baseline value from −5 to 0 s (indicated by blue line in the plot). Results are expressed as mean ± SEM (n = 3). *p < 0.05 between the indicated groups (one-way ANOVA). (C) Experimental timeline of GCaMP6f fluorescence recording in the hippocampal CA1 region after LPS administration. The heatmap and traces show the GCaMP6f fluorescence changes at baseline (at −24 h before LPS injection; recorded for 5 min) and after LPS injection (at 0, 3, and 24 h after LPS injection; recorded for 5 min). The plots show the ΔF/F (green trace), peak threshold (red dotted line), and the start and end of each peak that was detected (blue trace; lower). The adjacent graph displays the average peak frequency, duration, and amplitude before and after LPS administration. Results are expressed as mean ± SEM (n = 3). *p < 0.05 between the indicated groups; n.s., not significant (one-way ANOVA). (D) A schematic illustration of the experimental setup (left). Brain tissue samples were subjected to immunofluorescence analysis to localize the GCaMP6f (green) expression in astrocytes (GFAP, white) (right). The nuclei were stained with DAPI (blue). Arrowheads indicate the colocalization of GCaMP6f and GFAP. Quantification of GFAP and GCaMP6f colocalization is shown in the bar graphs. Scale bar: 200 μm. Results are expressed as mean ± SEM (n = 3). n.d., not detecte [file pbio.3002687.s023.tiff]
