# Supplementary material for: Aberrant activation of hippocampal astrocytes causes neuroinflammation and cognitive decline in mice
Source: PLoS Biol. 2024 Jul 11;22(7):e3002687. doi: 10.1371/journal.pbio.3002687 (PMC11239238; doi:10.1371/journal.pbio.3002687)
Supplement: S1 Table — (PDF) [file pbio.3002687.s024.pdf]

S1 Table. Comparison of current and previous studies using optogenetics or chemogenetics to manipulate astrocytes for interrogating cognitive function.

Target	Brain region	Stimulation condition (light/ CNO)	Effect (*in vivo* or *in vitro*)	Reactive astrocytes	Reference
**Optogenetics**					
ChR2 (H134R)	Hippocampus	20 min photostimulation once a day, repeated for three days (light on 500 ms, light off 500 ms)	Impaired short-term and long-term memory (Y-maze, passive avoidance, and Barnes maze)	Yes	Current study
ChR2 (H134R) in	Hippocampus	15 min photostimulation once a day (light on for 30 s, light off for 30 s)	Impaired contextual memory (contextual fear conditioning)	n.d	Li et al., 2020
ChR2 (H134R)	Hippocampal slice	90 s photostimulation (light on 1 s, light off 1 s)	Increased both IPSC and EPSC frequency (*in vitro*)	n.d.	Courtney et al., 2023
Gq-GPCR (Optoa1AR)	Hippocampal slice	90 s photostimulation (light on 1 s, light off 1 s)	No effect on IPSC and EPSC frequency (*in vitro*)		
Gq-GPCR (Optoa1AR)	Hippocampal slice	5 min photostimulation (light on 45 s, light off 5 s)	Increased IPSC and EPSC frequency (*in vitro*)		
Gq-GPCR (Optoa1AR)	Hippocampus	3 min photostimulation once a day (light on 45 ms, light off 5 ms)	Improved contextual memory (contextual fear conditioning)	n.d.	Adamsky et al., 2018
Gq-GPCR (Optoa1AR)	Anterior cortex	10 min photostimulation once a day (light on 3 s or 30 s, light off 3 min)	Enhanced long-term memory (novel object recognition)	No	Iwai et al., 2021
		25 min photostimulation once a day (light on 1 sec, light off 4 min 59 s)	Suppressed neuronal activity (*in vitro*)		
**Chemogenetics**					
Gq-GPCR (hM3Dq)	Hippocampus	CNO (3 mg/kg, 8-h interval, total 7 times over 3 days)	Impaired long-term memory (passive avoidance)	Yes	Current study
Gq-GPCR (hM3Dq)	Hippocampus	CNO (3 mg/kg, 30 min before the behavioral assays, once a day)	Enhanced spatial and contextual memory (novel arm preference and contextual fear conditioning)	n.d.	Adamsky et al., 2018
Gi-GPCR (hM4Di)	Hippocampus	CNO (10 mg/kg, 30 min before the behavioral assays, once a day)	Impaired remote contextual memory (contextual fear conditioning)	n.d.	Kol et al., 2020

Gi-GPCR (hM4Di)	Hippocampus	CNO (3 mg/kg, 8-h interval, total 7 times over 3 d)	Suppressed LPS-induced neuroinflammation and memory impairment (passive avoidance)	No	Kim et al., 2021

Abbreviations: IPSC, inhibitory postsynaptic current; EPSC, excitatory postsynaptic current; CNO, Clozapine N-oxide; n.d., not determined.
