# Supplementary material for: Aberrant activation of hippocampal astrocytes causes neuroinflammation and cognitive decline in mice
Source: PLoS Biol. 2024 Jul 11;22(7):e3002687. doi: 10.1371/journal.pbio.3002687 (PMC11239238; doi:10.1371/journal.pbio.3002687)
Supplement: S1 Raw Images — (PDF) [file pbio.3002687.s026.pdf]

Fig 2G

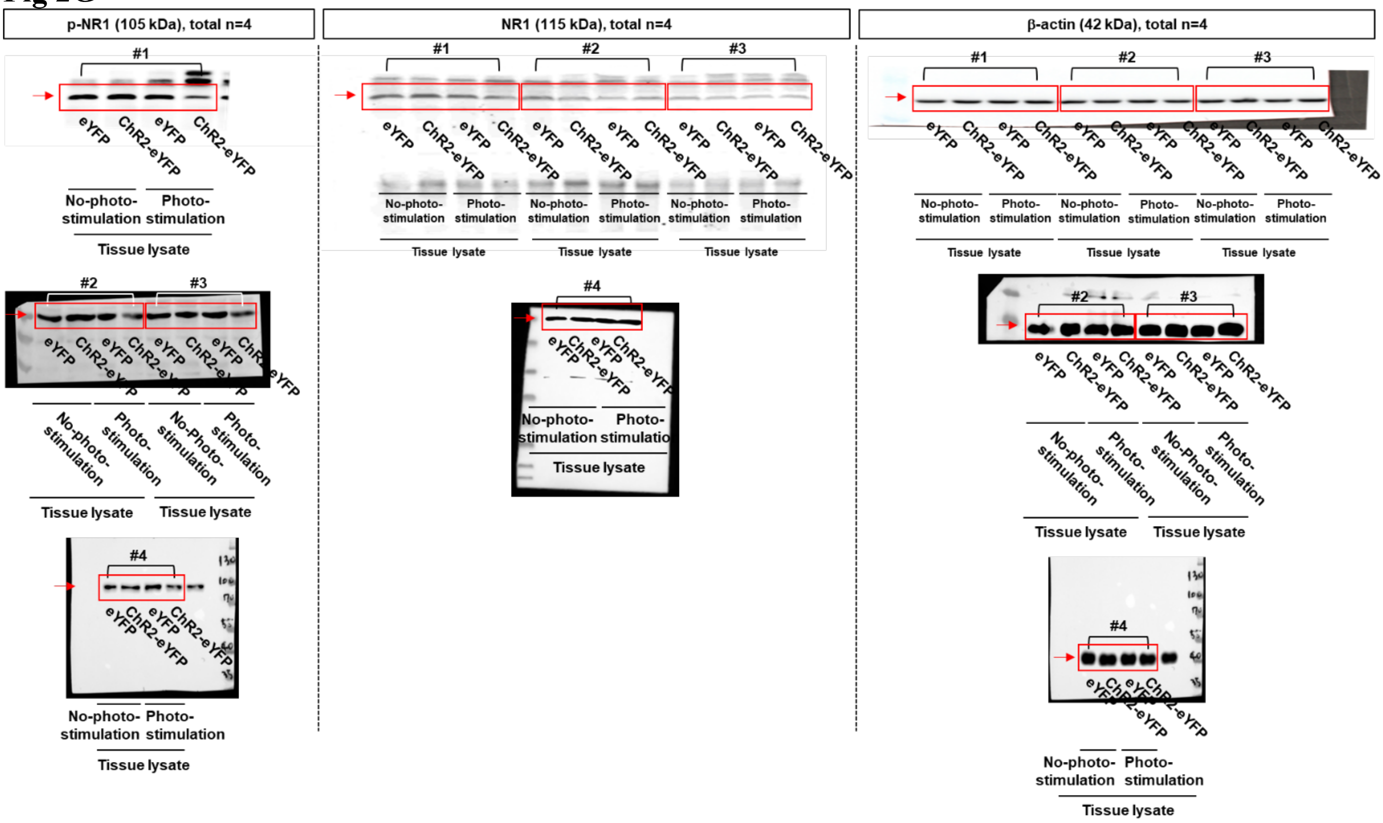
p-NR1 (105 kDa), total n=4
NR1 (115 kDa), total n=4
β-actin (42 kDa), total n=4
eYFP
ChR2-eYFP
No-photo-stimulation
Photo-stimulation
Tissue lysate

Fig 2H

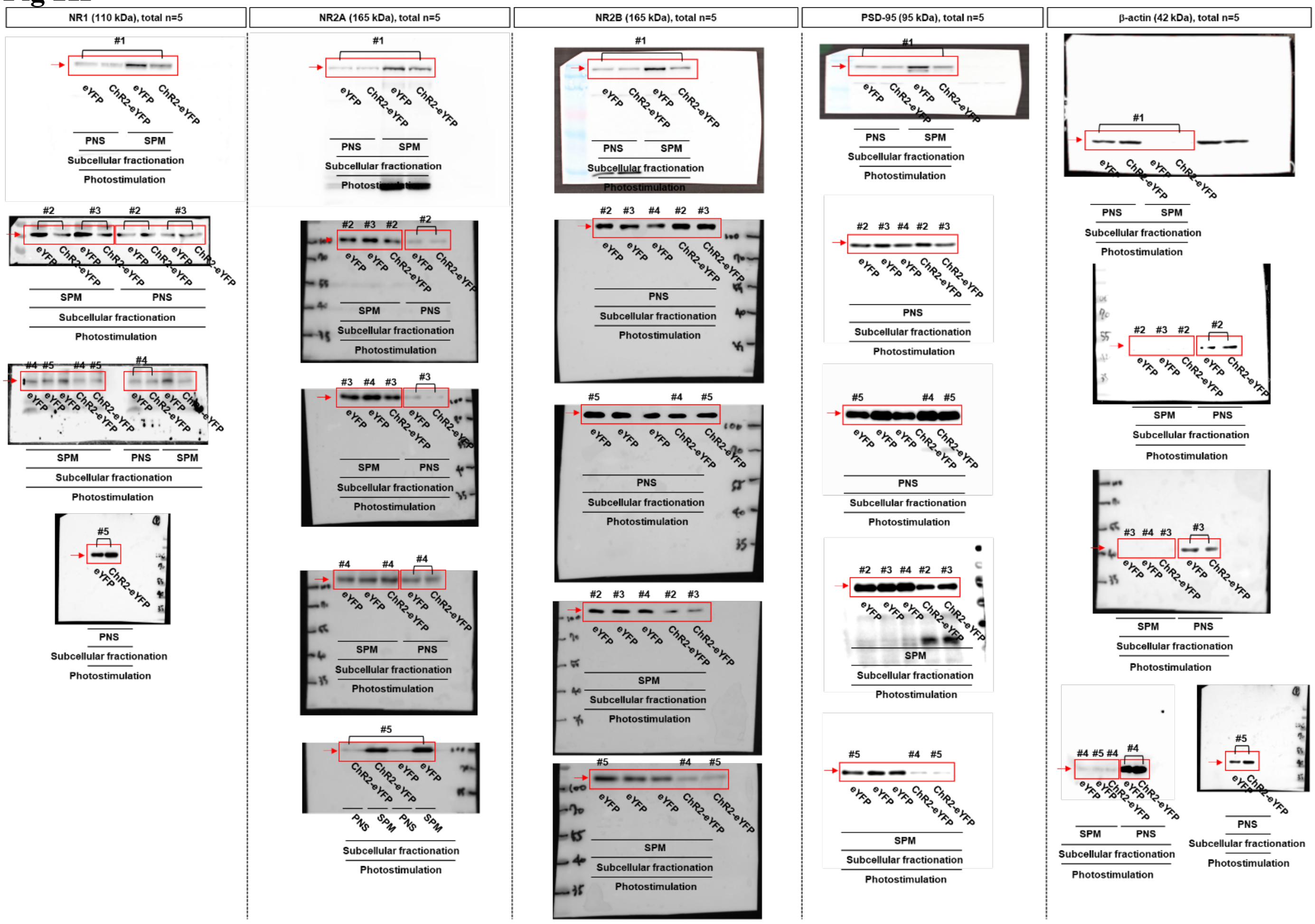
NR1 (110 kDa), total n=5
NR2A (165 kDa), total n=5
NR2B (165 kDa), total n=5
PSD-95 (95 kDa), total n=5
β-actin (42 kDa), total n=5
eYFP
ChR2-eYFP
PNS
SPM
Subcellular fractionation
Photostimulation

Fig 5B

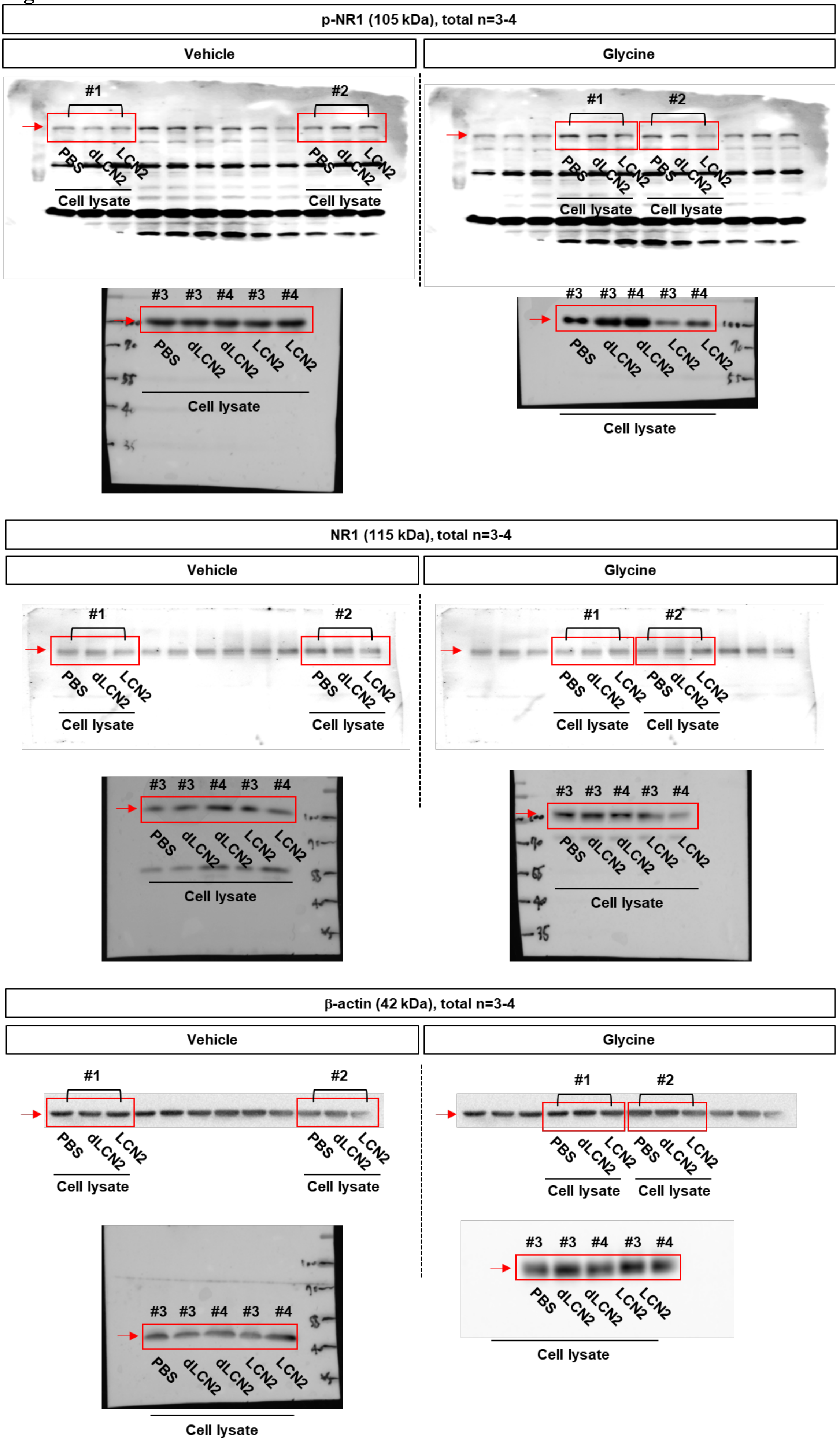
p-NR1 (105 kDa), total n=3-4
Vehicle
Glycine
#1
#2
PBS
dLCN2
LCN2
Cell lysate
#3
#4
NR1 (115 kDa), total n=3-4
β-actin (42 kDa), total n=3-4

Fig 5C

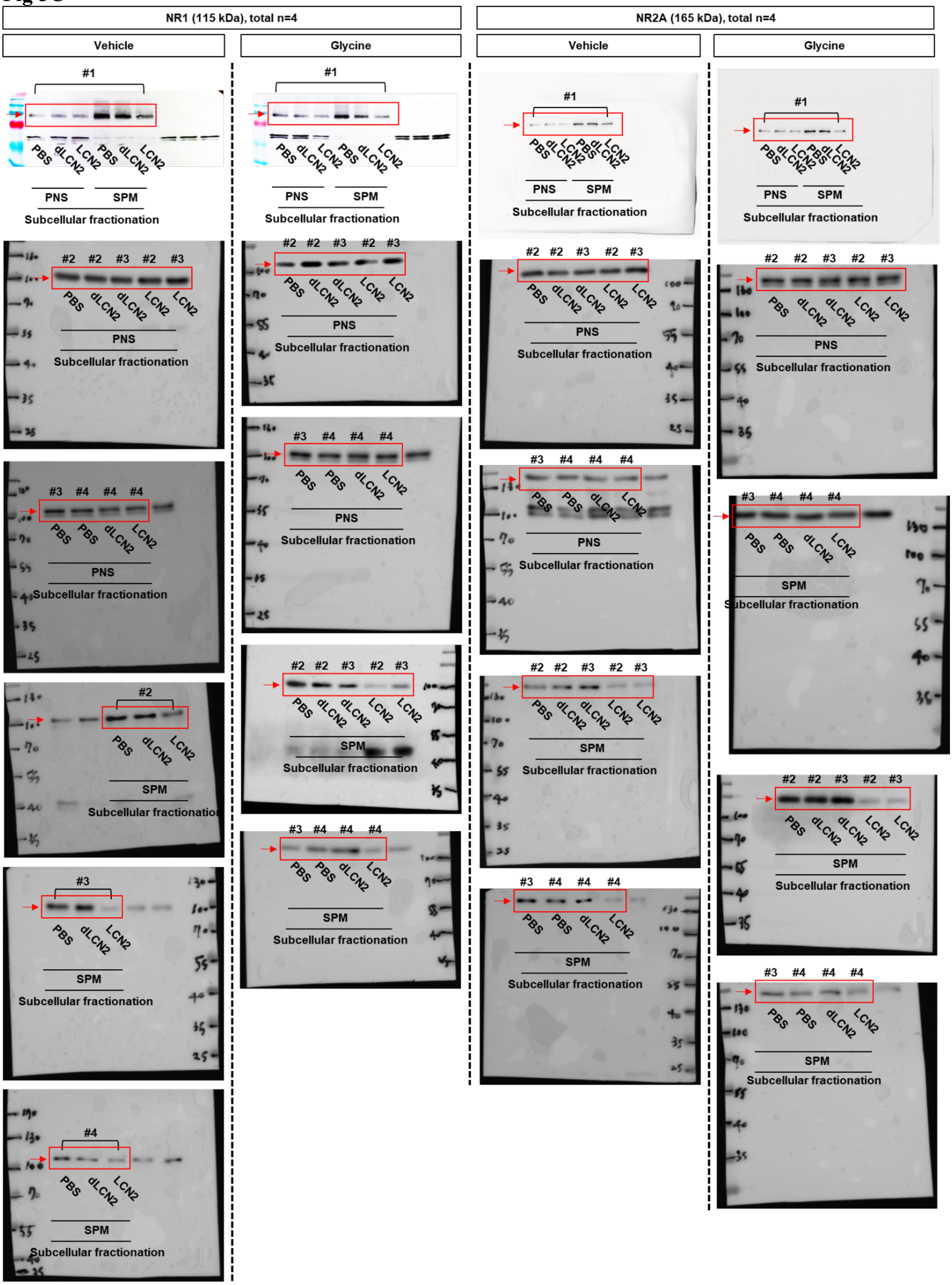
NR1 (115 kDa), total n=4
NR2A (165 kDa), total n=4
Vehicle
Glycine
Vehicle
Glycine
PNS
SPM
Subcellular fractionation

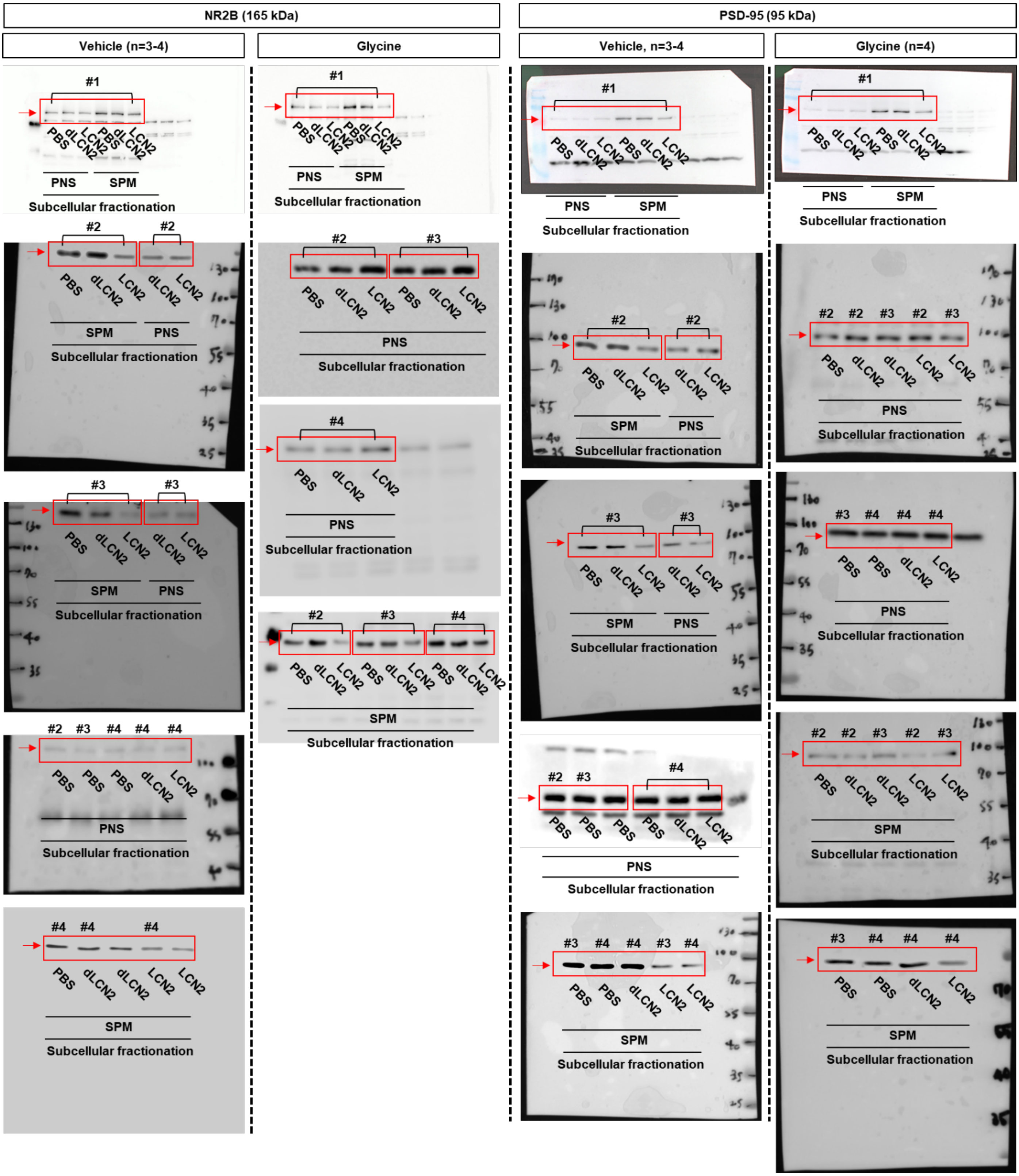
NR2B (165 kDa)
Vehicle (n=3-4)
Glycine
PSD-95 (95 kDa)
Vehicle, n=3-4
Glycine (n=4)
#1
PBS
dLCN2
LCN2
PNS
SPM
Subcellular fractionation
#2
#3
#4

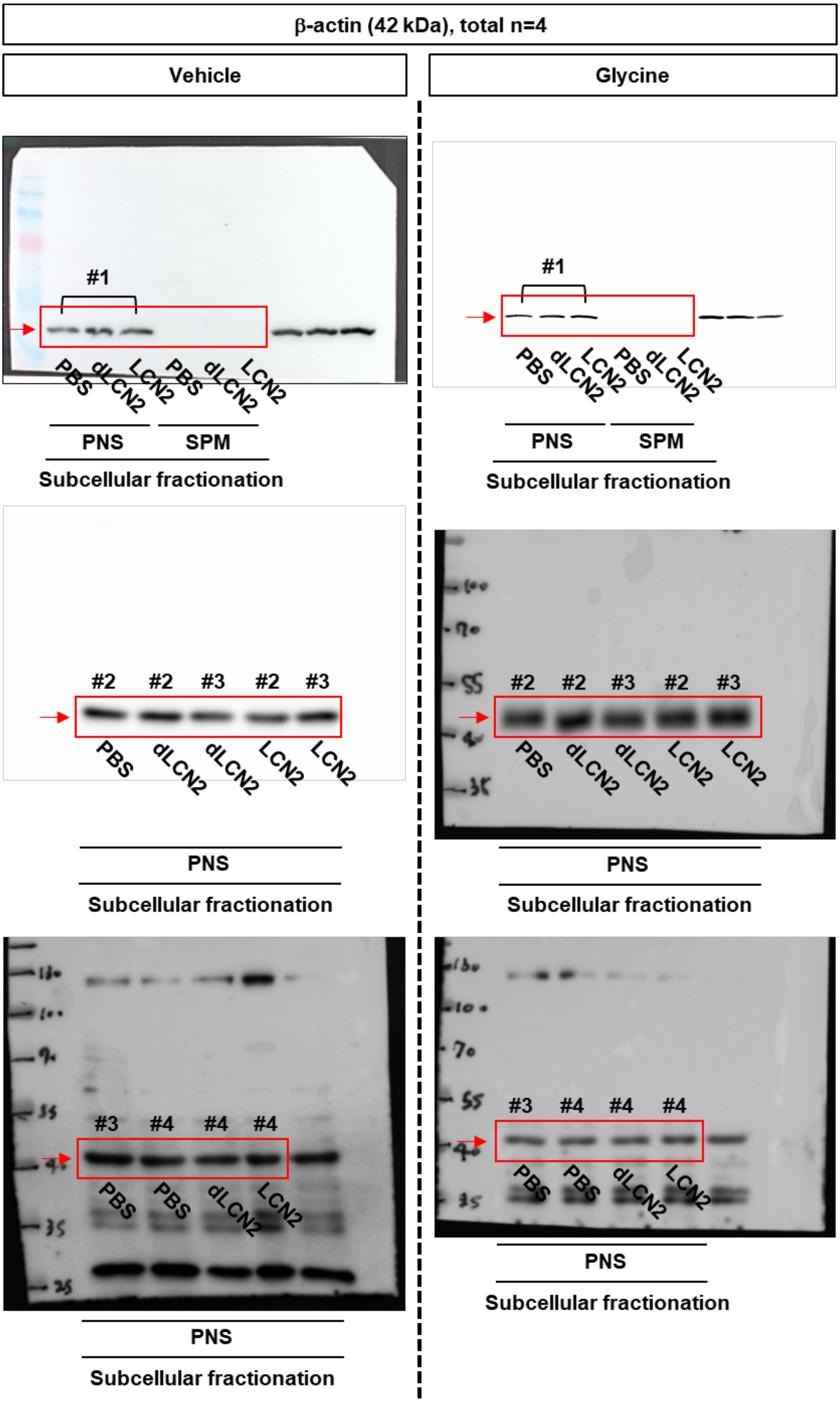
β-actin (42 kDa), total n=4
Vehicle
Glycine
#1
PBS
dLCN2
LCN2
PBS
dLCN2
LCN2
PNS
SPM
Subcellular fractionation
#1
PBS
dLCN2
LCN2
PBS
dLCN2
LCN2
PNS
SPM
Subcellular fractionation
#2
#2
#3
#2
#3
PBS
dLCN2
dLCN2
LCN2
LCN2
PNS
Subcellular fractionation
#2
#2
#3
#2
#3
PBS
dLCN2
dLCN2
LCN2
LCN2
PNS
Subcellular fractionation
#3
#4
#4
#4
PBS
PBS
dLCN2
LCN2
PNS
Subcellular fractionation
#3
#4
#4
#4
PBS
PBS
dLCN2
LCN2
PNS
Subcellular fractionation

S11B Fig

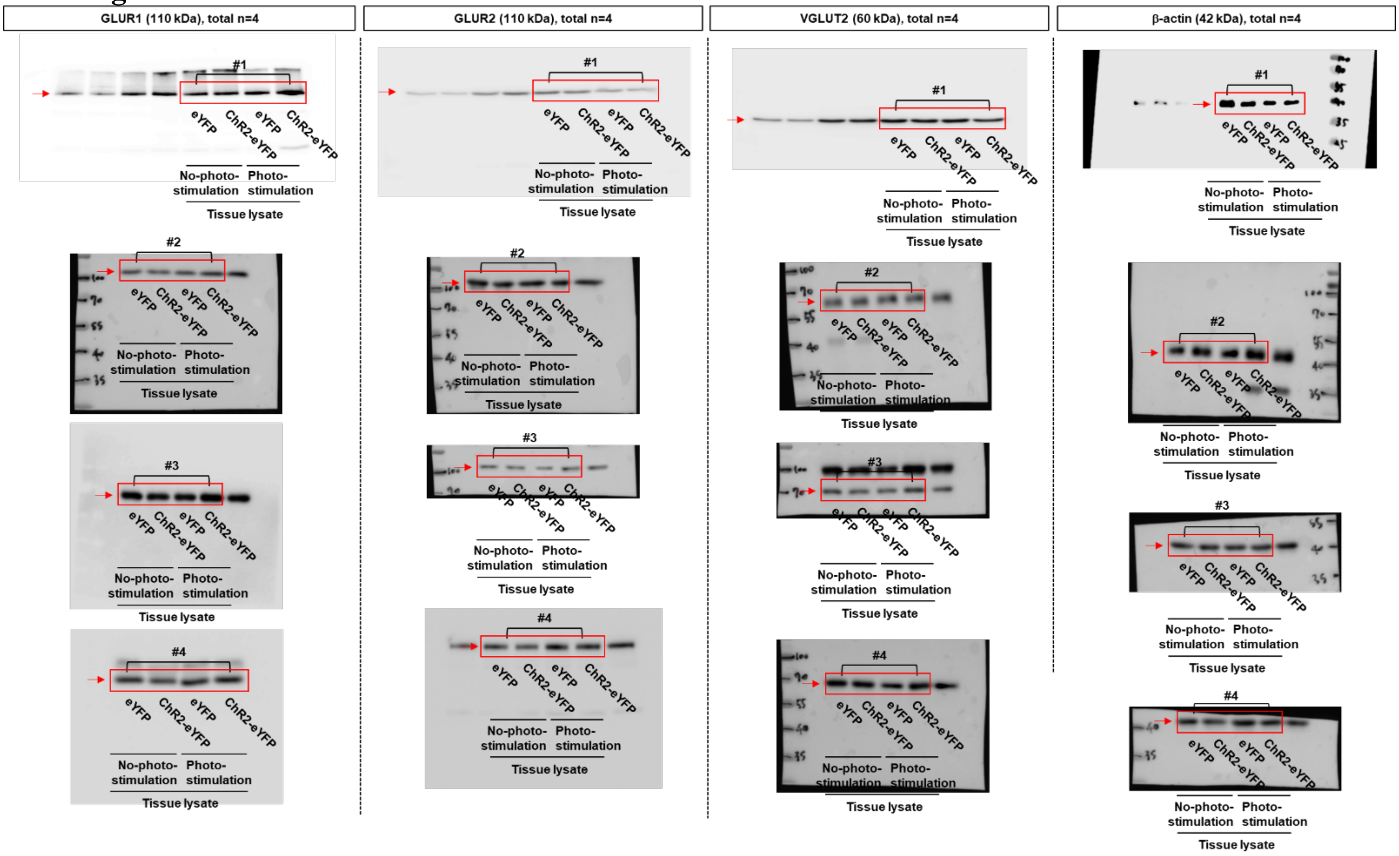

S11C Fig

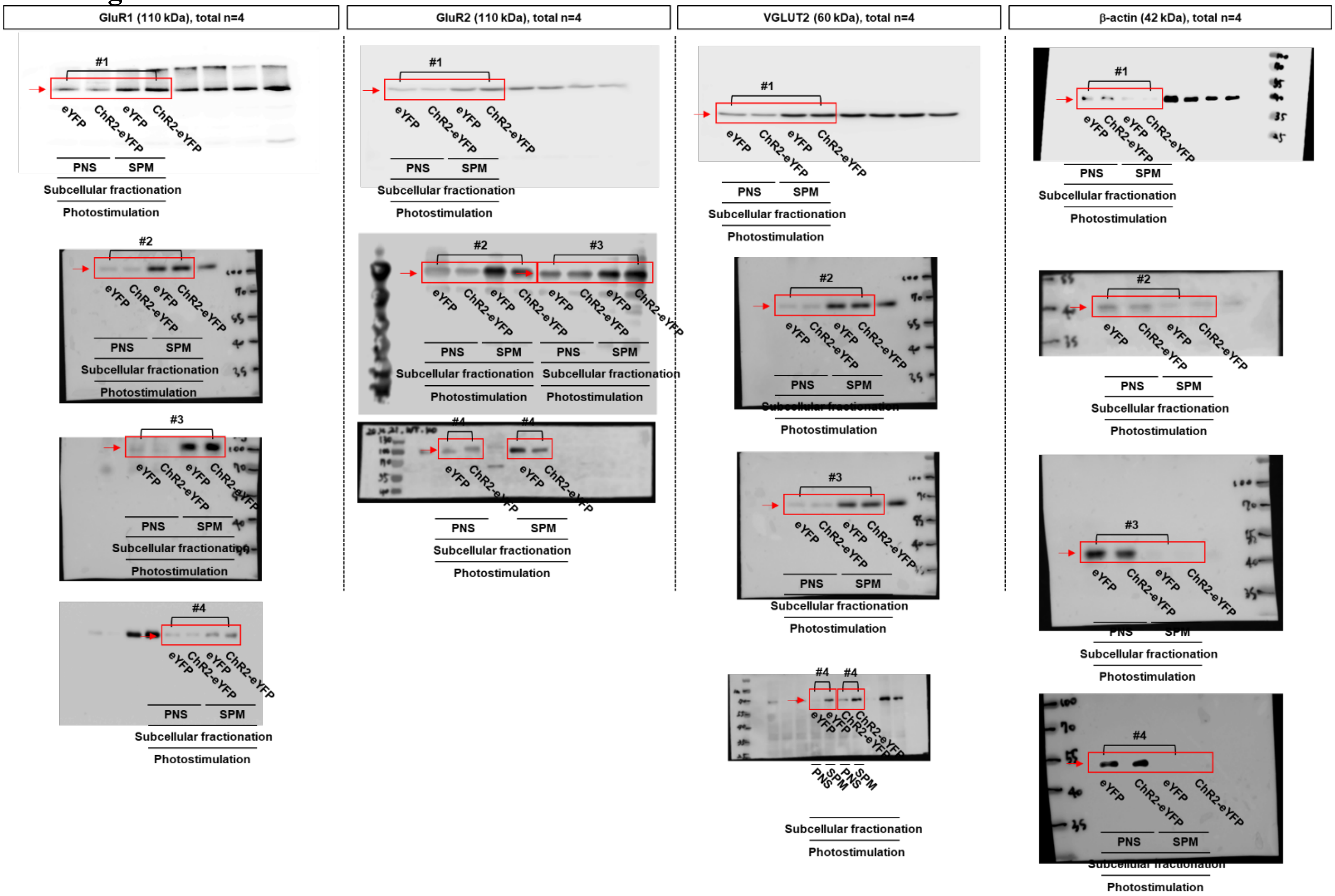

S12A Fig

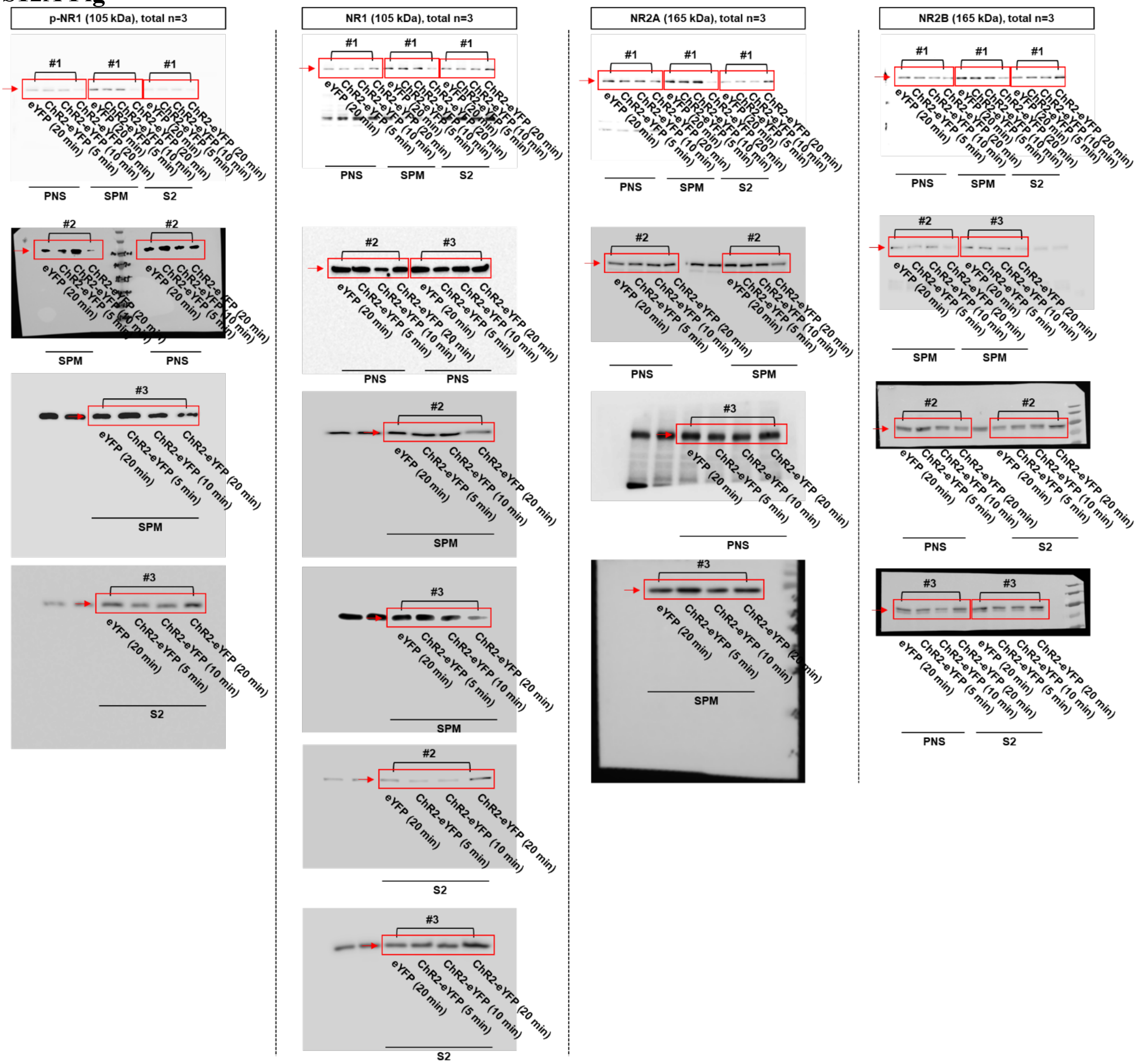

p-NR1 (105 kDa), total n=3
NR1 (105 kDa), total n=3
NR2A (165 kDa), total n=3
NR2B (165 kDa), total n=3
#1
#2
#3
eYFP (20 min)
ChR2-eYFP (5 min)
ChR2-eYFP (10 min)
ChR2-eYFP (20 min)
PNS
SPM
S2

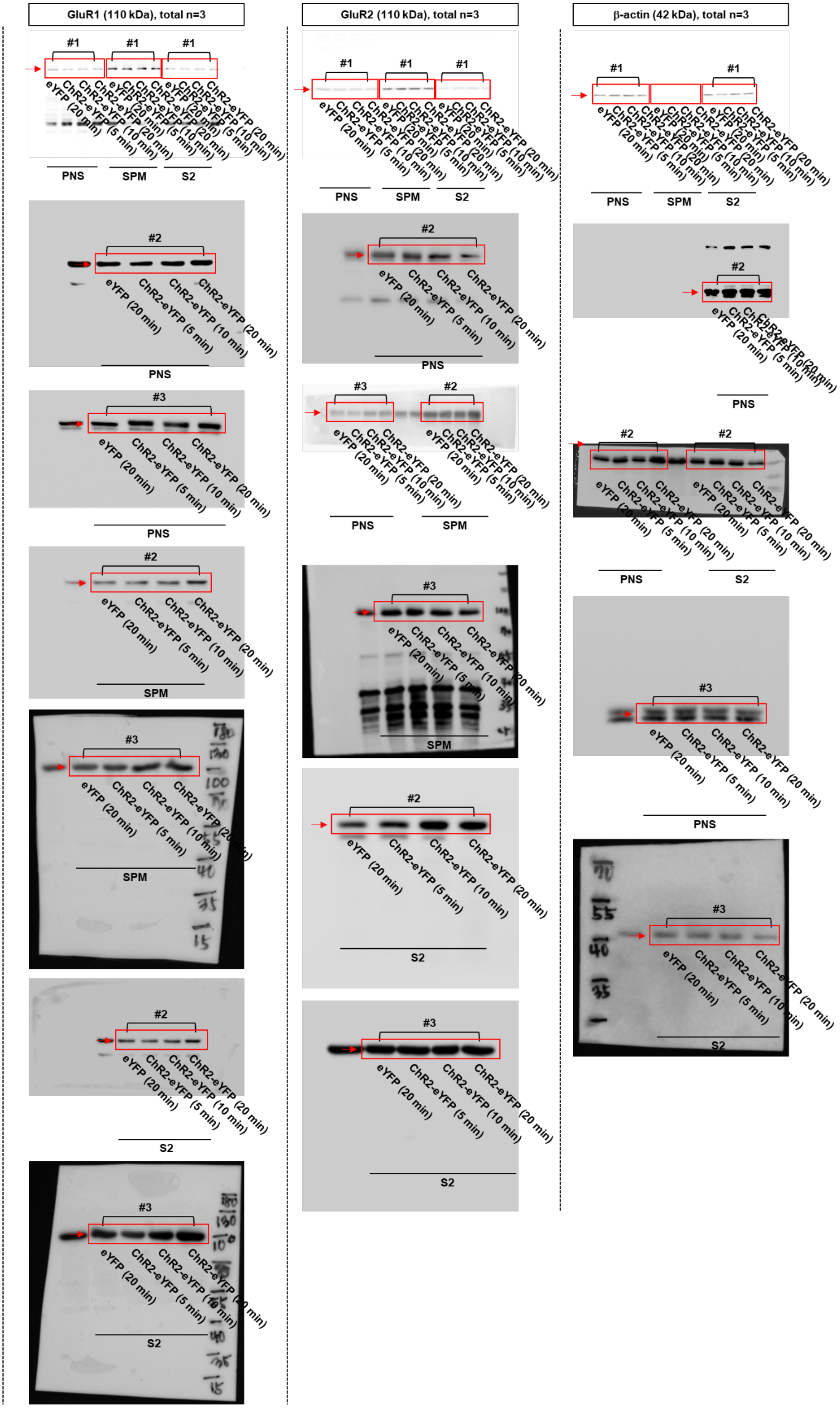
GluR1 (110 kDa), total n=3
GluR2 (110 kDa), total n=3
β-actin (42 kDa), total n=3
#1
#2
#3
eYFP (20 min)
ChR2-eYFP (5 min)
ChR2-eYFP (10 min)
ChR2-eYFP (20 min)
PNS
SPM
S2

S16B Fig (upper)

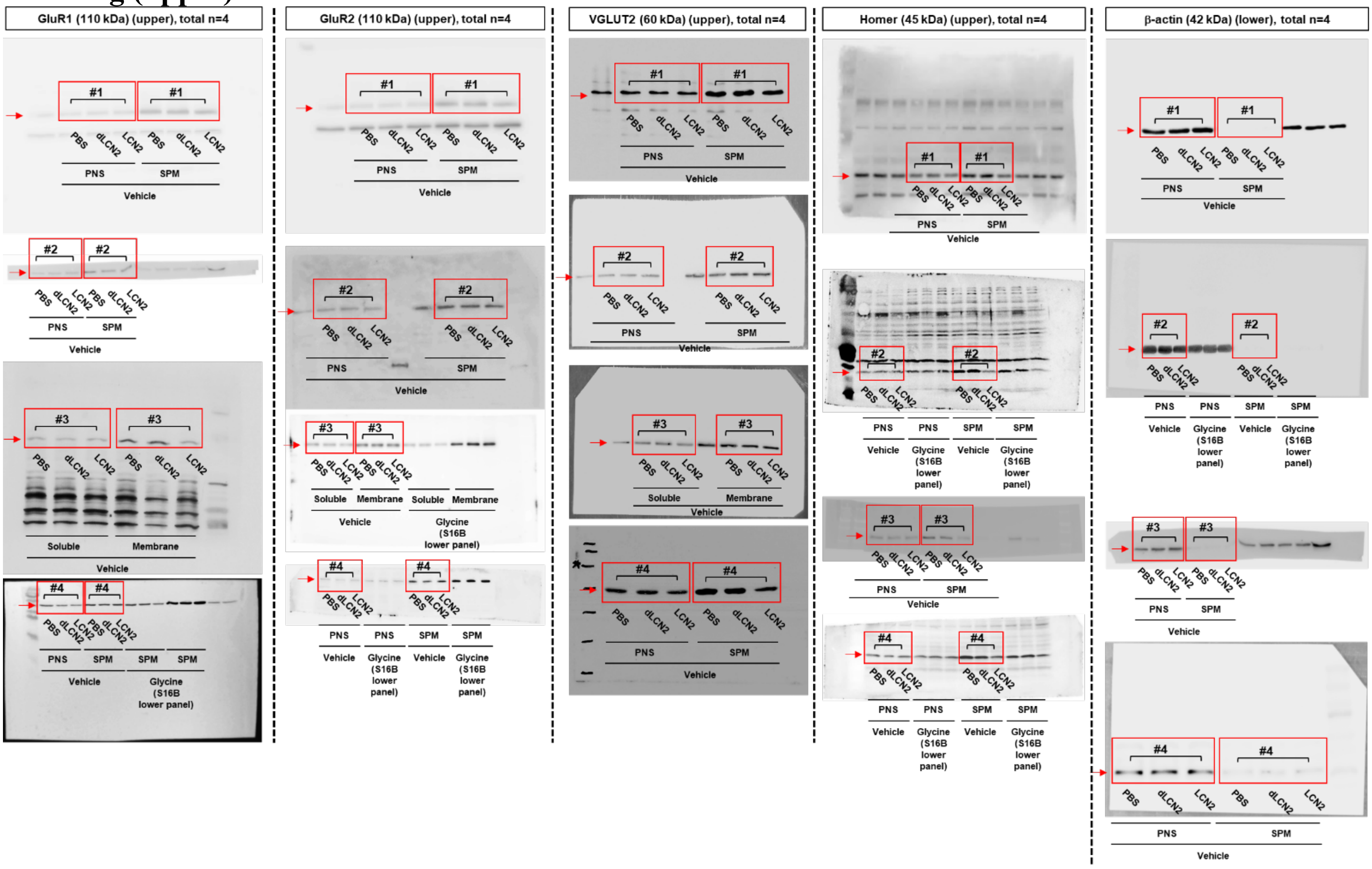

S16B Fig (lower)

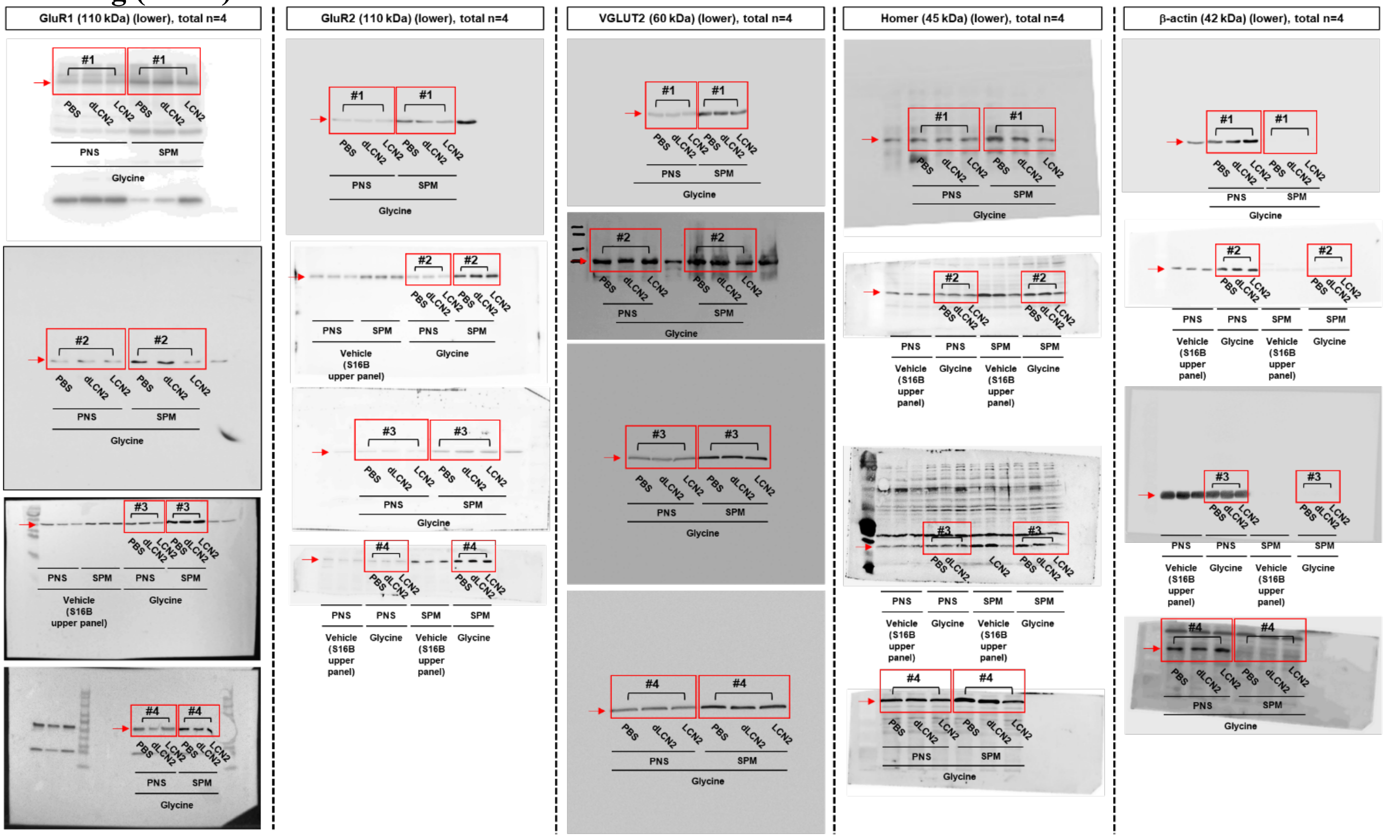

S16C Fig

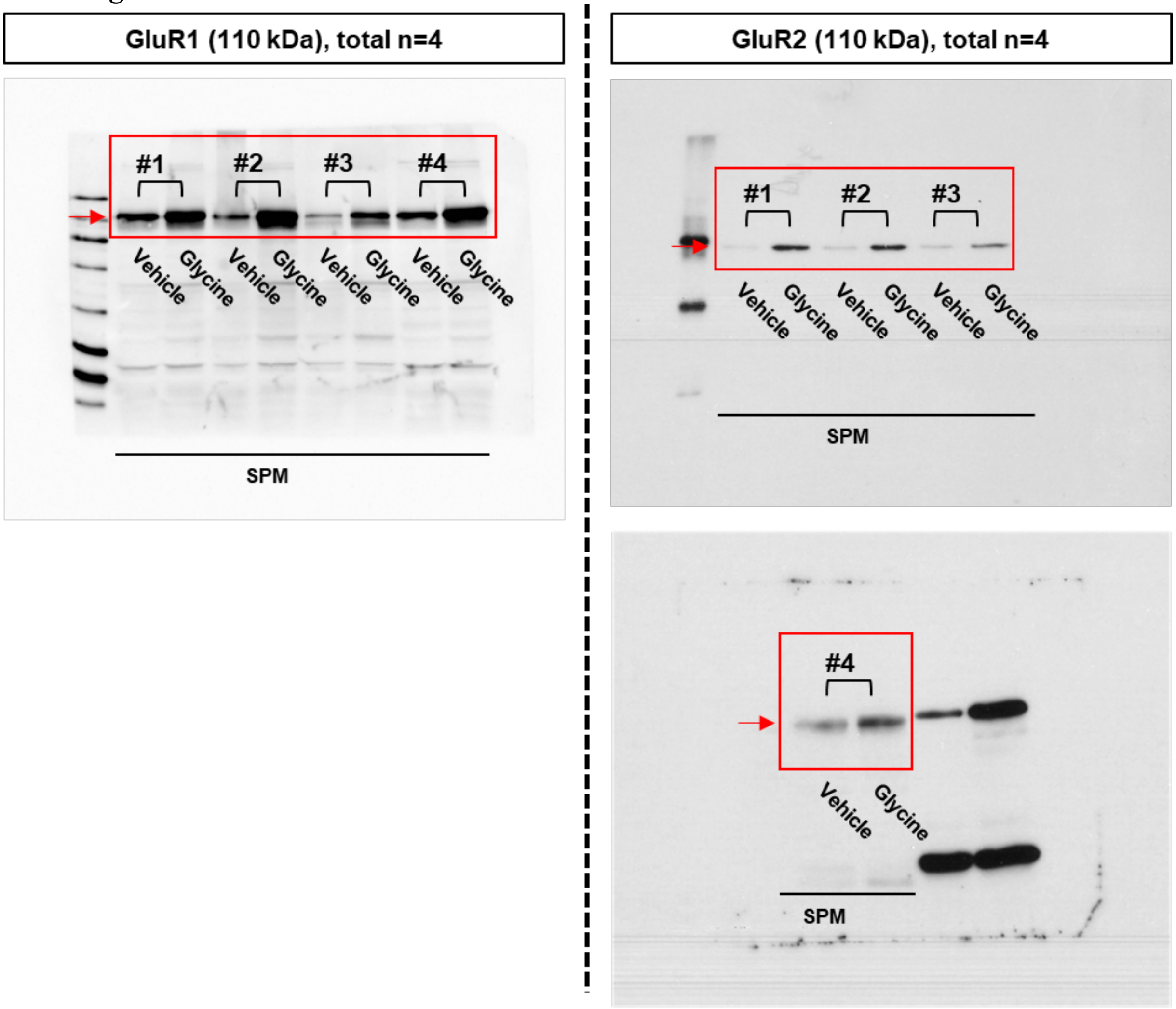
GluR1 (110 kDa), total n=4
#1
#2
#3
#4
Vehicle
Glycine
Vehicle
Glycine
Vehicle
Glycine
Vehicle
Glycine
SPM
GluR2 (110 kDa), total n=4
#1
#2
#3
Vehicle
Glycine
Vehicle
Glycine
Vehicle
Glycine
SPM
#4
Vehicle
Glycine
SPM
